# Human Atlas of Tooth Decay Progression: Identification of Cellular Mechanisms Driving the Switch from Dental Pulp Repair Toward Irreversible Pulpitis

**DOI:** 10.1002/advs.202510096

**Published:** 2025-10-31

**Authors:** Hoang Thai Ha, Sofya Kosmynina, Amandine Verocq, Keremsah Ozen, Ines Tekia, Hugo Bussy, Marie Ramirez, Dima Sabbah, Chloe Goemans, Valerie Vandenbempt, Esteban N. Gurzov, Sumeet Pal Singh, Nicolas Baeyens

**Affiliations:** ^1^ Laboratoire de Physiologie et Pharmacologie Faculty of Medicine Université Libre de Bruxelles (ULB) Brussels 1070 Belgium; ^2^ Signal Transduction and Metabolism laboratory Faculty of Medicine Université Libre de Bruxelles (ULB) Brussels 1070 Belgium; ^3^ Laboratory of Regeneration and Stress Biology Institut de Recherche Interdisciplinaire en Biologie Humaine et Moléculaire (IRIBHM‐Jacques E. Dumont) Université Libre de Bruxelles (ULB) Brussels 1070 Belgium

**Keywords:** dental pulp, fibrosis, inflammation, neurogenesis, odontoblasts, tooth decay, vascular remodeling

## Abstract

Dental pulp responses to dental decay, the most prevalent chronic disease worldwide, involve remodeling processes comparable to those observed in other human diseases. By combining volumetric imaging and single‐cell analysis at various stages of the disease in human samples, the natural history of how dental pulp responds to decay is uncovered. During the early phases, an arterialization of capillary networks and a progressive outward remodeling of larger vessels are observed. Additionally, neurogenesis of nerve endings and the reprogramming of perivascular progenitor cells into fibroblasts, initiating the physiological reparative response of the stromal tissue, is identified. Vascular and nerve regression, along with a shift in immune response and dental pulp fibrosis, contribute to irreversible pulpitis. These findings establish a foundation for a more comprehensive understanding of how dental tissues respond to injury, thereby prompting a paradigm shift in patient management strategies. Furthermore, this study underscores the potential of the human tooth as a valuable model for investigating other systemic diseases and evaluating treatment responses.

## Graphical abstract

Tooth decay progression transforms the dental pulp response from repair to fibrosis. At early stages, stromal cells reprogram to repair the extra cellular matrix (ECM), blood vessels, and nerves, remodel and grow, keeping repair possible. In advanced decay, hypoxia, and vessel regression, in complement with an immune switch, fuel nerve degeneration and tissue fibrosis, leading to irreversible damage.

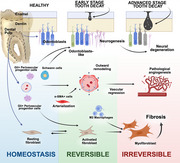

## 1 Introduction

The spatial and functional characteristics of cellular systems within the dental pulp, along with its heterogeneity and response to dental decay, remain largely underexplored due to the distinctive properties of the tooth. The dental pulp constitutes a specialized soft connective tissue located at the core of the tooth within a rigid, opaque, non‐expandable chamber. It is enclosed by hard, mineralized tissues: an outer enamel layer and an inner dentin layer containing dentinal tubules, which facilitate communication between the outer hard tissues and the pulp. The pulp exhibits a high degree of innervation and vascularization, with its vascular system playing a vital role in maintaining tooth homeostasis by supplying nutrients and oxygen and removing metabolic waste.^[^ ^1^ ^]^ Meanwhile, the neural system regulates blood flow, tooth sensitivity, and pain perception.^[^ ^2^ ^]^ Previous investigations utilizing resin casts^[^ ^3^ ^]^ and Indian ink^[^ ^4^ ^]^ in animal models have uncovered the fundamental 3D architecture of pulp vasculature. More recently, a study visualized the neurovascular systems of human dental pulp extracted from mineralized tissues following tissue clearing.^[^ ^1^ ^]^ However, these studies were either confined to animal models that do not naturally develop dental caries as observed in humans or involved pulp tissue extracted from its mineralized surroundings, often with limited imaging resolution. There exists a deficiency of high‐resolution visualization of the complete neurovascular architecture in human teeth, with preservation of the dentin‐pulp interface. The human tooth is distinctive in containing fully functional soft tissue that is accessible and readily collectible, for example, during wisdom teeth extraction, thereby enabling techniques such as omics investigations and therapeutic trials. Recent studies have examined the mouse and human dental pulp at the cellular level through single‐cell RNA sequencing (scRNAseq) analyses and have described the various cell populations that comprise the dental pulp.^[^ ^5^, ^6^, ^7^, ^8^ ^]^

Dental decay is a slowly progressing infectious disease and the most common chronic disease worldwide.^[^ ^9^ ^]^ It leads to enamel demineralization, followed by cavitation, and then the decay advances into the dentin until eventually reaching the pulp. This progression can be classified both clinically and histologically, as outlined by the SiSta (Site/stage) classification,^[^ ^10^ ^]^ which is based on the progression of decay within the hard tissue. The breakdown of hard tissues releases metabolic byproducts into the dentinal tubules, which can diffuse toward the dentin‐pulp interface. Pulpal cells detect invading pathogens and initiate a cascade of inflammatory responses known as pulpitis.^[^ ^11^ ^]^ Current clinical approaches mainly focus on removing infected hard tissues, without giving full weight to the pulp's regenerative capacity. The cellular and molecular mechanisms governing the pulp's response are still not fully understood, particularly in terms of how these processes change with disease progression and severity, and what drives the transition from healthy, reversible pulpitis to pathological, irreversible pulpitis. A pilot study has shown increased expression of pro‐inflammatory, anti‐inflammatory, and mineralization‐related genes in cases of deep decay.^[^ ^12^ ^]^ Another study highlighted the extent of extracellular matrix (ECM) remodeling within carious human teeth.^[^ ^13^ ^]^ A more thorough understanding of these mechanisms during disease progression is essential. Gaining precise insights into the cellular mechanisms that drive the transition from reversible pulpitis to irreversible pulpitis could lead to a paradigm shift in dental treatment, moving from traditional hard tissue removal and pulp cleansing via endodontic procedures to strategies focused on pulp preservation and regeneration.

In this study, we developed a comprehensive atlas of tooth decay in humans by combining volumetric imaging of cleared human teeth with a preserved dentin‐pulp interface and in‐depth single‐cell analysis of dental pulps in both healthy and diseased conditions. This approach provided new insights into the remodeling processes during disease progression, confirming or challenging previous observations. We identified a potential for reversibility in the early stages, with irreversible remodeling occurring in later stages if no dental treatment is provided. The remodeling processes, cellular mechanisms, and signaling cascades observed here are also seen in other systemic diseases, highlighting the human tooth as a potentially relevant model for studying systemic health conditions.

## 2 Results

### 2.1 Volumetric Imaging Unveils the Global Architecture of the Neurovascular Systems in the Healthy Human Dental Pulp: Innervation and Perfusion are Aimed at the Dentin–Pulp Interface

**Figure 1:**
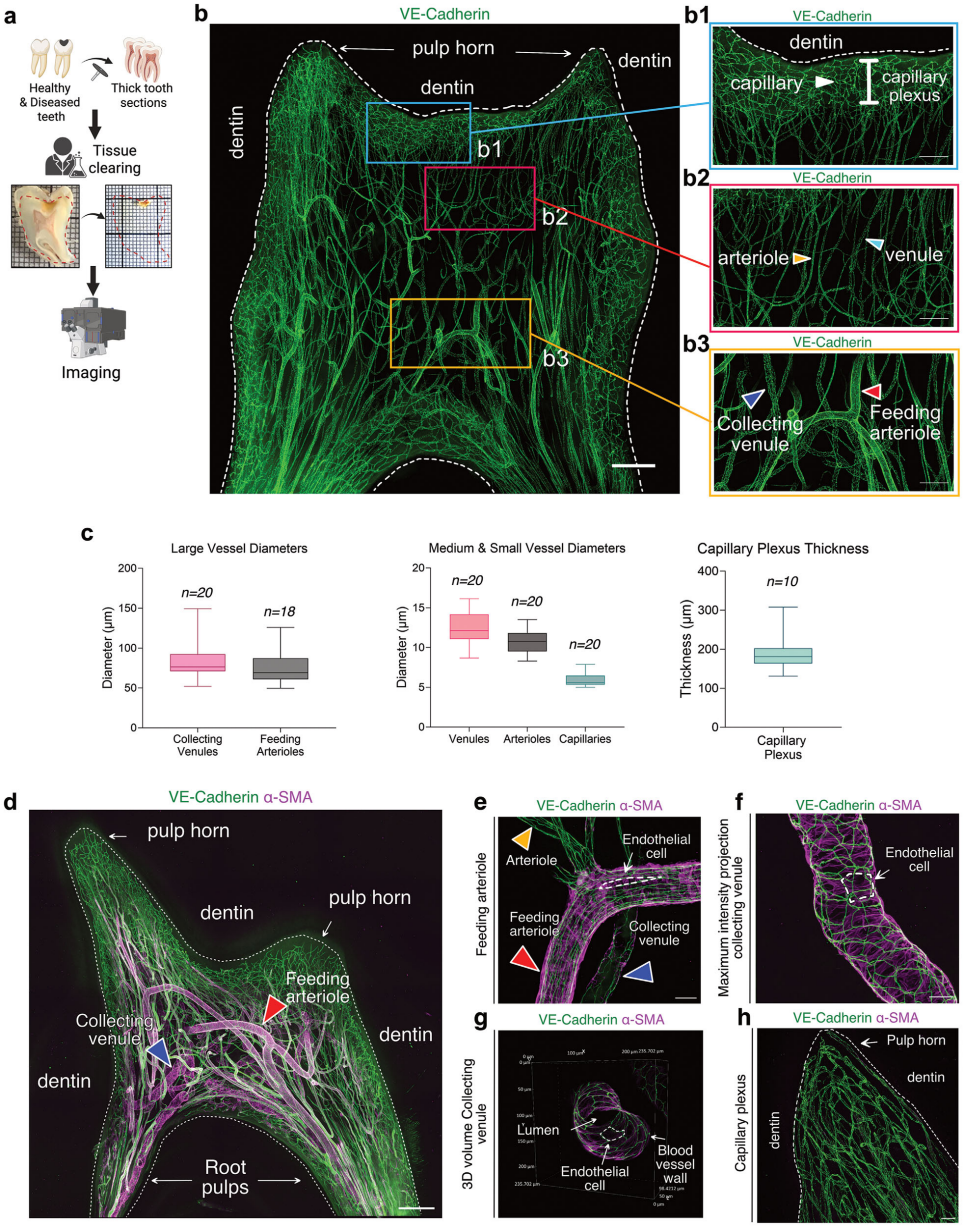
Global Architecture of the Vascular Systems in the Healthy Human Dental Pulp. a) Schematic of the imaging experimental workflow. b) Maximum intensity projection (MIP) of a 300 µm z‐stack showing the vasculature of a dental pulp in an immature molar, showing a spatial organization in three layers. b1) Zoom on the peripheral zone with capillaries organized in a dense plexus, with vessels mainly running parallel to the inner surface of the dentin. (white arrowhead) capillary. b2) Zoom on the intermediate zone with medium‐sized vessels showing a vertically oriented pattern. (orange arrowhead) arteriole, (turquoise arrowhead) venule. b3) Zoom on the central zone containing large vessels displaying a random orientation pattern. (blue arrowhead) collecting venule, (red arrowhead) feeding arteriole. Nikon confocal spinning disk microscope; immersion objective 10x (N/A 0.5 WD 5.5 mm; Nikon Plan Apo). Tissue clearing was performed using the iDISCO protocol, with staining for VE‐Cadherin (green). Scale bars: 500 µm for (b) and 200 µm for (b1, b2, b3). c) Plots show mean vessel diameters and capillary plexus thickness, with error bars indicating the range. ***n*** corresponds to the number of vessel measurements (typically 4–5 per sample, e.g. *n* = 20 from 4 samples, *n* = 18 from 4 samples). For capillary plexus thickness, ***n* = 10 samples** (one measurement per sample). Data are descriptive; no statistical tests were applied. (Left) Quantification of the mean diameters of large vessels. Collecting venules and feeding arterioles have mean diameters of 84.83 ± 24.12 µm (*n* = 20) and 76.08 ± 22.11 µm (*n* = 18), respectively. (Center) Mean diameters of medium and small vessels: venules, arterioles, and capillaries with diameters of 12.46 ± 2.05 µm (*n* = 19), 10.85 ± 1.48 µm (*n* = 20), and 5.89 ± 0.74 µm (*n* = 20). (Right) The capillary plexus has a mean thickness of 190.3 ± 46.64 µm (*n* = 10). d) MIP of a 343µm z‐stack showing the vasculature of a healthy mature dental pulp. (blue arrowhead) collecting venule, (red arrowhead) feeding arteriole. e) MIP of a 79 µm z‐stack displaying a feeding arteriole. (blue arrowhead) collecting venule, (red arrowhead) feeding arteriole, (orange arrowhead) arteriole. f) MIP of a 100 µm z‐stack showing a collecting venule. g) 3D projection of a 98 µm stack displaying a collecting venule. h) MIP of a 302 µm z‐stack showing capillaries, with a white dashed line indicating an endothelial cell. Tissue clearing was performed using a modified iDISCO protocol, with staining for the vasculature B–F) using VE‐Cadherin (green) and α‐SMA (purple). Point scanning resonant confocal microscope; large images for (d). Objectives used include Plan Apo 10x λS OFN25 DIC N1 for (d,f), and Plan Apo Lambda 25XC Sil for (g,e,h). Scale bars: 500µm for (b,d), 50 µm for (h), and 25 µm for (e,f). For Figure 1a, created in Biorender. HA. H. (2025) https://BioRender.com/q91e559. For all other panels, Copyright 2025, Hoang Thai HA.

**Figure 2:**
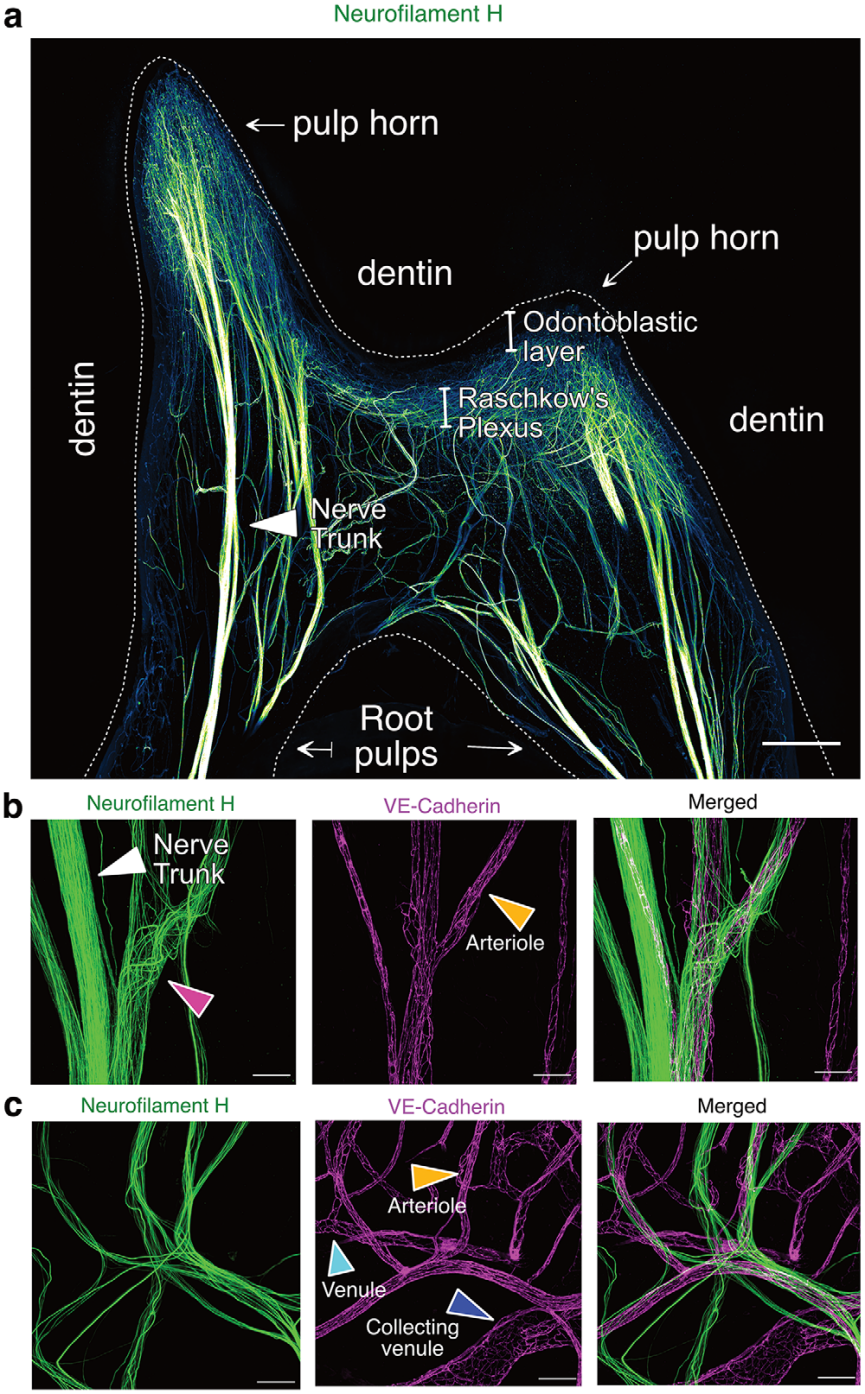
Global Architecture of the Neural Systems in the Healthy Human Dental Pulp. a) MIP of a 252 µm z‐stack showing the neural network of a healthy mature dental pulp. (white arrowhead) nerve trunk b) MIP of a 118 µm z‐stack and c) MIP of a 143 µm z‐stack showing the close neurovascular interactions. (white arrowhead) nerve trunk, (Pink arrowhead) nerve fibers change of orientation around a bifurcation; (orange arrowhead) arteriole; (turquoise arrowhead) venule, (blue arrowhead) collecting venule. Tissue clearing using a modified iDISCO protocol and staining for the neural network with Neurofilament‐H (green), and for the vasculature with VE‐Cadherin (purple). Point scanning resonant confocal microscope. Large images for (a). Plan Apo 10x λS OFN25 DIC N1 optic for (a,c). Plan apo Lambda 25XC Sil. Optic for (b). Scale bars: 500 µm for (a), 100 µm for (c), 50 µm for (b). Copyright 2025, Hoang Thai HA.

**Figure 3:**
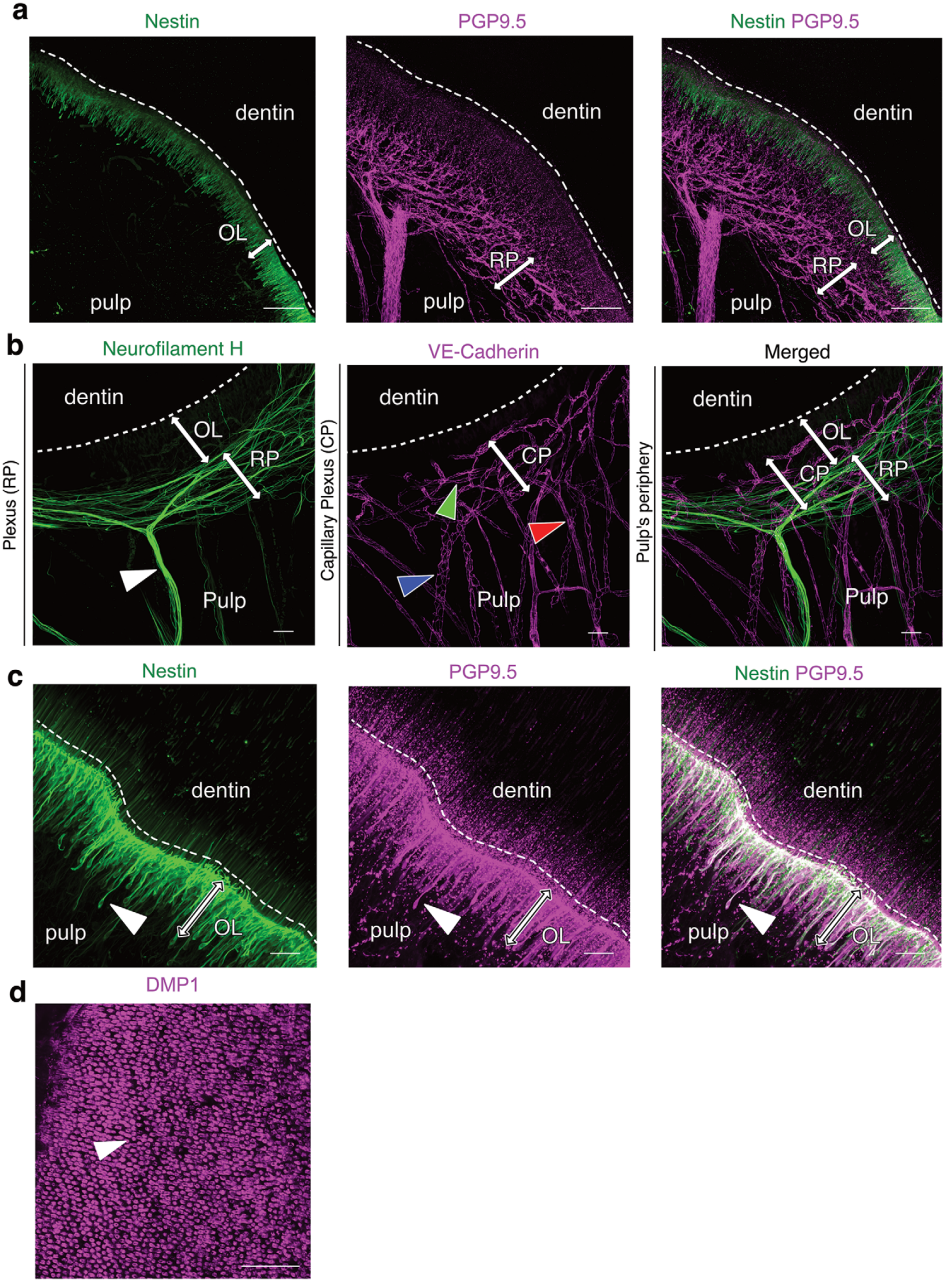
Architecture of the Peripheral Layer in the Healthy Human Dental Pulp. a) MIP of a 128 µm z‐stack showing the Raschkow's plexus (PGP9.5 staining) and the odontoblastic layer (nestin staining). (OL) odontoblast layer, (RP) Raschkow's plexus. b) MIP of a 143 µm‐stack showing the neurovascular interaction at the periphery of the pulp. (white arrowhead) nerve trunk, (OL) odontoblast layer, (RP) Raschkow's plexus, (blue arrowhead) venule; (red arrowhead) arteriole; (green arrowhead) capillary. c) MIP of a 91 µm z‐stack showing the odontoblastic layer. (OL) odontoblast layer, (white arrowhead) odontoblast body. d) MIP of an 18 µm z‐stack showing the dentinal tubules (DMP1 staining). (white arrowhead) dentinal tubule in a cross‐sectional view. Tissue clearing using a modified iDISCO protocol and staining for the vasculature (B‐F) with VE‐Cadherin (purple), for the neural network with Neurofilament‐H (green) and PGP9.5 (purple), for the odontoblastic cells (K) with Nestin (green and purple), and for the dentinal tubules with DMP1 (purple). Point scanning resonant confocal microscope. Plan Apo 10x λS OFN25 DIC N1 optic for (b, e). Plan apo Lambda 25XC Sil. Optic for (a, c, d). Scale bars: 100 µm for (a, e), 50µm for (b, d), 25 µm for (c). Copyright 2025, Hoang Thai HA.

To investigate the spatial characteristics of the human dental pulp, we collected samples from healthy (*n* = 40) and diseased (*n* = 74) teeth. These samples were processed into thick sections of >1mm, preserving the dentin‐pulp interface. The sections were cleared using a modified iDISCO protocol,^[^ ^14^ ^]^ immunolabeled, and imaged across the entire surface of the pulp at high resolution with a confocal microscope, by merging several z‐stacks (**Figure** **1**a). The dental pulp shows a dense, well‐organized vasculature (Figure 1b). High‐resolution 3D volumetric images of blood vessels and maximum intensity projections (MIP) from z‐stacks were created. We identified five distinct blood vessel types based on size, location, endothelial cell (EC) alignment, and mural cell (smooth muscle cells (SMCs) and pericytes) coverage: collecting venules, venules, feeding arterioles, arterioles, and capillaries. Feeding arterioles from the tooth roots converge centrally, branching into vertical arterioles that supply a dense capillary plexus beneath the dentin‐pulp interface. Blood flows through vertical venules into larger collecting venules, which exit via the roots (Figure 1b). Small vessels (capillaries) lie in the periphery of the pulp and form the capillary plexus (Figure 1b‐1), covering an intermediate zone made up of medium‐sized vessels oriented vertically (precapillary arterioles and postcapillary venules) (Figure 1b‐2). Larger vessels (feeding arterioles and collecting venules) are located centrally (Figure 1b‐3). Their mean diameters and the capillary plexus thickness have been quantified. (Figure 1c). ECs, marked by vascular endothelial (VE) cadherin‐specific antibodies targeting an endothelial‐specific adhesion molecule found at EC junctions,^[^ ^15^ ^]^ respond to blood flow forces called blood flow‐induced, fluid, or wall shear stress,^[^ ^16^, ^17^ ^]^ which affect their number, shape, and alignment.^[^ ^18^ ^]^ Arterioles experience higher shear stress (10–70 dynes cm^−^^2^), promoting EC elongation (Figure 1e), while venules, with lower stress (1–6 dynes cm^−^^2^), develop a cobblestone‐like morphology (Figure 1f,g).^[^ ^17^, ^18^ ^]^ Structurally, arterioles have thick tunica media layers with a continuous SMC layer, whereas venules have thinner, less resistant walls, with discontinuous coverage of SMCs. Capillaries, made up of a single EC layer with no tunica media, form a dense plexus at the dentin‐pulp interface, underscoring their essential role in pulp vascularization (Figure 1h). Mural cells were identified using antibodies targeting α‐smooth muscle actin (α‐SMA).^[^ ^19^ ^]^

The presence of lymphatic vessels within the dental pulp has been a subject of debate for many years.^[^ ^20^ ^]^ In our investigation, immunostaining utilizing lymphatic‐specific antibodies such as *LYVE1*, *PROX1*, and D2‐40 (Podoplanin) were used, but did not demonstrate the existence of a distinct lymphatic network within healthy dental pulp (Figure S1, Supporting Information).

Nerve fibers were immunolabeled either with Neurofilament H (NF‐H)‐specific antibodies or with PGP9.5‐specific antibodies,^[^ ^21^ ^]^ revealing a dense network. Large nerve trunks enter the root apex, traverse the roots, and branch extensively in the pulp chamber before reaching the periphery (**Figure** **2**a). Neurovascular interactions, well‐described in peripheral tissues^[^ ^22^ ^]^ and dental pulp,^[^ ^23^ ^]^ involve unmyelinated C‐fibers (regulating blood flow, dull pain, and running along blood vessels) and myelinated A‐fibers (mediating tooth sensitivity, sharp pain, and running independently) (Figure 2b,c; Video S1, Supporting Information).^[^ ^23^ ^]^

At the periphery near the dentin‐pulp interface, nerve trunks form a dense Raschkow's plexus (RP) beneath the odontoblastic layer (OL), closely interacting with the capillary plexus (CP) (**Figure** **3**a,b). Occasionally, some nerve fibers extend beyond the RP, crossing the OL to penetrate the dentin through dentinal tubules (Figure 3b). Odontoblasts, specialized cells at the pulp periphery, reside in the OL with their cell bodies next to the dentin. Their processes extend into the dentin via tubules to different depths (Figure 3c,d). Odontoblasts are essential, as they produce secondary and tertiary dentin in response to stimuli or injury, and facilitate pulpal sensory functions through molecular crosstalk with nerve fibers. The mechanisms behind these interactions are not yet fully understood. The dense CP just under the OL provides metabolic support, which is vital for these functions.

### 2.2 Cellular Heterogeneity of the Human Dental Pulp

**Figure 4:**
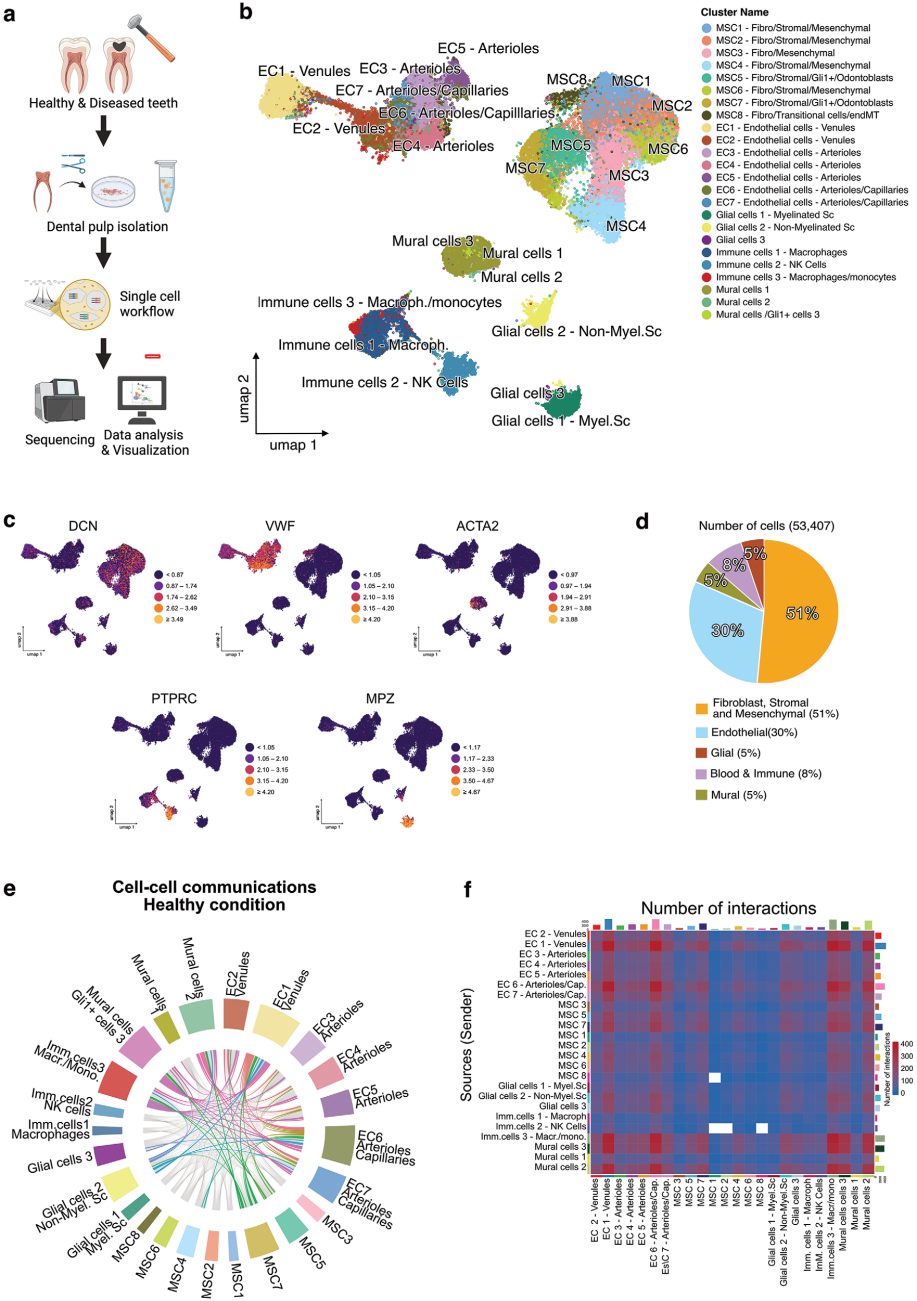
Cellular Heterogeneity of the Human Dental Pulp. a) Schematic of the single‐cell experimental workflow. b) Uniform manifold approximation and projection (UMAP) representation of 53.407 filtered and batch corrected healthy and diseased dental pulp cells across four healthy pulps, four early‐stage and four advanced‐stage diseased pulps. 24 clusters have been identified. c) Relative expression of a well‐established cell type marker for each cell population projected on UMAP plots. *DNC* for fibroblast and stromal cells; *VWF* for ECs; *ACTA2* for mural cells; *PTPRC* for immune cells; *MPZ* for Glial cells. d) Proportions of each cell population. e) Chord diagram showing inferred ligand–receptor interactions among all cell populations but restricted to the Healthy condition, as inferred by CellChat. Strong connections are observed between mesenchymal stem cell (MSC) subsets, endothelial subclusters (including venules, arterioles, and capillaries), mural cells, glial populations, and immune cells. f) Heatmap summarizing the total number of inferred interactions between sender (rows) and receiver (columns) cell populations. MSC subsets, ECs, and mural cells engage in the highest number of interactions. At the same time, glial and immune populations also contribute to the overall signaling network, though to a lesser extent. Cell–cell communication was inferred using CellChat (v1.6.1). Significance of ligand–receptor interactions was assessed by random permutation tests as implemented in the package (default settings). For Figure 4a, created in BioRender. HA, H. (2025) https://BioRender.com/hmhj4v1.

To comprehensively map the cellular heterogeneity of human dental pulp, we constructed a transcriptomic atlas using a split‐pool combinatorial barcoding scRNAseq protocol. We analyzed healthy (*n* = 4), early‐stage diseased (*n* = 4), and advanced‐stage diseased (*n* = 4) human pulp samples from molar teeth extracted for clinical reasons (Table S1, Supporting Information). Tissue samples were extracted, dissociated into single‐cell suspensions, fixed with Parse fixation kits, and stored at ‐80 °C. All samples were processed using the Parse Evercode WT v2 kit, sequenced, and analyzed with Trailmaker (**Figure** **4**a). After filtering, integrating (Harmony method), and clustering (Louvain method), we analyzed a total of 53407 cells. We identified 24 main clusters representing five systems: fibroblast, stromal, mesenchymal and odontoblastic cells (51% of the cell population), ECs (30% of the cell population), immune and blood cells (8% of the cell population), mural cells (5% of the cell population), and glial cells (5% of the cell population) (Figure 4b–d). A signature comprising *CXCL14, LUM, C1S, DCN, COL1A1, and COL1A2* characterized the fibroblasts, stromal, and mesenchymal clusters (Figure S2a, Supporting Information),^[^ ^24^, ^25^ ^]^ while *ALPL, COL1A1, NES, OMD, AXIN2, and RUNX2* were used to identify odontoblastic cell clusters (Figure S2b, Supporting Information).^[^ ^26^ ^]^ ECs were identified by canonical markers such as *CDH5*, *VWF*, *EMCN*, *ENG*, and *FLT4*,^[^ ^27^, ^28^ ^]^ while immune and blood cells expressed *FTL*, *CD68*, *MS4A7*, *MAF*, *TYROBP*, *CD74*, *PTPRC*, and *SKAP1* (Figure S2c,d, Supporting Information).^[^ ^29^ ^]^ Mural cells specifically expressed high levels of *ACTA2*, *TAGLN*, *BGN*, *NOTCH3*, *RGS5*, *PDGFRB*, and *MCAM* (Figure S2e, Supporting Information).^[^ ^25^ ^]^ Lastly, glial cells expressed *MPZ*, *MBP*, *PMP22*, *DMD*, *TRPM3*, *XKR4*, *ADGRB3*, and *GRIK2* (Figure S2f, Supporting Information).^[^ ^30^ ^]^ To characterize intercellular signaling in the healthy dental pulp microenvironment, we used CellChat (v1.6.1)^[^ ^31^ ^]^ on the integrated Seurat object containing all the cells, limited to the Healthy condition. The global communication network is shown as a chord diagram (Figure 4e), which summarizes the inferred ligand‐receptor interactions among the major cell populations. Clear communication links were observed between the fibro/stroma/mesenchymal & odontoblast cell (MSC) subsets, EC subclusters (including venules, arterioles, and capillaries), mural cells, glial cells, and immune cells, indicating that all the dental pulp cellular compartments contribute to the overall signaling network. The heatmap of interaction counts (Figure 4f) provides a quantitative overview of the number of ligand‐receptor interactions between the sender and receiver populations. This shows that MSC subsets, EC subsets, and mural cells engage in the highest number of interactions, while glial and immune populations also participate in signaling, but to a lesser extent. To complement these analyses, we examined interaction patterns in greater detail. The top 15 enriched Hallmark pathways in the Healthy condition are shown in a heatmap (Figure S3a, Supporting Information). Distinct activity profiles were observed across the different cell types: fibro/stromal/mesenchymal/odontoblast subsets displayed enrichment for pathways related to ECM organization and mesenchymal signaling, endothelial clusters showed activation of angiogenesis‐related programs, and immune subsets exhibited pathways linked to interferon responses and inflammatory signaling. By contrast, glial populations displayed relatively lower enrichment across these pathways. A grouped chord diagram summarizing communication at the population level (Figure S3b, Supporting Information) highlights broad reciprocal signaling between stromal, vascular, mural glial, and immune compartments (Figure S3c, Supporting Information). Subpopulation‐specific chord diagrams further illustrate the main patterns for each compartment: ECs interact most prominently with stromal and mural subsets and (Figure S3d, Supporting Information); stromal/MSC cells emerge as a central hub with extensive communications to all other populations (Figure S3e, Supporting Information); immune cells show more restricted interactions, primarily toward stromal and ECs (Figure S3f, Supporting Information); and mural cells engage mainly with endothelial and stromal populations (Figure S3g, Supporting Information). Together, these complementary representations establish a detailed baseline communication landscape of the healthy dental pulp, providing both global and compartment‐specific perspectives on intercellular signaling.

**Figure 5:**
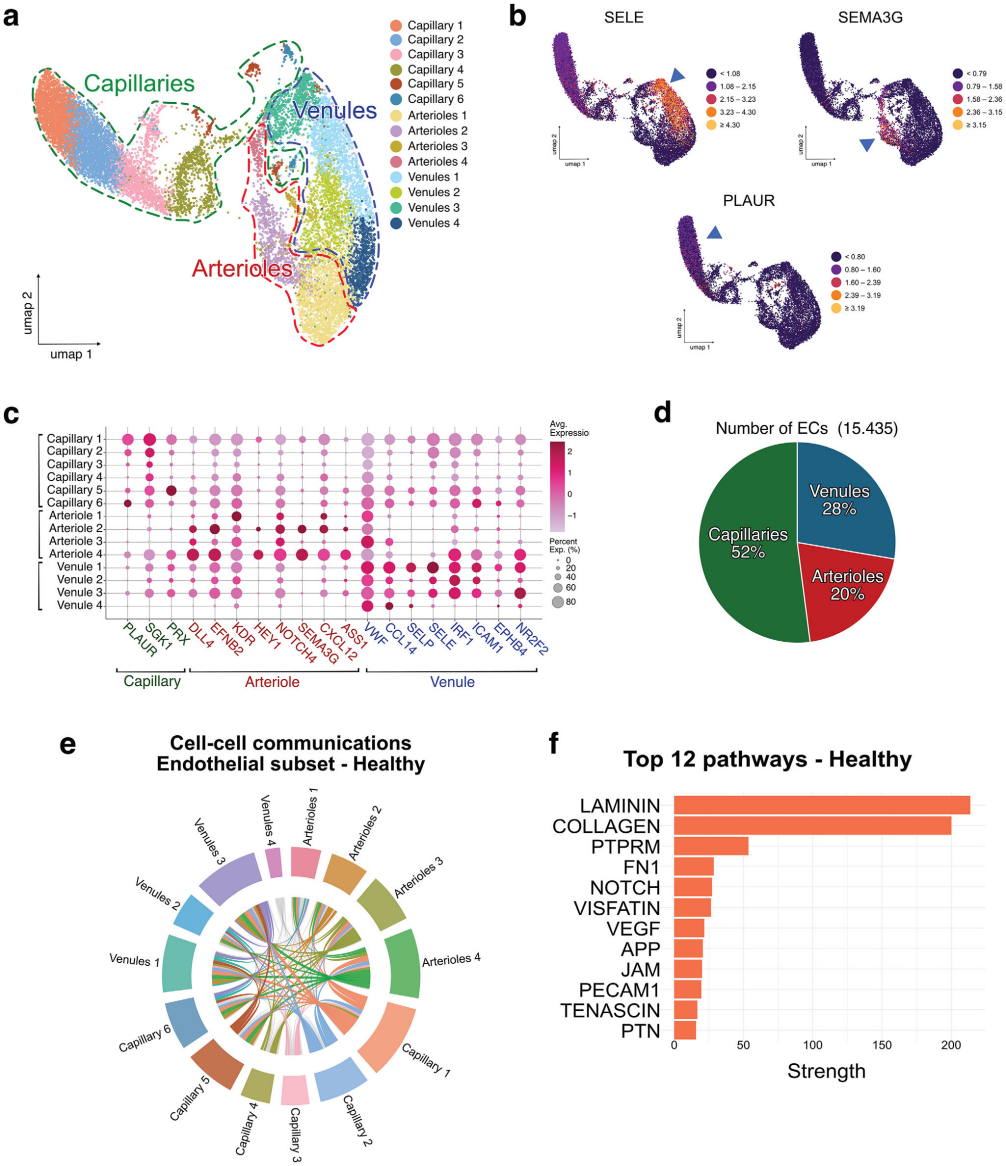
Cellular Heterogeneity of the Endothelial Population in the Human Dental Pulp. a) UMAP representation of 15435 filtered and batch corrected healthy and diseased dental pulp ECs across four healthy pulps, four early‐stage and four advanced‐stage diseased pulps. b) Relative expression of a well‐established cell type marker for each EC population projected on UMAP plots. SELE for venules; SEMA3G for arterioles; PLAUR for capillaries. c) A dot plot showing 3–8 well‐established cell type markers, allowing for the identification of each EC sub‐cluster. d) Graph showing the proportion of each of the different vascular structures. e) Chord diagram showing inferred ligand–receptor interactions among the endothelial subcluster (arterioles, venules, and capillaries) in the Healthy condition. Extensive reciprocal signaling is observed across all vascular compartments, with capillary and arteriolar clusters displaying particularly dense connectivity. Significant ligand–receptor interactions were identified by the CellChat permutation test (*p* < 0.05; min.cells = 5). Non‐significant edges are shown in grey. Sample size corresponds to the number of ECs analyzed in the Healthy condition (*n* = 7085). Edge color indicates the source subcluster. f) Bar plot of the top 12 signaling pathways enriched in endothelial‐endothelial communication. Laminin and Collagen signaling dominate, followed by PTPRM, Fibronectin (FN1), Notch, Visfatin, and VEGF pathways, together reflecting ECM organization, vascular regulation, and endothelial homeostasis. CellChat permutation test, *p* < 0.05; min.cells = 5. The sample size corresponds to the number of ECs analyzed in the Healthy condition (*n* = 7085). The radar displays aggregated pathway strength per subcluster; no between‐condition comparisons were performed.

To obtain a higher‐resolution view of EC heterogeneity, we isolated all ECs from the integrated Seurat object and generated a dedicated subset. This subset was reprocessed (normalization, dimensionality reduction, and clustering) to enable finer identification of endothelial subpopulations. We identified a total of 15.435 ECs divided into fourteen subclusters: six subclusters of capillary ECs (52% of the EC population) characterized by a higher expression of *PLAUR*, *SGK1* and *PRX*;^[^ ^27^, ^32^ ^]^ four subclusters of arteriolar ECs (20% of the ECs population) characterized with the following markers: *DLL4*, *EFNB2*, *KDR*, *HEY1*, *NOTCH4*, *SEMAG3G*, *CXCL12*, and *ASS1*;^[^ ^28^, ^32^ ^]^ and four subclusters of venous ECs (28% of the ECs population) expressing *VWF*, *CCL14*, *SELP*, *SELE*, *IRF1*, *ICAM1*, *EPHB4* and *NR2F2* (**Figure** **5**a–d; Figure S4a–c, Supporting Information).^[^ ^28^ ^]^ To further investigate the communication landscape within the endothelial compartment, we applied CellChat (v1.6.1) to this subset, restricted to the healthy condition. The resulting chord diagram illustrates the inferred ligand‐receptor interactions exclusively among the endothelial subsets. Dense reciprocal communication was observed between capillary, arterial, and venous populations, with the capillary and arteriolar clusters showing pervasive connectivity (Figure 5e). The pathway analysis identified the top signaling routes supporting the endothelial‐endothelial interactions in the Healthy state (Figure 5f). The most substantial contributions were derived from Laminin and Collagen signaling, reflecting ECM‐mediated interactions and maintenance of the ECM. Additional pathways included Fibronectin (*FN1*), *Notch*, and *VEGF*, consistent with vascular development and angiogenic signaling, as well as *PECAM1*, *JAM*, and *PTN*, associated with endothelial junction integrity and intercellular adhesion. Together, these analyses highlight that endothelial subclusters in healthy dental pulp form a highly interconnected communication network, driven by extracellular matrix signaling and vascular regulatory pathways.

Our single‐cell analyses did not reveal any distinct lymphatic subclusters within the EC subsets, and no significant expression of canonical lymphatic markers, such as *LYVE1, PROX1*, or *PDPN*, was detected (Figure S5a, Supporting Information).^[^ ^33^ ^]^ However, within the venous subclusters, the venule 1 subcluster exhibited co‐expression of *PROX1, ICAM1*, and *VCAM1*, suggesting the presence of functionally specialized reactive post‐capillary venules (REVs) (Figure S5b, Supporting Information).^[^ ^34^ ^]^

**Figure 6:**
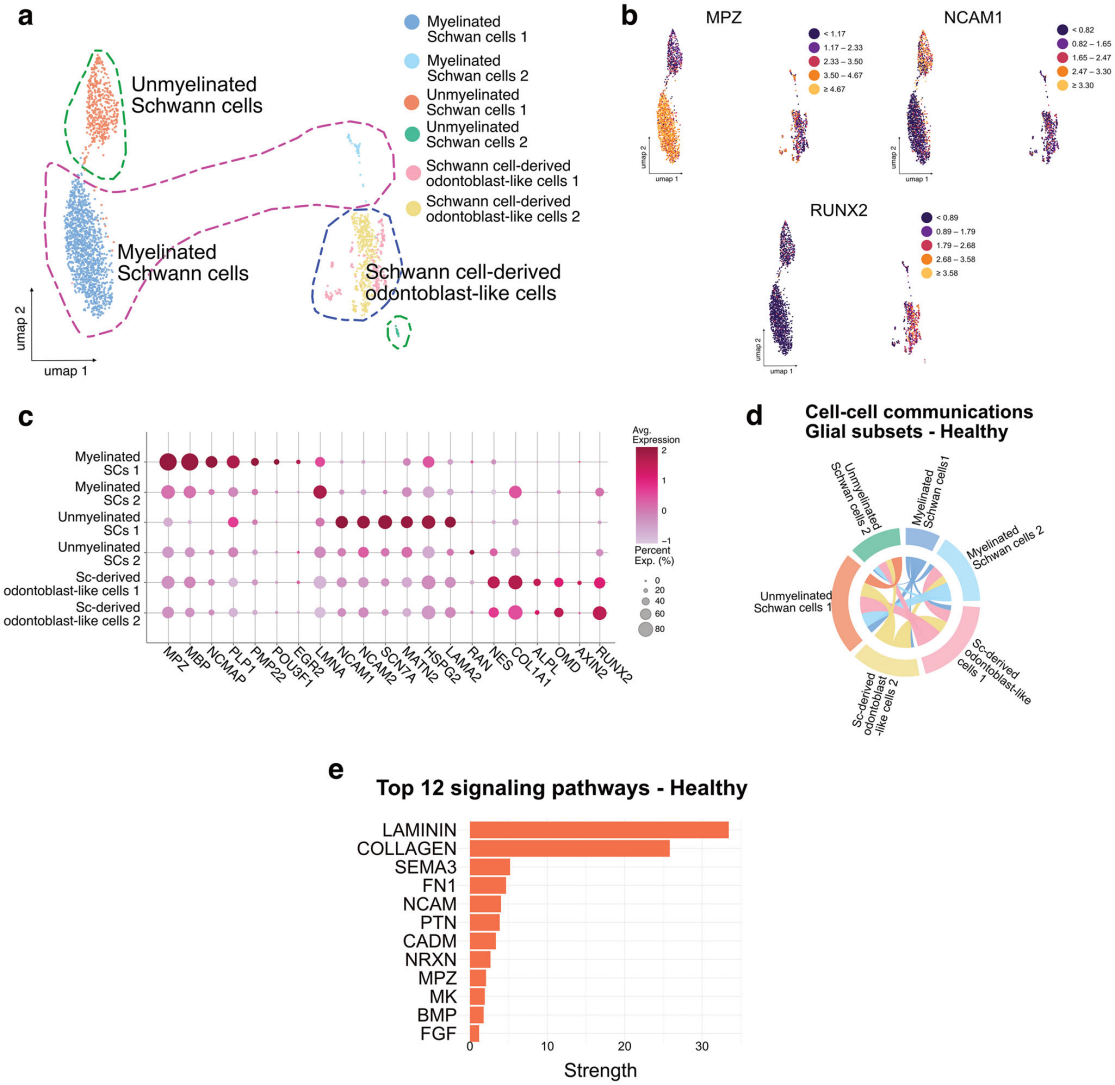
Cellular Heterogeneity of the Glial Population in the Human Dental Pulp. a) UMAP representation of 2440 filtered and batch‐corrected healthy and diseased dental pulp glial cells across four healthy pulps, four early‐stage, and four advanced‐stage diseased pulps. b) Relative expression of a well‐established cell type marker for each cell population projected on UMAP plots. MPZ for myelinated SCs; NCAM1 for unmyelinated SCs; RUNX2 for SC‐derived odontoblast‐like cells. c) A dot plot showing 21 well‐established cell type markers, enabling the identification of each cell subcluster. d) Chord diagram showing inferred ligand–receptor interactions among glial subclusters, including myelinated SCs, non‐myelinated SCs, and SC–derived odontoblast‐like cells, in the Healthy condition. Reciprocal interactions are observed between SC populations and the Sc‐derived odontoblast‐like clusters. Edges represent significant ligand–receptor interactions identified by the CellChat permutation test (*p* < 0.05; min.cells = 5). Non‐significant edges are shown in grey. Sample size corresponds to the number of glial cells analyzed in the Healthy condition (*n* = 658). Edge color indicates the source subcluster. e) Bar plot of the top 12 signaling pathways enriched in glial cell communication. Laminin and Collagen signaling predominate, followed by *SEMA3*, Fibronectin (*FN1*), *NCAM*, *PTN*, *CADM*, *NRXN*, *MPZ*, *MK*, *BMP*, and *FGF* pathways, consistent with roles in ECM interactions, axon–glia communication, and glial–mesenchymal cross‐talk. CellChat permutation test, *p* < 0.05; min.cells = 5. Sample size corresponds to the number of glial cells analyzed in the Healthy condition (*n* = 658). The radar displays aggregated pathway strength per subcluster; no between‐condition comparisons were performed.

To further investigate the glial compartment, we generated a dedicated Seurat object containing only glial cells. This analysis identified a total of 2.440 cells divided into six subclusters (**Figure** **6**a): two subclusters of myelinated Schwann cells (SCs) expressing *MPZ*, *MBP*, *NCMAP*, *PLP1*, *POU3F1*, *EGR2* and *LMNA* (Figure 6b,c; Figure S6a, Supporting Information);^[^ ^35^, ^36^ ^]^ two subclusters of non‐myelinated SCs characterized by the expression of *NCAM1*, *NCAM2*, *SCN7A*, *MATN2*, *HSPG2*, *LAMA2* and *RAN* (Figure 6b,c; Figure S6b, Supporting Information);^[^ ^35^, ^36^ ^]^ and two subclusters of SC‐derived odontoblast‐like cells characterized by the expression of *ALPL, AXIN2, NES*, *COL1A1*, *RUNX2*, and *OMD* (Figure 6b,c).^[^ ^37^, ^38^, ^39^ ^]^ These odontoblast‐like clusters were detected specifically within the glial subset and are therefore distinct from the bona fide odontoblast population identified in the integrated dataset. Their gene expression profile is consistent with previous lineage‐tracing studies showing that SC precursors can give rise to mesenchymal stem cells and odontoblasts during tooth development and repair.^[^ ^40^ ^]^ In contrast to earlier studies indicating a predominance of unmyelinated nerve fibers over myelinated ones in the dental pulp,^[^ ^41^ ^]^ our findings suggest the opposite. In a healthy pulp, myelinated and unmyelinated SCs account for 46.3% and 20.4% of the glial cell population, respectively. To explore the communication network within the glial compartment more thoroughly, we utilized CellChat (v1.6.1) on this specific subset, specifically focusing on the healthy condition. The chord diagram illustrates inferred ligand‐receptor interactions among the six identified subclusters. Reciprocal signaling was observed between myelinated and non‐myelinated SC clusters, while both also established connections with the SC‐derived odontoblast‐like populations (Figure 6d). Pathway analysis highlighted Laminin and Collagen signaling as the most prominent contributors, reflecting extracellular matrix‐mediated communication. Additional enriched pathways included *SEMA3, FN1, NVCAM, PTN, CADM, NRXXN, MPZ, MK, BMP*, and *FGF*, which are consistent with mechanisms involved in axonal guidance, glial‐neural interactions, and ECM regulation (Figure 6e). In the healthy dental pulp, the glial compartment displays marked heterogeneity and a structured interaction network, in which myelinated and non‐myelinated ECs communicate extensively and establish signaling links with SC–derived odontoblast‐like clusters.

### 2.3 Validation of Odontoblast (‐like) Subclusters Across Integrated, Fibro/Stromal, and Glial Datasets

**Figure 7:**
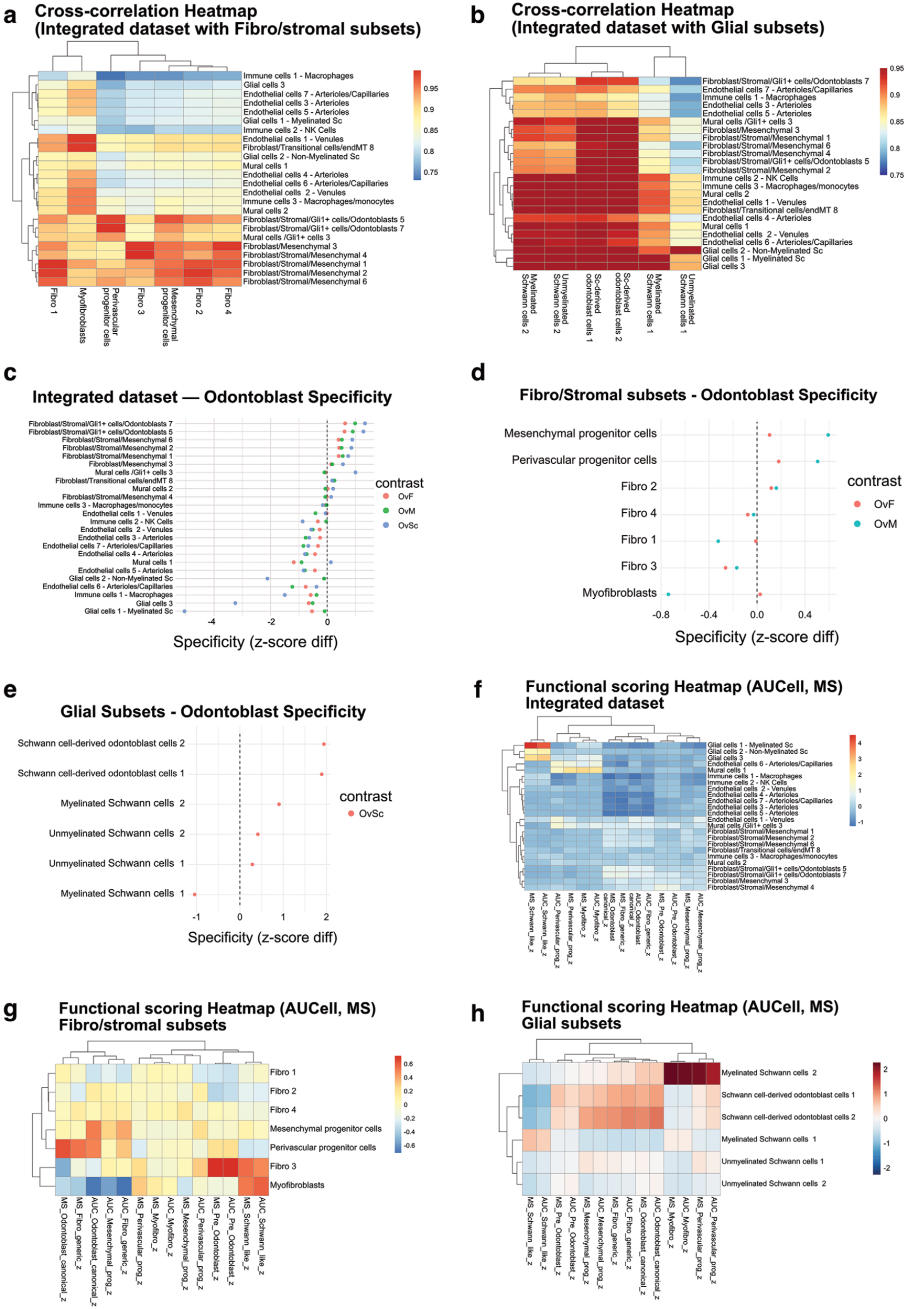
Validation of Odontoblast(‐like) Identity Across Integrated, Stromal, and Glial Subsets. a,b) Cross‐correlation heatmaps of cluster‐level expression programs in the integrated dataset a) and in the glial subset b). Heatmaps show Pearson correlation coefficients (r) between cluster‐average expression profiles from the integrated and stromal/glial objects; no inferential test was performed. Sample size per correlation equals the number of common high‐variance genes used (*n* = 1000). High correlation values (red) indicate transcriptional similarity between clusters. Odontoblast‐enriched clusters (Fibroblast/Stromal/Gli1+ cells/Odontoblasts 5 and 7) correlate strongly with fibro/stromal populations (perivascular and mesenchymal progenitor cells, and fibro 3). In the glial subset, SC‐derived odontoblast‐like clusters align with integrated odontoblast/stromal clusters, whereas myelinated and unmyelinated SCs align with glial programs. c–e) Specificity plots quantifying the relative enrichment of odontoblast programs compared to other lineages. In the integrated dataset c), odontoblast clusters show positive specificity across all three contrasts—OvF (versus fibro/stromal), OvM (versus mesenchymal progenitor), and OvSc (versus Schwann‐like)—while other lineages cluster near or below zero. Cluster‐level mean specificity scores (z‐score difference: odontoblast canonical minus comparator gene set). No inferential test was performed. Sample size for each dot corresponds to the number of cells in the cluster (range *n* = 95–6789). In the stromal subset d), odontoblast clusters display strong positive contrasts relative to fibroblasts and mesenchymal progenitors. Cluster‐level mean specificity scores (stromal progenitor/odontoblast‐related signatures). No inferential test was performed. Sample size for each dot corresponds to the number of cells in the cluster (range *n* = 1342–6615). In the glial subset e), SC‐derived odontoblast‐like clusters show high OvSc scores, clearly separating them from glial cell clusters. Cluster‐level mean specificity scores (glial‐related signatures). No inferential test was performed. Sample size for each dot corresponds to the number of cells in the cluster (range *n* = 23–1299). f–h) Functional score heatmaps showing the relative activation (AUCell/z) of predefined gene programs across clusters. In the integrated dataset f), odontoblast clusters co‐activate odontoblast and stromal programs, with endothelial and glial programs remaining low. Heatmap of mean z‐scored Module Score (MS) and AUCell (AUC) pathway scores per cluster (integrated object). No inferential test was performed. Sample size per cluster corresponds to the number of cells (range *n* = 95–6789). In the stromal subset g), gradients of odontoblast, myofibroblast, and perivascular/mesenchymal progenitor programs are evident. Heatmap of mean z‐scored Module Score (MS) and AUCell (AUC) pathway scores per cluster (stromal subset). No inferential test was performed. Sample size per cluster corresponds to the number of cells (range *n* = 1342–6615). In the glial subset h), SC‐derived odontoblast clusters acquire odontoblast/stromal signatures, whereas myelinated and unmyelinated SCs retain strong glial programs. Heatmap of mean z‐scored Module Score (MS) and AUCell (AUC) pathway scores per cluster (glial subset). No inferential test was performed. Sample size per cluster corresponds to the number of cells (range *n* = 23–1299). Contrasts: OvF = Odontoblast versus fibro/stromal programs; OvM = Odontoblast versus mesenchymal progenitor programs; OvSc = Odontoblast versus Schwann‐like programs.

We validated the odontoblast(‐like) signal across the integrated dataset and in focused fibro/stromal and glial subsets. The integrated cross‐correlation heatmap (**Figure** **7**a) shows the overall organization of cell classes, with odontoblast‐enriched fibro/stromal clusters grouped together and distinct from immune, endothelial, mural, and glial populations. The cross‐correlation map for the glial subset (Figure 7b) positions SC‐derived odontoblast‐like clusters next to odontoblast/stromal clusters, while canonical myelinated and unmyelinated SCs align with neuronal programs. The integrated specificity analysis (Figure 7c) demonstrates that odontoblast clusters score positively across the three contrasts: OvF (odontoblast versus fibro/stromal programs), OvM (odontoblast versus mesenchymal‐progenitor programs), and OvSc (odontoblast versus Schwann‐like programs), whereas non‐odontoblast lineages remain near or below zero. In the stromal subset, the specificity plot (Figure 7d) confirms positive Odontoblast‐versus‐Fibro/Mesenchymal contrasts. Contrastingly, in the glial subset, the OvSc comparison (Figure 7e) cleanly separates SC‐derived odontoblast‐like clusters from SCs. Functional scoring brings additional information: in the integrated dataset (Figure 7f), odontoblast clusters co‐activate stromal programs, while neuronal and endothelial pathways remain weak. Within the stromal compartment (Figure 7g), functional heatmaps reveal gradients spanning odontoblast, myofibroblast, and perivascular/mesenchymal progenitor states. In the glial compartment (Figure 7h), SC‐derived populations acquire odontoblast/stromal programs, whereas SCs retain their canonical identity.

### 2.4 Progressive Vascular Responses to Decay: Blood Vessels Early Outward Remodeling and Arterialization, Followed by Pathological Angiogenesis and Regression of the Vascular Network at the Dentin–Pulp Interface

**Figure 8:**
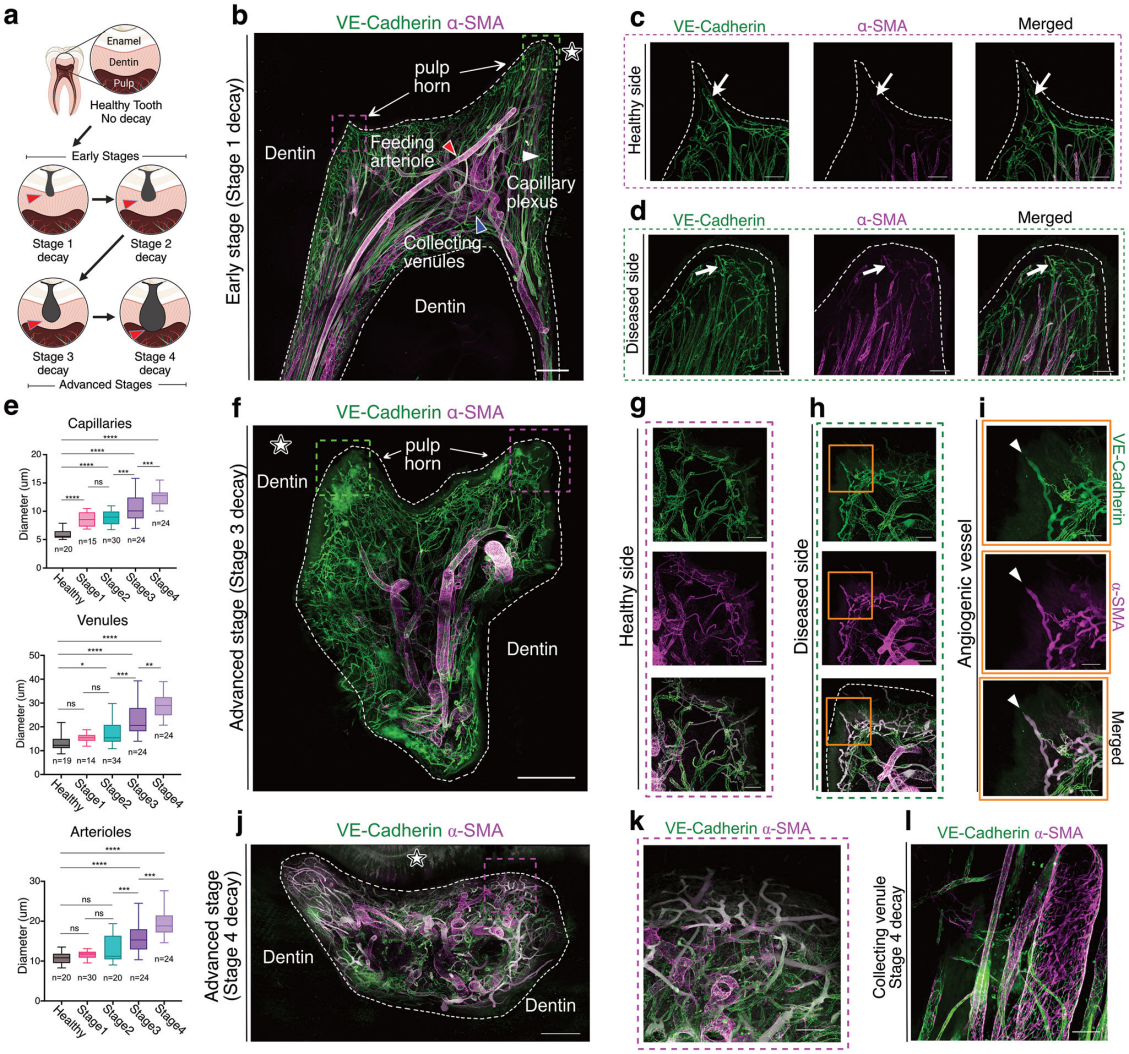
Progressive Vascular Responses to Decay: Blood Vessels Early Outward Remodeling and Arterialization Followed by Pathological Angiogenesis and Regression of the Vascular Network at the Dentin‐Pulp Interface. a) Schematic showing the different layers composing the tooth and the progression of the dental decay, from Healthy through Early to Advanced stages. b) MIP of a 486 µm z‐stack showing the vasculature of a mature dental pulp at an early stage (stage 1) of the disease. c) MIP of 317 µm z‐stack of the pulp horn on the healthy side showing the CP. (white arrow) capillary without expression of α‐SMA. d) MIP of 308 µm z‐stack of the pulp horn on the diseased side showing the CP. (white arrow) capillary with expression of α‐SMA. e) Quantification of different vessels' diameter increase (outward remodeling) on healthy dental pulps and at various stages of their disease. Boxplots show vessel diameters across stages of disease progression. n indicates the number of individual vessel measurements (≈5 per sample; 6 healthy samples and 21 diseased samples in total). Group comparisons were performed using ordinary one‐way ANOVA with post hoc multiple comparisons (GraphPad Prism). f) MIP of a 330 µm z‐stack showing the vasculature of a mature dental pulp at an advanced stage (stage 3) of the disease. g) MIP of a 217 µm z‐stack showing the pulp horn on the healthy side, showing non‐perfused vessels. h) MIP of a 338 µm z‐stack showing the pulp horn on the diseased side, showing a non‐perfused vessel sprout and potential pathological angiogenesis. i) Zoom on the vessel sprout (white arrowhead). j) MIP of a 150 µm z‐stack showing the vasculature of a mature dental pulp at an advanced stage (stage 4) of the disease. k) MIP of a 335 µm z‐stack showing non‐functional and regressed CP. l) MIP of a 125 µm z‐stack showing a heavily dilated collecting venule at stage 4 of the disease. Tissue clearing using a modified iDISCO protocol and staining for VE‐Cadherin (green) and α‐SMA (purple). Point scanning resonant confocal microscope with variable spectrum detector (Nikon AX R). Large images for (b, f, j). Plan Apo 10x λS OFN25 DIC N1 optic for (b, d, f). Plan apo Lambda 25XC Sil. Optic for (c, e, g, h, i, j, k, l). Scale bars: 500 µm for (B, F, J); 100µm for (c, d, g, h, k, l); 50 µm for (i). The white stars indicate the side of the decay. For Figure 8a, created in BioRender. HA, H. (2025) https://BioRender.com/8egv7le. For all other panels, copyright 2025, Hoang Thai HA.

Dental decay progresses slowly, with early stages (Stages 1 and 2; SiSta classification) marked by enamel demineralization and the involvement of the outer and middle thirds of the dentin. In contrast, advanced stages (Stages 3 and 4; SiSta) extend into the inner third of the dentin and pulp tissue (**Figure** **8**a). Although early decay is physically distant from the pulp, we observed that pulp cells detect damage as early as Stage 1, triggering early inflammation and vascular remodeling. At Stage 1, arterialization occurs with the recruitment of α‐SMA+ cells around capillaries in the CP on the side facing the decay in the pulp, contrasting with the absence of capillary arterialization on the healthy side (Figure 8b–d). Early vascular responses include a significant increase in capillary diameter (known as outward remodeling), which is already present at Stage 1, followed by venules at Stage 2 and arterioles at Stage 3 as the disease progresses (Figure 8e). Increased blood flow, necessary to supply nutrients to activated odontoblasts and immune cells acting as first responders, results in arterial‐like pulsatile and high‐pressure flow. This induces blood vessel differentiation, such as capillary arterialization —a process known as arteriogenesis.^[^ ^42^, ^43^ ^]^ As the disease advances, inflammation spreads within the pulp, leading to capillary regression under the decay. We observed the presence of vessel sprouting at Stage 3, where ECs start to migrate to address hypoxia by forming new vessels (angiogenesis). However, these endothelial sprouts seem to lack stable endothelial junctions and lumens, indicating a probable non‐functional, pathological state of angiogenesis that cannot resolve local hypoxia (Figure 8f–i). These require further analysis using dynamic methods, such as perfusion with a tracer, and in animal models, to provide additional evidence of disrupted cellular junctions and the absence of a lumen. Pathological angiogenesis, vessel regression, tissue degradation, and outward remodeling extend throughout the pulp as the disease progresses (Figure 8j,k,l). These vascular responses illustrate the transition from reparative (early stages) to pathological processes (late stages) within the vasculature of the dental pulp in response to tooth decay. Importantly, although we observe extensive disorganization of the vascular network at later stages, we also see remaining, although dilated, functional blood vessels under the fibrotic lesions (Figure 8k) and in the roots (Figure 8l).

**Figure 9:**
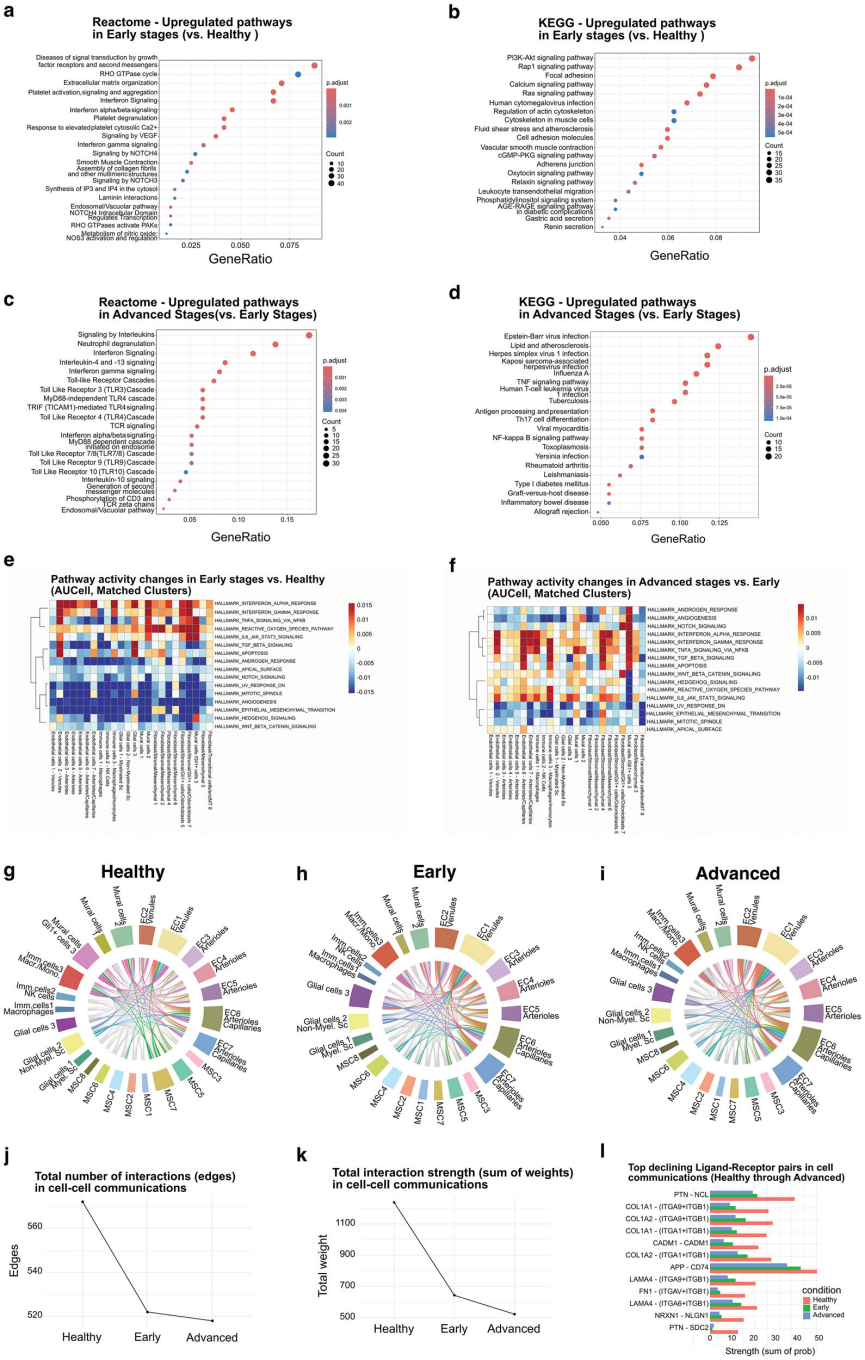
Pathway Enrichment and Cell–cell Communication Analysis Across Disease Stages. a,b) Early versus Healthy (Upregulated pathways): Reactome a) and KEGG b) highlight vascular/ECM remodeling—ECM/collagen organization, focal/adherens junctions and integrins, PI3K–Akt/Rap1/Ras, Ca^2^⁺ and cGMP–PKG signaling, vascular smooth‐muscle contraction, leukocyte transendothelial migration, and platelet activation/degranulation. Over‐representation analysis (hypergeometric test) on differentially expressed genes; *p*‐values Benjamini–Hochberg (FDR) adjusted. Sample size equals the number of mapped genes used (Up: *n* = 763; Down: *n* = 1534). c,d) Advanced versus Early (Upregulated): Reactome c) and KEGG d) show innate/adaptive inflammatory programs—Toll‐like receptor (MyD88/TRIF), NF‐κB, type I/II interferon signaling, antigen processing/presentation, T‐cell receptor signaling/differentiation, and neutrophil activation/degranulation (KEGG “infection” terms reflect these signatures rather than specific pathogens). Dot size indicates the number of overlapping upregulated genes (Count); color encodes adjusted P value (p.adjust); GeneRatio = Count/size of the upregulated list. Over‐representation analysis (hypergeometric test) on differentially expressed genes; p‐values Benjamini–Hochberg (FDR) adjusted. Sample size equals the number of mapped genes used (Up: *n* = 236; Down: *n* = 450). e,f) AUCell heatmaps of differential pathway activity across matched clusters for Early versus Healthy e) and Advanced versus Early f) stages. Scores represent mean z‐scored AUCell pathway activity per cluster. No inferential test was performed. Sample size per cluster corresponds to the number of cells (range *n* = 91–6170 for Early versus Healthy; *n* = 5–2747 for Advanced versus Early). Clusters with very few cells were retained for completeness, but should be interpreted with caution. g–i) Chord diagrams of inferred ligand–receptor interactions in Healthy g), Early h), and Advanced i) conditions, showing a progressive reduction in network connectivity. Healthy (*n* = 30 786), Early Stage (*n* = 12 573), and Advanced Stage (*n* = 10 048). Significant ligand–receptor interactions were identified by the CellChat permutation test (*p* < 0.05; min.cells = 5), while non‐significant edges are shown in grey. Edge color indicates the source cluster. j,k) Quantification of the total number of interactions j) and total interaction strength k) across stages, both declining with disease progression. Number of significant ligand–receptor interactions (edges) identified by the CellChat permutation test (*p* < 0.05; min.cells = 5) with 95% confidence intervals. Total communication strength (sum of interaction probabilities) with 95% confidence intervals. Sample size corresponds to the number of cells analyzed per condition (Healthy *n* = 30 786, Early Stage *n* = 12 573, Advanced Stage *n* = 10 048). l) Bar plot of the top declining ligand–receptor pairs (Healthy versus Advanced), highlighting the loss of ECM–associated signaling, including collagens, laminins, fibronectin, and tenascin. Bars show the summed communication probability per condition for each pair, computed from CellChat‐filtered interactions (CellChat permutation test, *p* < 0.05; min.cells = 5). No additional between‐condition test was applied at the pair level. Sample size (cells): Healthy *n* = 30 786, Early *n* = 12 573, Advanced *n* = 10 048.

To better understand the molecular basis of these changes, we performed pathway enrichment and intercellular communication analyses from the scRNAseq dataset, across disease stages. Reactome and KEGG enrichment highlighted upregulated biological pathways related to endothelial signaling and matrix organization, such as VEGF–NOS3, PI3K–Akt/Rap1/Ras, RHO‐GTPase/cytoskeleton, Ca^2^⁺ and cGMP–PKG signaling, focal/adherens junctions, vascular smooth‐muscle contraction, leukocyte transendothelial migration, platelet activation/degranulation, and ECM/collagen assembly (**Figure** **9**a,b). The same analysis, comparing advanced stages to early stages, revealed enrichment of innate and adaptive immune pathways, including Toll‐like receptor cascades (MyD88/TRIF), NF‐κB, type I/II interferon signaling, antigen processing/presentation, and T‐cell receptor signaling, as well as neutrophil activation/degranulation (Figure 9c,d). AUCell analysis further revealed a shift in pathway activity across matched clusters, with immune, stromal, and endothelial populations showing progressive activation of angiogenesis‐ and hypoxia‐related signatures in early stages, followed by metabolic and stress responses in advanced disease (Figure 9e,f). Analysis of cell–cell communication networks using CellChat showed a marked reduction in both the number of interactions and the total signaling strength of all the cellular components from healthy to advanced stages (Figure 9g–k). While the healthy pulp displayed a dense and interconnected signaling landscape (Figure 8g), this network became progressively restricted at the early stage (Figure 9h) and further diminished at advanced stages (Figure 9i). Finally, inspection of specific ligand‐receptor pairs revealed a consistent downregulation of extracellular matrix‐associated interactions, including multiple signaling pathways involving collagen, laminin, fibronectin, and tenascin (Figure 9l). These analyses indicate that vascular and stromal communication in the dental pulp undergoes an early compensation remodeling response, but progressively declines with advancing decay, leading to a loss of structural signaling pathways responsible for maintaining ECM homeostasis and reduced network connectivity.

Additional enrichment and network analyses supported these observations. In the Early versus Healthy comparison, GO enrichment highlighted terms related to endothelium development, cell‐substrate adhesion, vasculogenesis, and regulation of angiogenesis (Figure S7a, Supporting Information). The cnet plot highlighted nodes related to platelet activation, signaling, and aggregation, as well as ECM organization, endosomal/vacuolar pathways, interferon signaling, and interferon/beta signaling (Figure S7c, Supporting Information). In parallel, the protein‐protein interaction (PPI) network identified functional modules associated with blood coagulation (Module 1), vasculogenesis (Module 2), muscle cell differentiation (Module 3), endodermal cell differentiation (Module 4), microvillus organization (Module 5), and RNA splicing regulation (Module 6), together with several smaller unannotated modules (Figure S7e, Supporting Information). In the Early versus Advanced stages comparison, GO terms were associated with immune activation, leukocyte‐mediated responses, and metabolic processes (Figure S7b, Supporting Information). In contrast, Reactome networks highlighted categories linked to signaling by interleukins, interleukin‐4 and ‐7 signaling, neutrophil degranulation, interferon gamma signaling, and interferon signaling, indicating inflammatory activation in advanced stages (Figure S7d, Supporting Information), and a switch in the metabolic state of the cells. The PPI network clustered upregulated genes into modules associated with antigen processing and presentation (Module 1), regulation of inflammatory response (Module 2), microglial cell activation (Module 3), and L‐leucine transport (Module 4), alongside several unannotated smaller clusters (Figure S7f, Supporting Information). These complementary GO, pathway, and PPI analyses provide additional evidence for the progressive shift from vascular/structural remodeling in early pulp responses to immune‐ and inflammation‐related remodeling in advanced stages.

**Figure 10:**
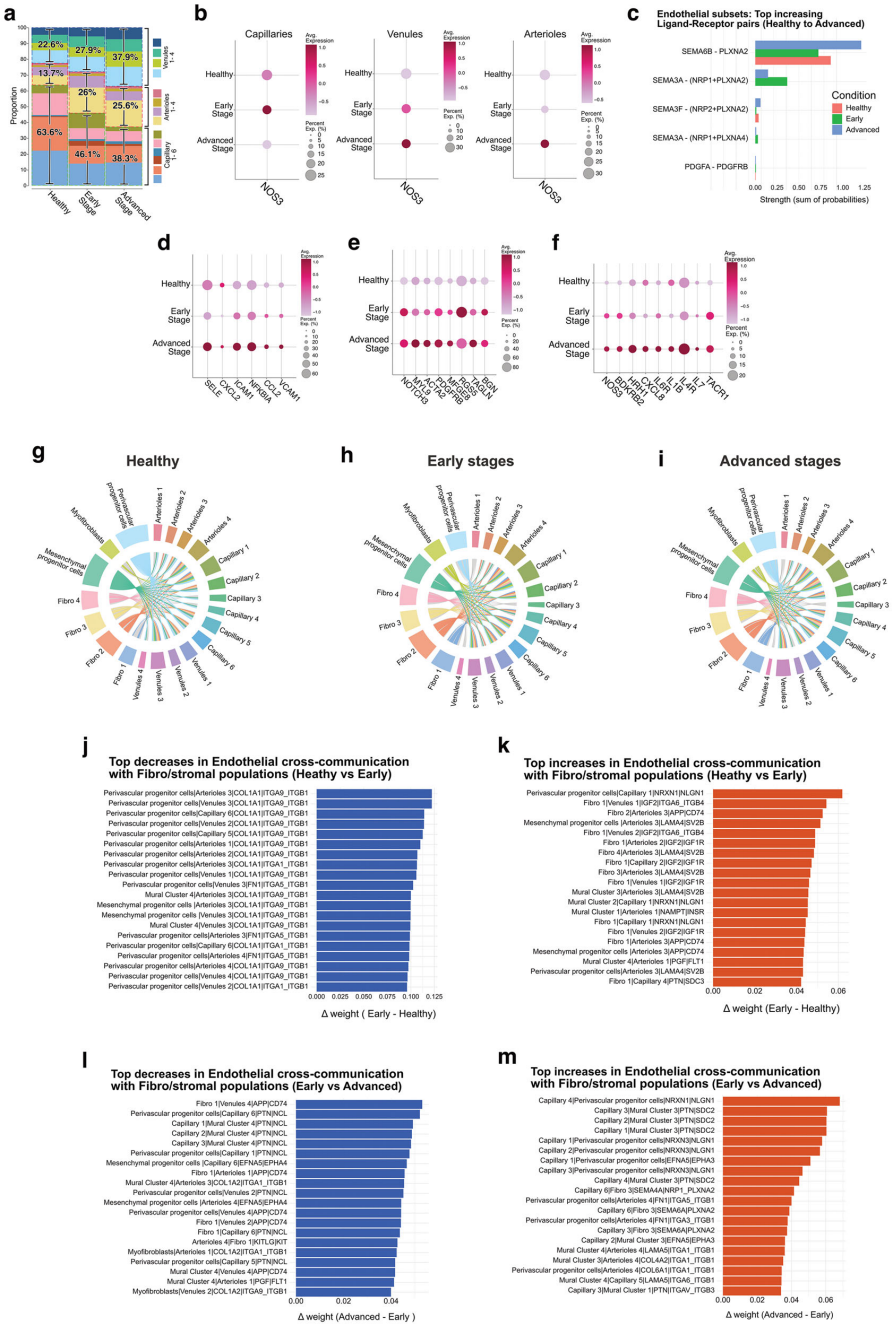
Endothelial Subcluster Dynamics and Fibroblast–Endothelial Crosstalk During Progression of Decay. a) Proportions of endothelial subclusters (capillaries, venules, and arterioles) across Healthy, Early, and Advanced stages, showing a progressive increase in venular and arteriolar populations. Metabolically stress–related genes such as *NDRG1, TPI1, LDHA, SLC20A1, ADM, TUBB6*, and *MIF* were consistently upregulated, while *AK3* and *ALDOA* did not show a clear increase across stages. b) Progressive upregulation of *NOS3* expression in capillary, venular, and arteriolar clusters. c) Endothelial subset analysis. Top increasing ligand–receptor interactions from healthy to advanced pulp. SEMA3A–NRP1/PLXNA2 signaling peaks at the early stage, suggesting transient vascular remodeling, whereas SEMA6B–PLXNA2 and PDGFA–PDGFRB progressively rise, consistent with sustained angiogenic and fibrotic responses. Bars show the summed communication probability per condition for each pair, computed from CellChat‐filtered interactions (CellChat permutation test, *p* < 0.05; min.cells = 5). No additional between‐condition test was applied at the pair level. Sample size (cells): Healthy *n* = 7085, Early *n* = 4711, Advanced *n* = 3639. d–f) Expression of endothelial activation markers (*SELE, CXCL2, ICAM1, NFKBIA, CCL2, VCAM*), mural arterialization markers (*NOTCH3, MYL9, ACTA2, PDGFRB, MFGE8, RGS5, TAGLN, BGN*), and inflammatory mediators (*BDKRB2, HRH1, CXCL8, IL6R, IL1B, IL4R, IL7, TACR1*) across stages. (g‐i) Chord diagrams of fibroblast‐to‐endothelial communication in healthy g), early h), and advanced i) pulps. Healthy pulps are characterized by fibroblast‐derived collagen–integrin adhesion, whereas early stages exhibit diverse fibroblast outputs, including IGF2, laminin, and APP–CD74. In advanced disease, fibroblast signaling is markedly reduced, with endothelial inputs becoming more prominent. Healthy (*n* = 7085), Early Stage (*n* = 4711), and Advanced Stage (*n* = 3639). Significant ligand–receptor interactions were identified by the CellChat permutation test (*p* < 0.05; min.cells = 5), while non‐significant edges are shown in grey. Edge color indicates the source subcluster. j,k) Barplots of top decreases j) and increases k) ligand–receptor interactions between healthy and early stages, showing the loss of collagen–integrin adhesion and the gain of growth and remodeling pathways. Bars show the summed communication probability per condition for each pair, ranked by Δ (Early − Healthy): decreases (j, Δ < 0) and increases (k, Δ > 0). Interactions are retained if significant by the CellChat permutation test (*p* < 0.05; min.cells = 5). No additional between‐condition test was applied at the pair level. Sample size (cells, endothelial subset): Healthy *n* = 7085, Early *n* = 4711. l,m) Barplots of top decreases l) and increases m) interactions between early and advanced stages, showing loss of pleiotrophin (PTN–NCL) and ephrin–Eph (EFNA5–EPHA4) interactions, and a relative increase in endothelial‐to‐stromal NRXN–NLGN and SEMA–PLXNA signaling in Early versus Advanced stages. Bars show the summed communication probability per condition for each pair, ranked by Δ (Advanced − Early): decreases (l, Δ < 0) and increases (m, Δ > 0). Same criteria and display. Sample size (cells, endothelial subset): Early *n* = 4711, Advanced *n* = 3639.

The distribution of endothelial subclusters changed notably during disease progression (**Figure** **10**a). In the healthy pulp, capillary ECs were predominant, whereas the relative proportion of venular and arteriolar clusters increased in both the early and advanced stages. This cellular reorganization was accompanied by transcriptional changes in genes related to metabolic stress and vascular function. Among stress‐associated genes, *NDRG1, TPI1, LDHA, SLC20A1, ADM, TUBB6, and MIF* were consistently upregulated, while *AK3* and *ALDOA* did not show an apparent increase across stages.^[^ ^44^, ^45^, ^46^ ^]^ *NOS3*, a marker of vasodilation,^[^ ^47^, ^48^ ^]^ showed progressive upregulation across capillaries, venules, and arterioles (Figure 10b). In the endothelial subsets, semaphorin signaling (SEMA3A–NRP1/PLXNA2) peaks early, suggesting a role in initial vascular remodeling by preventing sprouting.^[^ ^49^ ^]^ In contrast, SEMA6B–PLXNA2 and PDGFA–PDGFRB progressively increase, consistent with sustained angiogenic^[^ ^50^ ^]^ and fibrotic responses during disease progression (Figure 10c). In parallel, markers of endothelial activation, including *SELE, CXCL2, ICAM1, NFKBIA, CCL2, and VCAM*,^[^ ^51^ ^]^ mural arterialization markers such as *NOTCH3*, *MYL9*, *ACTA2*, *PDGFRB*, *MFGE8*, *RGS5*, *TAGLN*, and *BGN*,^[^ ^52^, ^53^, ^54^ ^]^ and inflammatory mediators including *BDKRB2*, *HRH1*, *CXCL8*, *IL6R*, *IL1B*, *IL4R*, *IL7*, and *TACR1* displayed increased expression with stage‐ and population‐specific variation (Figure 10d–f). BDKRB2 encodes the bradykinin receptor B2, a G‐protein‐coupled receptor mediating vasodilation and inflammation,^[^ ^55^ ^]^ *HRH1* encodes the histamine H1 receptor, *CXCL8* (IL‐8) acts as a potent angiogenic chemokine via VEGF activation,^[^ ^56^, ^57^ ^]^ and *TACR1* (substance P receptor, NK1R) functions as a vasodilatory neuropeptide receptor.^[^ ^58^ ^]^ Together, these data indicate that vascular remodeling during decay progression is characterized by a shift in endothelial subcluster proportions, the selective induction of metabolic stress–related genes, the upregulation of vasodilatory and inflammatory mediators, and the involvement of particular ligand–receptor interactions that facilitate vascular activation.

Analysis of fibroblast‐to‐endothelial communication revealed progressive remodeling during the progression of the disease.^[^ ^59^, ^60^ ^]^ In healthy pulps, chord diagrams revealed fibroblast‐derived signals as the dominant ones (Figure 10g). At early stages, the overall pattern shifted, with a visible diversification of fibroblast outputs (Figure 9h). In advanced disease, the chord diagram alone still displayed multiple connections (Figure 10i); however, cross‐checking with the differential edge analysis revealed that fibroblast‐derived inputs were reduced, while endothelial‐derived signals became more prominent. Barplots provided molecular detail to these transitions: between healthy and early stages, the strongest decreases involved collagen–integrin interactions, which usually ensure stromal–vascular adhesion, while increases were driven by *IGF2*, laminin, and *APP‐CD74*, pathways associated with growth, ECM remodeling, and inflammatory signaling (Figure 10j,k). Between early and advanced stages, the most prominent decreases included pleiotrophin (*PTN‐NCL*) and ephrin–Eph (*EFNA5‐EPHA4*) interactions, both implicated in angiogenesis and repair, whereas top increased edges highlighted atypical endothelial‐derived signals such as *NRXN‐NLGN* and semaphorin–plexin (*SEMA4‐NRP1/PLXNA2*, and *SEMA6A*‐*PLXNA2*), interactions more commonly linked to neuronal guidance and maladaptive tissue remodeling (Figure 10l,m). These results demonstrate a transition from fibroblast‐driven adhesion (healthy) to balanced pro‐regenerative crosstalk (early stages) and, finally, to endothelial‐driven maladaptive signaling (advanced stages).

### 2.5 Neurogenesis Develops at the Dentin–Pulp Interface in Early Stages of Decay, Followed by Neural Regression and Disorganization at Later Stages

**Figure 11:**
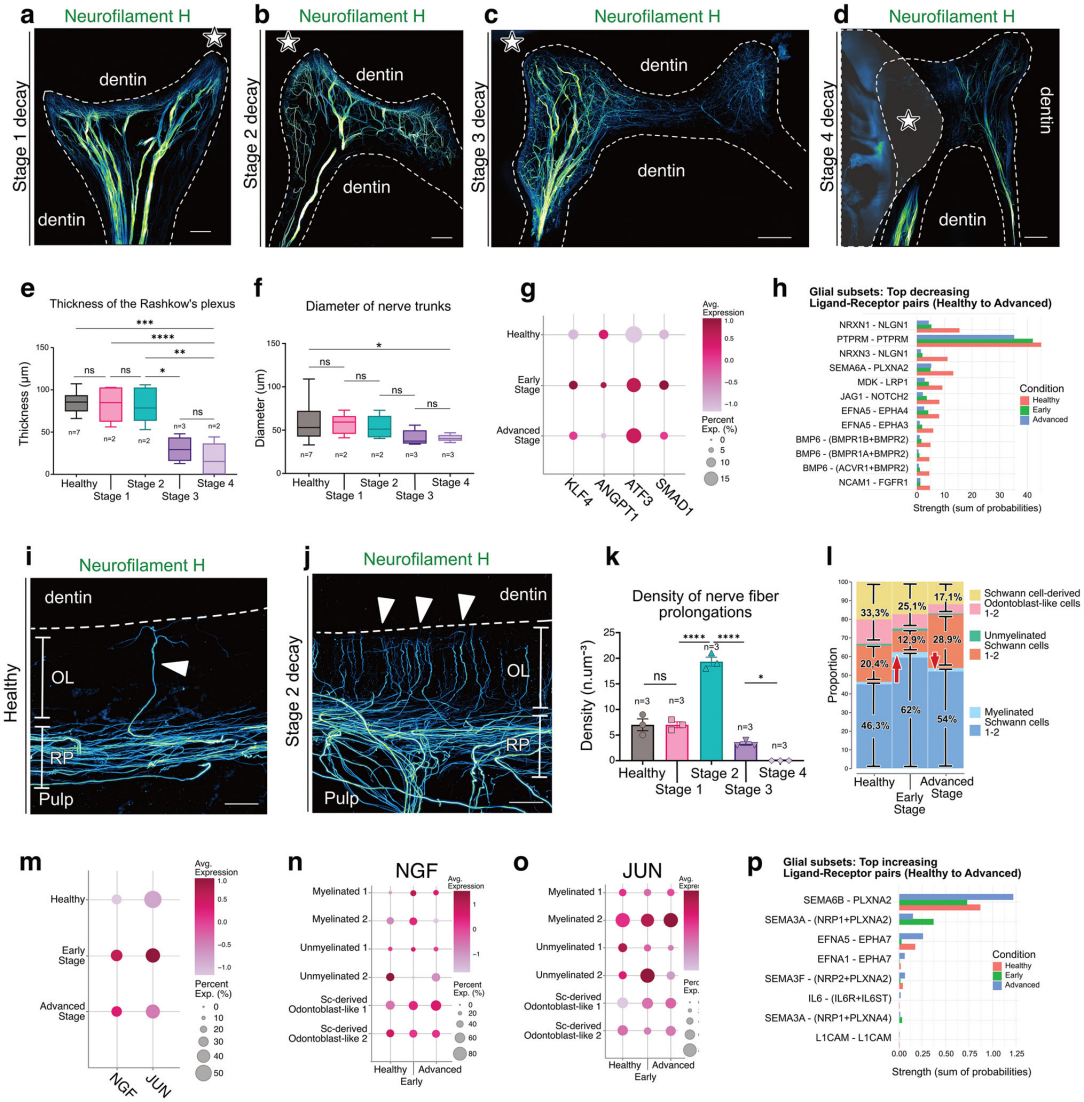
Neurogenesis Develops at the Dentin‐Pulp Interface in the Early Stages of the Disease, Followed by Neural Regression at Later Stages. a–d) MIP of z‐stack showing the neural network of a mature dental pulp at various stages of the disease. (white star) side where lays the decay. a) stage 1 (233 µm z‐stack), b) stage 2 (284 µm z‐stack), c) stage 3 (293 µm z‐stack), and d) stage 4 (291 µm z‐stack) of the disease. The white stars indicate the side of the decay. e) Graph showing the thickness of the RP at different health states. ***n*** indicates the number of biological samples (Healthy: 7, Stage 1: 2, Stage 2: 2, Stage 3: 3, Stage 4: 2). For each sample, 1–3 measurements were averaged to obtain a single value. Statistical comparisons were performed using a mixed‐effects model (REML) with Tukey's multiple comparisons (GraphPad Prism). f) Graph showing the diameters of nerve trunks at different health states. ***n*** indicates the number of biological samples (Healthy: 7, Stage 1: 2, Stage 2: 2, Stage 3: 3, Stage 4: 3). For each sample, multiple measurements were averaged to obtain a single value. Statistical comparisons were performed using a mixed‐effects model (REML) with Tukey's multiple comparisons (GraphPad Prism). g) Dot plot showing stage‐specific expression of neurogenic and inflammatory mediators (*KLF4, ANGPT1, ATF3, SMAD1*), which increase during early disease, consistent with pulp inflammation and neuronal stress responses. Bars show the summed communication probability per condition for each pair, computed from CellChat‐filtered interactions (CellChat permutation test, *p* < 0.05; min.cells = 5). No additional between‐condition test was applied at the pair level. Sample size (cells, glial subset): Healthy *n* = 658, Early *n* = 1055, Advanced *n* = 727. h) Bar plot of the top decreasing ligand–receptor pairs (Healthy → Advanced), illustrating the loss of multiple neurotrophic and axon guidance interactions, including *NRG1–ERBB4* and *BMP5–BMPR2*. i) MIP of a 140 µm z‐stacks in a healthy dental pulp. j) MIP of a 205 µm z‐stacks in a diseased dental pulp at stage 2. (RP) Rashkow's plexus; (OL) the odontoblastic layer. k) Graph showing the density of nerve fibers prolongations (in n.µm‐3) at different health states. Three independent samples were analyzed per group. Comparisons between groups were performed using repeated‐measures one‐way ANOVA, followed by Tukey's multiple comparisons test (GraphPad Prism). l) proportions of different sub‐clusters at different health states. m) Dot plot showing two well‐established markers associated with axon growth. n) Dot plot showing the relative expression of *NGF* at different health states. o) Dot plot showing the relative expression of *JUN* at different health states. p) Bar plot of the top increasing ligand–receptor pairs (Healthy → Advanced), highlighting enhanced semaphorin signaling (*SEMA3A–NRP1/PLXNA2*, *SEMA4B–PLXNA2*) and *PDGFA–PDGFRA*, reflecting altered glial communication during pulp disease progression. Bars show the summed communication probability per condition for each pair, computed from CellChat‐filtered interactions (CellChat permutation test, *p* < 0.05; min.cells = 5). No additional between‐condition test was applied at the pair level. Sample size (cells, glial subset): Healthy *n* = 658, Early *n* = 1055, Advanced *n* = 727. a–d,i,j) Tissue clearing using a modified iDISCO protocol and staining with Neurofilament F (green) for the neural network. Point scanning resonant confocal microscope. Large images for (a–d). Plan Apo 10x λS OFN25 DIC N1 optic for (a–d). Plan apo Lambda 25XC Sil. Optic for (i,j). Scale bars: 500 µm for (a–d), 50 µm for (i, j). Copyright 2025, Hoang Thai HA.

Progression of dental decay revealed increasing spatial disorganization of nerve fibers, with a progressive reduction in the diameter of nerve trunks and thinning of the RP (**Figure** **11**a–f). In healthy pulps, the RP lies beneath the OL, with nerve fibers running parallel to the dentin surface and a limited number of fibers extending through the OL into dentinal tubules alongside odontoblastic processes (Figure 11i). At Stage 2, there is a notable increase in nerve fiber density crossing the OL (Figure 11j,k), which may underlie heightened tooth sensitivity reported at this stage.^[^ ^61^, ^62^, ^63^ ^]^ Analysis of endothelial–nerve signaling showed upregulation of inflammatory and axon guidance–associated genes including *KLF4, ANGPT1, ATF3, and SMAD1* (Figure 11g), consistent with early pulp inflammation and neurogenic responses.^[^ ^64^, ^65^, ^66^, ^67^ ^]^ In parallel, ligand–receptor analysis revealed a marked decline in neurotrophic interactions across stages (Figure 11h), whereas other signaling pairs, notably semaphorin (*SEMA3A–NRP1/PLXNA2* and *SEMA4B–PLXNA2*), showed an increase from Healthy to Advanced pulp (Figure 11p), indicating a potential repulsion of nerve growth or even neural fiber apoptosis.^[^ ^68^ ^]^ At the cellular level, proportions of neural subclusters shifted with disease progression (Figure 11l). Myelinated SCs predominated in the healthy pulp, whereas advanced disease was characterized by a reduction in both myelinated and unmyelinated SCs, as well as a relative increase in SC–derived odontoblast‐like cells (1 and 2). These clusters, defined by co‐expression of glial and odontoblastic markers, are transcriptionally distinct from bona fide odontoblasts of mesenchymal origin. Their expansion across disease stages suggests an adaptive glial‐derived response contributing to pulp remodeling. Expression analysis confirmed that axon growth–associated genes, such as *NGF* and *JUN*, were upregulated not only in myelinated SCs but also in the SC–derived odontoblast‐like subclusters during the early disease stages (Figure 11m,o).^[^ ^69^, ^70^ ^]^

### 2.6 Early Immune Response Followed by Macrophage Recruitment and Activation as the Disease Progresses

**Figure 12:**
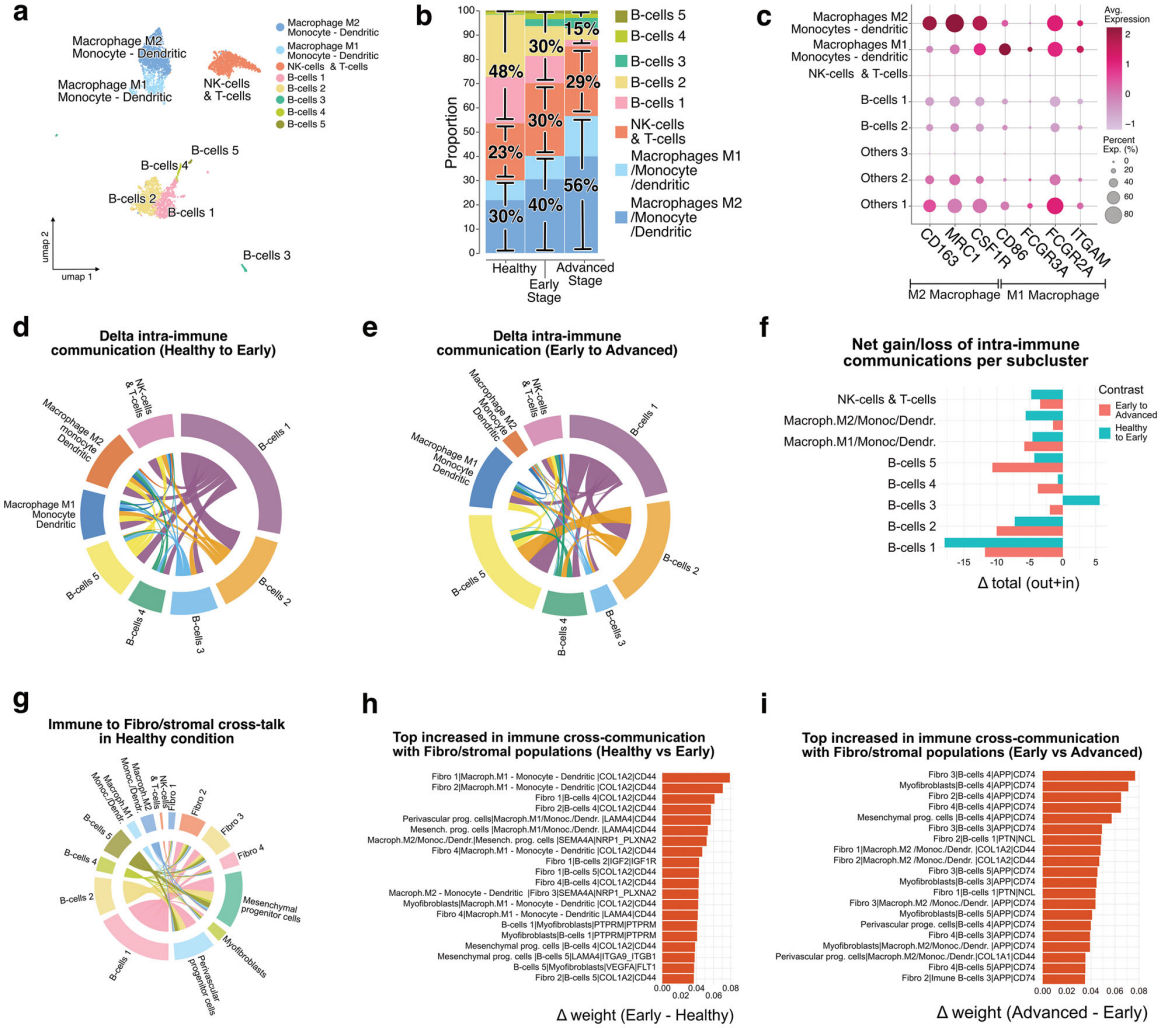
Progressive Reorganization of Immune Populations and Fibroblast Crosstalk. a) Immune subset: UMAP representation of 4.349 filtered and batch corrected healthy and diseased dental pulp cells across four healthy pulps, four early‐stage and four advanced‐stage diseased pulps. b) Immune subset: Proportions of different immune cell sub‐clusters according to their health states (healthy, early, and advanced stage disease). c) Immune subset: A dot‐plot defines major immune populations, including macrophage M1/M2, NK/T cells, and B‐cell subsets 1–5. d,e) Immune subset analysis. Δ‐Chord diagrams showing net changes in intra‐immune communication plotted in BOTH modes, comparing Healthy to Early d) and Early to Advanced e) stages. They show a shift from macrophage‐centered interactions to increased B‐cell contributions. Δ‐Chord diagrams of intra‐immune communication. Edges represent changes in ligand–receptor interaction probability between conditions, with the dominant direction of change indicated by edge color (source subcluster). Significant interactions were identified by the CellChat permutation test (*p* < 0.05; min.cells = 5), whereas non‐significant changes are shown in grey. Sample size (cells, immune subset): Healthy *n* = 2739; Early *n* = 624; Advanced *n* = 986. f) Immune subset analysis. Change in total interaction weight per immune population across contrasts, confirming a global decrease in macrophage signaling and a progressive increase in B‐cell activity. Bars indicate the difference between the number of significant outgoing and incoming interactions gained versus lost across conditions, computed from CellChat interaction probabilities (permutation test, *p* < 0.05; min.cells = 5). Positive values indicate a net gain of communications, negative values a net loss. Sample size (immune subset): Healthy *n* = 2739; Early *n* = 624; Advanced *n* = 986. g) Immune–Fibro subset comparison. Chord diagram of Immune to Fibro/stromal communications showing fibroblast subsets as major immune targets, with edges colored by immune sender population. Significant interactions were defined by the CellChat permutation test (*p* < 0.05; min.cells = 5), while non‐significant edges are shown in grey. Sample size (cells): immune subset Healthy *n* = 2739, Early *n* = 624, Advanced *n* = 986; stromal subset Healthy *n* = 7085, Early *n* = 4711, Advanced *n* = 3639. h) Immune‐Fibro subset comparison. Barplot showing the top increased Immune and Fibro/stromal ligand–receptor edges in Healthy to Early, highlighting macrophage‐to‐fibroblast communication through *COL1A2–CD44*, *APP–CD74*, and *SEMA4A–NRP1/PLXNA2*. i) Immune–Fibro subset comparison. Barplot showing the top increased Immune and Fibro/stromal ligand–receptor edges in Early to Advanced, showing reinforcement of B‐cell (notably B‐cell 3) to fibroblast interactions via *APP–CD74* and *PTN–NCL*. Bars show the summed communication probability per condition for each pair, ranked by Δ. Interactions were retained if significant by the CellChat permutation test (*p* < 0.05; min.cells = 5). No additional between‐condition test was applied at the pair level. Sample sizes: immune subset Healthy *n* = 2739, Early *n* = 624, Advanced *n* = 986; stromal subset Healthy *n* = 7085, Early *n* = 4711, Advanced *n* = 3639.

Single‐cell analysis identified diverse clusters of blood and immune cells labeled with gene markers: *EBF1* for B‐cells, *HLA*‐*DRA* for dendritic cells,^[^ ^71^ ^]^ *CD14* for monocytes,^[^ ^72^ ^]^ *CD68* for macrophages,^[^ ^73^ ^]^ *PARP8* for NK cells,^[^ ^74^ ^]^ and *CD3E* for T‐cells^[^ ^75^ ^]^ (**Figure** **12**a–c). The major immune populations consisted of macrophages/monocytes/dendritic cells, B‐cells, and NK/T‐cells, with dynamic changes across disease stages (Figure 12b). Macrophages were further classified into M2 (*CD163, MRC1, CSF1R*) and M1 (*CD86, FCGR3A, ITGAM*) subtypes (Figure 12c).^[^ ^76^ ^]^

Chord diagrams summarizing immune communication between conditions revealed a progressive reorganization of the signaling. From Healthy to Early stages of decay (Figure 12d), macrophages remained central players, but their contribution declined, while B‐cell subsets increased their outgoing signals. From Early to Advanced (Figure 10e), B‐cells, particularly the B3–B5 subclusters, expanded their communications, reshaping the overall immune network. A quantitative comparison of total interaction weight per node confirmed these stage‐specific shifts (Figure 12f), showing a global decline in macrophage involvement and an increase in B‐cell 3 subcluster activity from the Healthy to the Early stages.

Zooming in on immune–fibroblast interactions highlighted the nature of this transition. At early stages, macrophages, including the M2 subtype, established strong connections with fibroblasts through ligand–receptor pairs such as *COL1A2–CD44*, *APP–CD74*, and *SEMA4A–NRP1/PLXNA2* (Figure 12h).^[^ ^77^ ^]^ These axes highlight the role of macrophages in initiating stromal remodeling. At later stages, however, B‐cells, especially B‐cells 3, emerged as dominant fibroblast partners (Figure 12f), signaling through APP–CD74 and PTN–NCL to reinforce fibroblast activation and stromal remodeling. This B‐cell 3 subcluster has been characterized (Figure S8, Supporting Information).

Together, these findings suggest that immune responses in the dental pulp are progressively reconfigured, with an initial macrophage–fibroblast dialogue that is subsequently complemented and reinforced by B‐cell–fibroblast interactions, thereby sustaining stromal remodeling and fueling fibrosis.^[^ ^78^, ^79^, ^80^ ^]^

### 2.7 Perivascular Progenitor Cell Differentiation into Fibroblasts at the Initiation of the Disease is Followed by Myofibroblasts Activation and Reactive Fibrosis in Advanced Disease Stages

**Figure 13:**
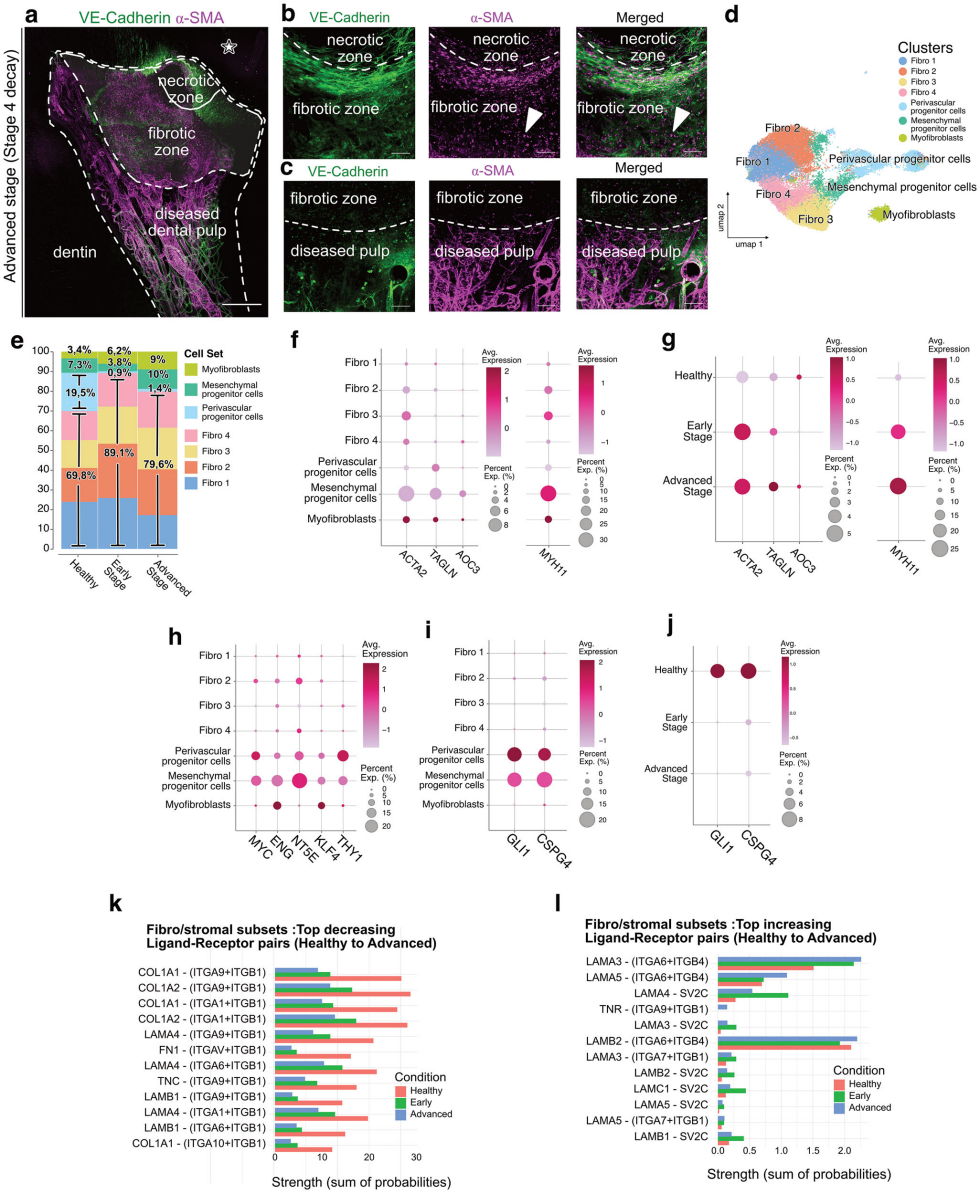
Perivascular Progenitor Cell Differentiation into Fibroblasts at the Initiation of the Disease is Followed by Myofibroblast Activation and Reactive Fibrosis in Advanced Disease Stages. a) MIP of a 152 µm z‐stack showing the presence of necrotic and fibrotic zones in a mature dental pulp at an advanced stage (stage 4) of the disease. The white star indicates the side of the decay. b) MIP of 181 µm z‐stack showing the transition between the necrotic and fibrotic zones. c) MIP of 122 µm z‐stack showing the transition between the fibrotic zone and the adjacent diseased dental pulp. d) UMAP representation of 28.419 filtered and batch corrected healthy and diseased dental pulp fibroblast and stromal cells across 4 healthy pulps, 4 early stage and 4 advanced stage diseased pulps. e) Proportions of different fibroblast and stromal cell sub‐clusters according to their health states (healthy, early‐stage, and advanced‐stage disease). f,g) Dot plot showing 4 well‐established markers associated f) with myofibroblasts. h) with myofibroblasts at various health states. h) Dot plot showing 5 well‐established markers associated with progenitor stem cells. i,j) Dot plot showing 2 well‐established markers associated i) with perivascular progenitor cells and j) with perivascular progenitor cells at various health states. k) Top decreasing ligand–receptor interactions (Healthy to Advanced), highlighting reduced collagen–integrin and fibronectin–integrin signaling. l) Top increasing ligand–receptor interactions (Healthy to Advanced), showing upregulation of laminin–integrin and laminin–SV2C signaling. Bars represent the summed communication probability per condition for each pair, computed from CellChat‐filtered interactions (CellChat permutation test, *p* < 0.05; min.cells = 5). No additional between‐condition test was applied at the pair level. Sample size (cells, fibro/stromal subset): Healthy *n* = 7085; Early *n* = 4711; Advanced *n* = 3639. Tissue clearing using a modified iDISCO protocol and staining for VE‐Cadherin (green) and α‐SMA (purple). Point scanning resonant confocal microscope. Large images for (a). Plan apo Lambda 25XC Sil. Optic for (a–c). Scale bars: 500 µm for (a); 100 µm for (b,c). Copyright 2025, Hoang Thai HA.

In advanced stages of tooth decay, extensive tissue degradation is observed, with a necrotic zone directly beneath the decay site. Adjacent to this necrotic zone, we observe a fibrotic zone without any vascular and nervous structures but abundant α‐SMA‐expressing myofibroblasts (**Figure** **13**a–c). Myofibroblast subclusters were identified using gene markers such as *ACTA2, TAGLN, AOC3, and MYH11* (Figure 13d–f)^[^ ^81^, ^82^ ^]^ with their relative expression levels significantly increasing as the disease progresses (Figure 13g). Additional subclusters were identified: fibroblasts (*VIM, CXCL14, LUM, C1S, DCN, FBLN2, POSTN, COL1A2, and CDH11*),^[^ ^83^ ^]^ perivascular progenitor cells (*GLI1, CSPG4*)^[^ ^84^, ^85^ ^]^ (Figure 13d,i,j), and mesenchymal progenitor cells (*MYC, ENG, NT5E, KLF4, and THY1*)^[^ ^86^ ^]^ (Figure 13d,h). In healthy tissue, perivascular progenitor cells are prevalent; however, this cellular population disappears at the onset of tooth decay (Figure 13e,i,j). This indicates the early differentiation of Gli1^+^ perivascular progenitor cells into fibroblasts (subclusters 2, 3, and 4), whose numbers increase significantly during disease progression. This differentiation could be triggered by the early activation of the capillary network as part of the reparative response. Subsequently, these fibroblasts further differentiate into myofibroblasts under activation by M2 macrophages as the disease progresses.^[^ ^77^ ^]^ Ligand–receptor analysis further revealed a shift in ECM communication during fibrosis. Collagen‐ and fibronectin‐integrin interactions showed a marked decrease with disease progression (Figure 13k), while laminin‐based interactions (*LAMA3, LAMA5, LAMB2*) increased, particularly through integrins and SV2C (Figure 13l). This transition suggests a remodeling of the ECM, from collagen‐rich to laminin‐dominant niches, consistent with the establishment of a fibrotic microenvironment in advanced stages. Of interest, this process might indicate the formation of “laminin scaring”, a laminin‐dense fibrotic scar which has been shown to impede the regenerative potential of muscle progenitor cells.^[^ ^87^ ^]^

### 2.8 Odontoblasts’ Fate With the Progression of the Disease

**Figure 14:**
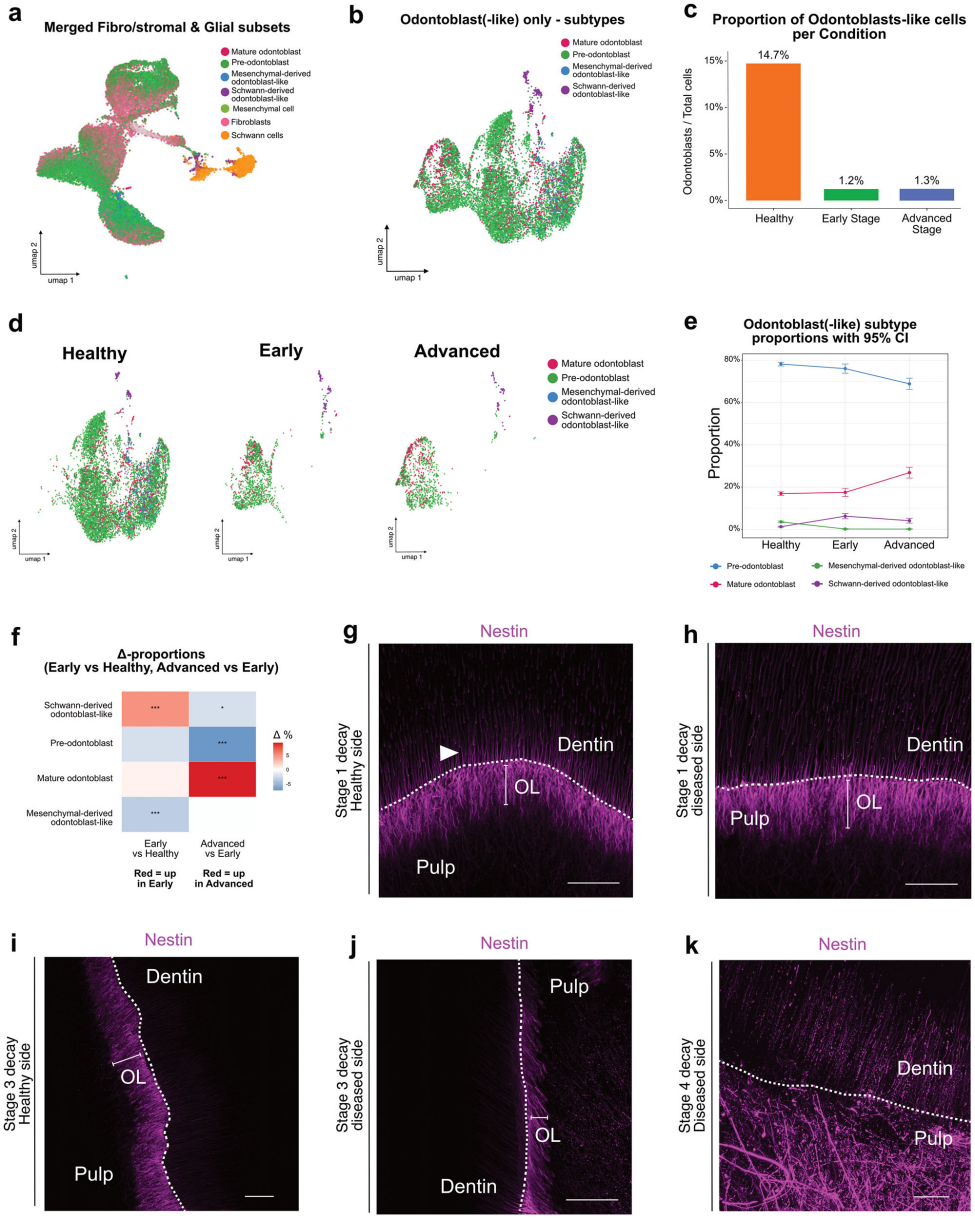
Evolution of Odontoblast (‐like) Subtypes Across Health and Disease Stages. a) UMAP representation of the merged fibro/stromal and glial subset showing the different subclusters. b) UMAP representation of the four odontoblastic subclusters: Mature odontoblast, preodontoblast, mesenchymal‐derived odontoblast‐like, and SC‐derived odontoblast‐like cells. c) Graph showing the proportions of odontoblast (‐like) cells across the three conditions. Data are normalized to the total cell number within each condition. d) UMAP representation of odontoblast(‐like) subtypes separated by health states (Healthy, Early, and Advanced stage). e) Proportions of odontoblast(‐like) subtypes across conditions with 95% confidence intervals. Pre‐odontoblasts constitute the largest population, while mature odontoblasts represent a minor but progressively increasing fraction from early to advanced stages. Mesenchymal‐ and SC‐derived odontoblast‐like cells remain very low. Proportion of odontoblast(‐like) subtypes per condition with 95% binomial Wald confidence intervals. Sample size (cells): Healthy *n* = 8100; Early *n* = 1480; Advanced *n* = 1158. No between‐condition hypothesis test is shown in this panel. f) Δ‐proportion heatmap of odontoblast(‐like) subtypes. Pairwise comparisons of subtype proportions between Early versus Healthy (right) and Advanced versus Early (left). Colors indicate the direction and magnitude of change (Δ%), with red showing enrichment and blue showing reduction in the numerator condition. SC‐derived odontoblast‐like cells increase significantly in the Early Stages compared to Healthy, but decline again in the Advanced Stages. Pre‐odontoblasts remain stable initially but decrease in Advanced stages. Mature odontoblasts are relatively preserved in Early and show a modest increase in Advanced. Mesenchymal‐derived odontoblast‐like cells decrease significantly already at the Early stage and remain unchanged thereafter. Significance stars are from two‐sided Fisher's exact tests on 2 × 2 contingency tables (subtype versus not‐subtype) for each contrast, with Benjamini–Hochberg FDR correction applied. Stars: *p* < 0.05 (*), <0.01 (**), <0.001 (***). Sample size (cells): Healthy *n* = 8100; Early *n* = 1480; Advanced *n* = 1158. g,h) Odontoblastic layers on a stage 1 decay (Early stage). g) Maximum intensity projection (MIP) of a 50 µm z‐stack on the healthy side, and h) of a 49 µm z‐stack on the side facing the decay. No major changes can be observed. i,j) Odontoblastic layer on a stage 3 decay (Advanced stage). i) MIP of a 49 µm z‐stack on the healthy side, and j) of a 49 µm z‐stack on the side facing the decay, showing a clear reduction in the odontoblastic layer thickness and reduction in the odontoblasts. k) MIP of a 131 µm z‐stack of the pulpo‐dentinal area on the side facing the decay, showing an absence of odontoblastic layer. Tissue clearing using a modified iDISCO protocol and staining for Nestin (purple). Point scanning resonant confocal microscope. Plan apo Lambda S40XC Sil. Optic for (g–j). Plan apo Lambda 25XC Sil. Optic for (k). Scale bars: 50 µm for (g–k). Copyright 2025, Hoang Thai HA.

As described above, odontoblast populations were identified in the integrated dataset and further characterized within both the fibro/stromal and glial subsets. Their dual developmental origin has been previously reported in the literature. To investigate their evolution during disease progression, we merged the fibro/stromal and glial subsets (**Figure** **14**a). We identified distinct odontoblast populations: mature odontoblasts (expressing *DSPP, DMP1, SPARC, COL1A1*), pre‐odontoblasts (expressing *RUNX2, MSX1, DLX3, PAX9, ALPL*), mesenchymal‐derived odontoblast‐like cells (expressing *PDGFRA, POSTN, THY1, ACTA2*) together with odontogenic genes (*DSPP, RUNX2*), and SC–derived odontoblast‐like cells (expressing *SOX10, MPZ, PLP1, S100B*) with co‐expression of odontoblast‐related genes (*DSPP, DPMP1, RUNX2*), along with mesenchymal cells (*ENG, NT5E, MCAM*), fibroblasts (*DCN, LUM, COL3A1*), and SCs (*NGFR, ERBB3, PLP1*). We then isolated the four odontoblastic subclusters (Figure 14b; Figure S9a–d, Supporting Information). A higher abundance of odontoblasts is observed in healthy pulps, with a drastic decrease at early stages and then a progressive decline into advanced disease stages (Figure 14c,d). Pre‐odontoblasts constituted the largest population, showing a marked reduction from healthy to diseased states. Mature odontoblasts, although less abundant, displayed a modest increase from the early to the advanced stages (Figure 14e). Differential proportion analysis confirmed the previous observations. SC‐derived odontoblast‐like cells significantly increased at the early stage before declining in advanced disease. Pre‐odontoblasts decreased consistently across the stages, while mature odontoblasts increased significantly from early to advanced stages. Mesenchymal‐derived odontoblast‐like cells were already reduced at the early stage and did not recover (Figure 14f).

Volumetric imaging analysis did not reveal significant changes in the odontoblastic layer at stage 1 decay (early stage), when comparing the healthy side of the pulp with the side adjacent to the lesion (Figure 14g,h). At the advanced stage (stage 3), however, an apparent reduction of the odontoblastic layer was observed on the lesion‐facing side (Figure 14i,j). In the most severe cases (stage 4), the odontoblastic layer was absent in areas directly facing the decay (Figure 14k).

**Figure 15:**
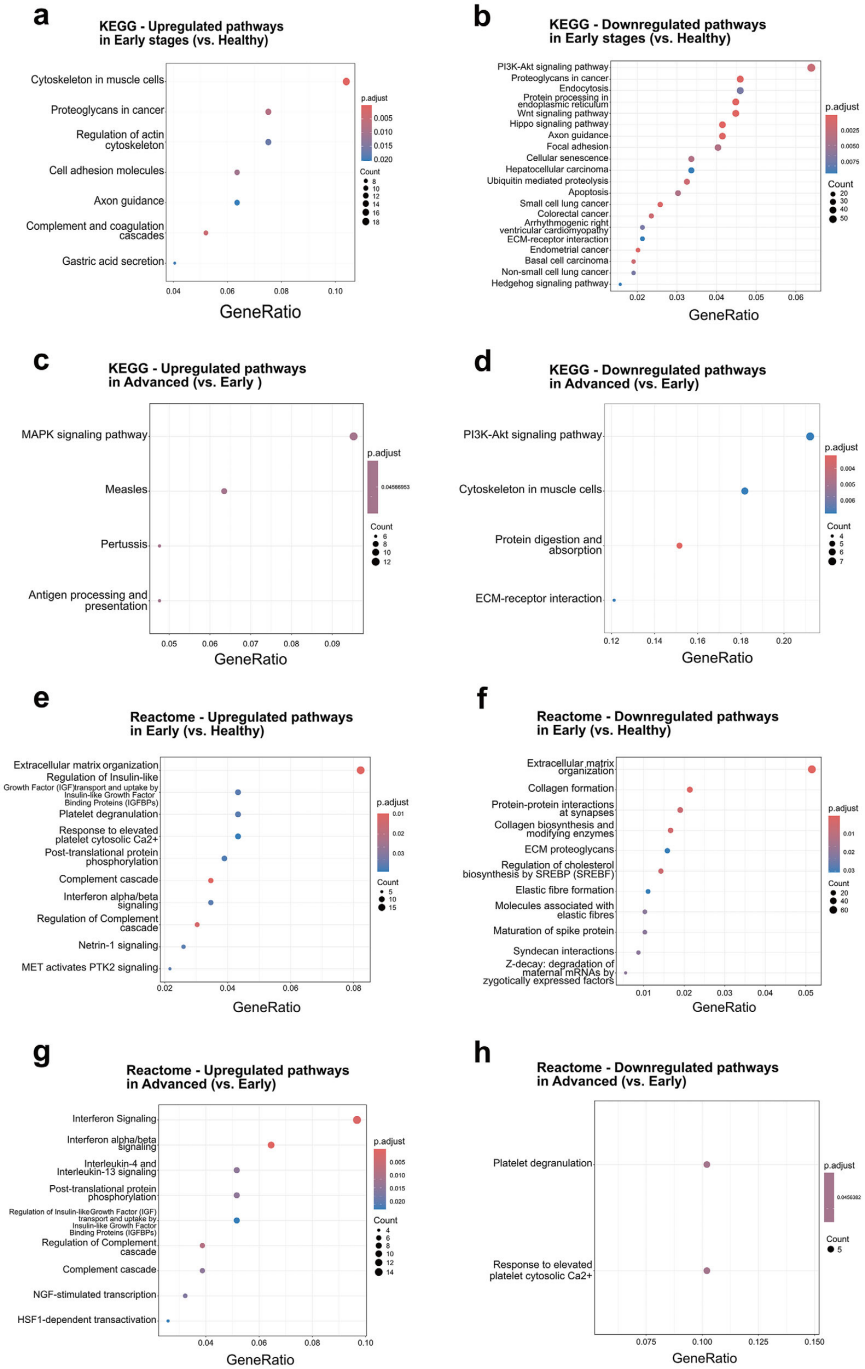
Functional Enrichment of Odontoblast (‐like) Cells Across Disease Stages. a,b) KEGG enrichment analysis for Early versus Healthy odontoblast(‐like) cells. Early odontoblasts exhibited a) significant upregulation of ECM remodeling, cytoskeletal organization, and immune‐related pathways, b) concomitant with downregulation of developmental and differentiation programs, including Wnt and Hippo signaling. c,d) Reactome enrichment analysis for Early versus Healthy. Early odontoblasts demonstrated (c) enrichment of ECM organization and complement cascade pathways, d) while processes related to collagen biosynthesis and fibril assembly were suppressed. Over‐representation analysis on differentially expressed genes (hypergeometric/Fisher's exact); *p* values BH‐adjusted (FDR). Sample size: number of mapped genes (upregulation, *n* = 377; downregulation, *n* = 2147). e,f) KEGG enrichment analysis for Advanced versus Early. Advanced odontoblasts exhibited e) a significant enrichment of MAPK signaling and antigen presentation pathways, f) whereas ECM–receptor interactions, PI3K–Akt signaling, and cytoskeletal organization were downregulated. g,h) Reactome enrichment analysis for Advanced versus Early. Advanced odontoblasts displayed g) upregulation of interferon α/β and cytokine signaling, including IL‐4 and IL‐13 pathways, h) together with downregulation of platelet degranulation and IGF2/SPARC‐mediated secretory functions. Over‐representation analysis on differentially expressed genes (hypergeometric/Fisher's exact); *p* values BH‐adjusted (FDR). Sample size: number of mapped genes (upregulation, *n* = 237; downregulation, *n* = 76).

To complete the cellular analysis, we performed pathway enrichment of odontoblast (‐like) cells across disease stages. In the Early versus Healthy comparison, KEGG analysis revealed upregulation of ECM remodeling, cytoskeletal regulation, and immune‐related processes (**Figure** **15**a). In contrast, developmental and differentiation programs, including Wnt and Hippo signaling, were significantly suppressed (Figure 15b). Reactome terms further confirmed the activation of ECM organization and complement signaling (Figure 15c), together with the downregulation of collagen formation and biosynthesis pathways (Figure 15d). In the comparison between the Advanced and Early stages, KEGG enrichment analysis indicated a strong induction of MAPK signaling and antigen presentation pathways (Figure 15e). In contrast, ECM‐receptor interactions, PI3K‐Akt survival signaling, and cytoskeletal programs were significantly reduced (Figure 15f). Reactome analysis revealed an upregulation of interferon α/β and cytokine signaling, including the IL‐4 and IL‐13 pathway (Figure 15g), alongside suppression of platelet degranulation and IGF2/SPARC‐mediated secretory function (Figure 15h). Collectively, these results indicate a progressive loss of odontoblast structural and secretory function alongside a gain of immune and stress‐related signatures.

## 3 Discussion

The tooth is composed of interconnected hard and soft tissues that interact dynamically at the dentin‐pulp interface, forming the dentin‐pulp complex.^[^ ^88^ ^]^ Our study aimed to elucidate the underappreciated impact of hard tissue (dentin) damage on distant soft tissue (dental pulp) during the natural course of the disease. Recently, several studies have investigated the cellular characteristics of the dental pulp under healthy conditions^[^ ^5^, ^6^, ^7^, ^8^ ^]^ and in disease states.^[^ ^12^, ^13^ ^]^ By integrating high‐resolution volumetric confocal imaging with in‐depth single‐cell analysis, we created a comprehensive imaging and cellular atlas of both healthy and diseased human teeth. Furthermore, our findings have significant global implications, as they reveal that dental pulp responses could mirror those observed in other tissues under pathological conditions, providing a potential novel human model to address those mechanisms.

Our panoramic approach highlights the intricate interplay between various systems within the dental pulp throughout disease progression, with the vasculature playing a pivotal role since most components involved in the inflammatory response transit through it.^[^ ^89^ ^]^ Early in the disease, bacterial invasion of the outer dentin layer is detected by specialized defense cells within the pulp, called odontoblasts, specifically through their odontoblastic processes. An arterialization of capillaries occurs at the localized inflamed site, recruiting α‐SMA^+^ cells driven by chemoattractants produced by stress‐activated ECs.^[^ ^90^ ^]^ Capillary and vessels lumen enlargement (outward remodeling) occurs as a mechanical response to the increased blood flow at the inflamed site, a process called arteriogenesis. Similarly, in the early stages of atherosclerosis, a radial expansion of vessels helps to preserve lumen size, thereby maintaining shear stress homeostasis.^[^ ^91^, ^92^ ^]^ The pulp's response to early decay has been documented for a long time.^[^ ^93^ ^]^ Numerous studies have noted changes in the dental pulp of teeth with early‐stage decay; however, these have often been limited to 2D histological analysis, while the pulp response is spread across the whole volume of the pulp.^[^ ^94^, ^95^ ^]^ Our study provides a deeper outlook on these early responses and provides a structural analysis across the pulp.

Inflammation serves as a self‐protective mechanism, promoting nerve and axon regeneration, as well as myelin formation.^[^ ^96^ ^]^ Our data demonstrate that neurogenesis, through nerve endings sprouting, increases within the odontoblastic layer at an early stage of the decay, explaining the increased tooth sensitivity the patient feels, as peripheral inflammatory mediators induce peripheral nerve sensitization.^[^ ^97^ ^]^ The myelinated SCs exhibit increased synthesis of NGF and serve as the primary mediators of this early neurogenesis. Additionally, Gli1^+^ perivascular progenitor cells rapidly differentiate into fibroblasts, thereby contributing to the pool of activated fibroblasts that are essential for tissue repair.^[^ ^98^ ^]^ These perivascular progenitor cells can also differentiate into odontoblast‐like cells and contribute to tooth defense by producing tertiary dentine. For this aspect, our data align with previous research.^[^ ^99^ ^]^ At these early stages of the disease (stages 1&2), the various remodeling processes contribute to tissue repair and aim for a return to homeostasis. Our data have significant clinical implications. At this stage, early lesions could be stabilized through remineralization if appropriate measures are taken, such as implementing a dental hygiene program, modifying the diet, and ensuring proper fluoride intake. If conservative treatment, such as placing a dental filling, is required, it will remove the demineralized tissue along with the microorganisms responsible for pulp inflammation. Our findings suggest that an early intervention on the affected mineralized tissues is essential to allow a natural repair of the underlying dental pulp. Further research is, however, needed to investigate the pulp's return to homeostasis.

Our data highlights the importance of containing the inflammatory process, as excessive and sustained inflammation elicits irreversible remodeling. As decay advances toward the dental pulp and the inflammation becomes chronic, an increasing number of mononuclear leukocytes (monocytes) are recruited to the inflamed and hypoxic tissue. These cells differentiate into macrophages, which promote the formation of new blood vessels, a process known as angiogenesis. As the formation of these de novo vessels originates from a pre‐existing capillary bed, this phenomenon is referred to as “sprouting angiogenesis”. It begins with the migration of endothelial cells, followed by the consolidation of endothelial cell junctions and the formation of a lumen. Tissue hypoxia, as seen in various diseases such as cancer or atherosclerosis, triggers this angiogenic switch.^[^ ^100^ ^]^ Our study shows a regression of vessels in the CP with the progression of the disease, leading to a lack of oxygen necessary for the odontoblast's activity. However, our data also shows that newly formed vessels are often non‐perfused and non‐functional due to the absence of endothelial junctions and lumens, therefore not perfusing the tissue, constituting pathological angiogenesis. It can be present in systemic diseases such as coronary artery disease or peripheral arterial disease (PAD), when the angiogenic response is insufficient to restore an adequate and functional vasculature.^[^ ^101^ ^]^ Persistent hypoxia and unresolved angiogenesis exacerbate immune cell infiltration, thereby precipitating tissue damage.^[^ ^102^ ^]^ We identified a subpopulation of B cells that is specifically increased in advanced stages and that might worsen the fibrosis. Chronic inflammation also increases peripheral nerve damage, releasing additional inflammatory mediators and perpetuating the inflammatory cycle.^[^ ^97^ ^]^ Simultaneously, activated fibroblasts increasingly differentiate into α‐SMA^+^ myofibroblasts, producing excessive ECM proteins, resulting in tissue fibrosis and degradation, as observed in many different tissues such as the heart or the lungs. At these advanced stages, merely removing the affected hard tissues, as is commonly done in clinical practice today, is insufficient to stop the progression of tissue remodeling and degradation. This ultimately leads to the need for complete dental pulp removal from the coronal and the root portions, replacing it with synthetic materials. This procedure, known as pulpectomy or root canal therapy, is commonly used to manage mature teeth with carious pulp involvement.^[^ ^103^ ^]^ In contrast, a pulpotomy involves only the partial removal of the dental pulp, limited to its coronal section, with the remaining tissue covered by a calcium silicate‐based cement. It is the recommended procedure for primary or immature teeth to preserve pulp vitality until root formation is complete, as well as for mature teeth with irreversible pulpitis.^[^ ^103^ ^]^ The successful outcome of this treatment relies solely on clinical and subjective criteria, such as the absence of pain and the lack of radiographic signs of peri‐apical lesions.^[^ ^103^ ^]^ In the event of a pulpectomy, whole pulp regeneration is a promising future therapy, not yet achievable. Our findings indicate that interventions at advanced disease stages require a multimodal approach. Restoring the affected hard tissues with dental materials should be complemented by strategies aimed at arresting and reversing dental pulp damage. These may include novel treatments such as angiogenesis inhibitors, antifibrotic drugs, or innovative regenerative therapies. These novel treatments associated with procedures such as a complete pulpotomy, which is the removal of the dental pulp in the coronal portion until the entry of the roots, or a partial pulpotomy, which is a partial removal (2‐3 mm) of the coronal pulp,^[^ ^104^ ^]^ could become a new standard of care. Indeed, root canal therapies that totally destroy the pulp tissue seem unnecessary and prevent the possibility of future pulp regeneration therapies. Further research is needed to investigate ways to promote pulp regeneration from the remaining living tissue, unaffected by fibrosis.

As previously mentioned, odontoblast (‐like) cells can have various origins. During tooth development, they are derived from the dental papilla mesenchyme, which originates from the neural crest cells.^[^ ^105^ ^]^ Neural crest cells are of ectodermal origin but undergo an epithelial‐to‐mesenchymal transition (EMT) during development,^[^ ^106^ ^]^ thereby acquiring mesenchymal properties. They lose their mesenchymal markers as they mature. In adults, they can have different sources: a (Gli1^+^) perivascular mesenchymal origin,^[^ ^99^, ^107^, ^108^, ^109^ ^]^ and a glial origin.^[^ ^40^, ^110^, ^111^ ^]^ Our study successfully identified specific odontoblast‐like populations in both the mesenchymal and the glial subsets, in alignment with the previous data. Moreover, our data do not indicate the activation of most genes associated with odontoblastic differentiation (*ALP, NES, AXIN2*). Furthermore, our data suggest that the Gli1^+^ perivascular cells primarily differentiate into fibroblasts and into mesenchymal‐derived odontoblast‐like cells at a very early stage of the decay, likely in response to the initial stromal damage. As shown in Figure 14e,f, the pre‐odontoblast population, which represents the major odontoblast population, decreases at early stages of decay and is likely replaced by newly differentiated Schwann cell‐derived odontoblast‐like cells. The latter showed a highly significant increase at the early stage, and might represent the primary source of odontoblast‐like cells. They, in turn, differentiate into mature odontoblasts. These findings provide a different perspective on the role of odontoblasts in the dental pulp defense mechanism. Advanced stages of the disease are characterized by a global reduction in the number of odontoblasts, especially under the decay location, further confirming the irreversibility of the damage. It remains to be seen whether neo‐odontoblasts (odontoblast‐like cells) can survive after the removal of decay, differentiate into mature odontoblasts, and restore the dentin‐pulp interface.

Untreated advanced dental decay is often linked to intensified and exacerbated pain. However, this clinical observation contrasts with our data, which show an increase in nerve fiber density (neurogenesis) only at early stages, followed by a gradual decline in later stages. These findings suggest the need to rethink current concepts of dental pain.^[^ ^112^ ^]^ Pain in the early stages is likely associated with increased nerve endings in RP, while pain in later stages may be linked to the autonomic nervous system and its connections to blood vessels that undergo outward remodeling in a confined space.

Unlike developing and immature dental pulp, which contains stem cells known as human dental pulp stem cells (hDPSCs), healthy mature dental pulp contains Gli+ perivascular progenitor cells and mesenchymal progenitor cells. Our findings reveal a drastic decline in Gli+ perivascular progenitor cells as soon as the disease begins, emphasizing the critical role of the vasculature in the immediate tissue response to inflammation. Additionally, it has been previously suggested that Gli1^+^ cells proliferate and differentiate into myofibroblast following injury to organs such as the heart, liver, lung or kidney.^[^ ^84^ ^]^ Our study revealed that disease progression to advanced stages leads to the establishment of a fibrotic condition, similar to other human organs.

In conclusion, our findings emphasize that dental decay is a slow‐progressing disease characterized by a continuum of transitional states within the pulp's cellular environment. These states involve multiple system interplays and a delicate balance between reversible reparative processes (e.g., outward remodeling and arterialization) and irreversible pathological mechanisms (e.g., pathological angiogenesis and fibrosis). The implications extend beyond dental sciences. In addition to establishing new standards of care for the dental profession, understanding these processes in the tooth will provide valuable insights into systemic conditions and offer a platform for testing innovative therapeutics.

## 4 Limitations of the Study

While this study provides for the first time a combined imaging and cellular insights into the biological response of the dental pulp to decay on untreated teeth, a limitation should be acknowledged. The healthy and diseased samples analyzed in this research were not always obtained from the same patient, which introduces variability due to individual genetic and physiological differences. An uncompleted tissue dissociation can also result in variation in cell proportion in the scRNA seq analysis.

## 5 Experimental Section

### Experimental Model and Study Participant Details—Human Patient Tissue Samples

Healthy and diseased human teeth were obtained from the Dentistry and Stomatology biobank at the Erasme University Hospital (HUB – Erasme site) (AFMPS number: BB190032). The samples were classified as residual human body material. They were collected from patients attending the Outpatient Dentistry Department and the One‐Day Clinic at the Erasme University Hospital (HUB – Erasme site, Brussels, Belgium). This study received approval from the Erasme Hospital‐Faculty Ethics Committee—ULB (Reference number P2021/146). All samples were anonymized to ensure patient identities were not accessible. The collection included healthy and diseased teeth from patients of both sexes, aged between 18 and 50 years, and representing various ethnicities (Table S1, Supporting Information).

The SiSta (Site & Stage) classification was used to determine the degree of progression in diseased teeth. The teeth were categorized into five groups:

1. Stage 0: representing a healthy tooth or a tooth with decay strictly limited to the enamel layer.
2. Stage 1: representing a tooth with decay limited to the external third of the dentin.
3. Stage 2: representing a tooth with decay limited to the middle third of the dentin.
4. Stage 3: representing a tooth with decay extending to the internal third of the dentin; and
5. Stage 4: representing a tooth with decay extending to the internal third and involving the dental pulp.

### Method Details—Human Patient Tissue Acquisition for Imaging

Samples from healthy and diseased teeth were obtained from the Dentistry and Stomatology biobank at Erasme University Hospital (Erasme site, Brussels, Belgium). The samples were fixed in a 4% formaldehyde solution immediately after extraction and stored at 4 °C for 7 to 14 days. The teeth were sectioned into 1 mm‐thick slices in the mesiodistal direction for healthy teeth and along the direction of decay for diseased teeth, using a gravity‐fed precision sectioning machine (Isomet Low Speed, Buehler, Germany) equipped with a precision diamond blade (IsoMet Diamond Wafering Blades, Buehler (Arbor Size: 0.5)). Sections containing both hard and soft tissues were preserved in Phosphate‐Buffered Saline (PBS) solution at 4 °C.

### iDISCO Clearing Protocol

The original iDISCO protocol^[^ ^14^ ^]^ was modified to suit our samples. Sectioned samples were first decalcified in ethylenediaminetetraacetic acid (EDTA) solution (0.5m, pH 7.0), which was renewed daily for a total of 7 days and kept on a shaker at 37 °C. They were washed in PBS solution for 1 day at room temperature. The samples were dehydrated at room temperature in methanol solutions diluted in PBS, as follows: 50% methanol for 1 h, 80% methanol for 1 h, and 100% methanol twice for 1 h each.

The samples were bleached overnight at 4 °C in a solution of 5% H_2_O_2_ in 20% DMSO/methanol, followed by washing steps at room temperature in 100% methanol twice for 1 h and in 20% DMSO/methanol twice for 1 h. The samples were rehydrated at room temperature as follows: 80% methanol for 1 h, 50% methanol for 1 h, and PBS twice for 1 h each. This was followed by a blocking step at room temperature twice for 1 h in PTx2 solution (PBS/0.2% Triton X‐100).

The immunostaining step began with overnight permeabilization at 37 °C in PTwH solution (1× PBS/0.2% Triton X‐100/20% DMSO/0.3m glycine). The samples were then blocked in Blocking Buffer solution (Intercept Blocking Buffer, Li‐Cor) containing 0.2% Triton X‐100 (Sigma–Aldrich) for 2 days and subsequently washed at room temperature twice for 1 h in PBS.

The samples were then incubated in the blocking buffer solution containing 0.2% Triton X‐100 and a primary antibody diluted at 1:1000 (1‰) on a shaker at 37 °C for 3 days, followed by washing in PBS solution for 1 day at room temperature. From this stage onward, the samples were protected from light using aluminum foil. The samples were then incubated in the blocking buffer solution containing 0.2% Triton X‐100 and a secondary antibody diluted at 1:1000 (1‰) on a shaker at 37 °C for 3 days, followed by washing in PBS solution for 1 day at room temperature.

The samples were dehydrated overnight at room temperature in a 50% v/v tetrahydrofuran/dH2O (THF) solution, then for 1 h in an 80% v/v THF/dH2O solution, and twice for 1 h in a 100% v/v THF solution. The samples were then incubated at room temperature in dichloromethane (DCM) until they sank to the bottom of the vial. Finally, the samples were cleared at room temperature in dibenzyl ether (DBE) until they were fully cleared and then stored in the same solution. All the steps were performed on a shaker.

### Microscopy and Image Analysis

Fluorescent images were acquired using two different microscopes. First, a scanning point resonant confocal microscope with a variable spectrum detector (Nikon AX R), equipped with various optics such as a Plan Apo 10x λS OFN25 DIC N1 and a Plan Apo Lambda 25xC Sil, was used to visualize the markings. Samples were mounted on Nunc Glass bottom dishes (Thermo Fisher Scientific, Massachusetts, USA) along with the storage solution. Second, a confocal microscope (Nikon) with a spinning disk (X‐Light V3, Crest Optics), connected to a camera (Photometrics Prime BSI), and equipped with a 10x immersion objective (N/A 0.5, WD 5.5 mm; Nikon Plan Apo), was also used to visualize the markings. Samples were mounted on Lab Glass Petri Dishes along with the storage solution. Z‐stacks of various steps and various depths (up to 600 µm) were acquired and processed using NIS software. The images were subsequently processed into MIP on which quantifications were performed.

### Human Patient Tissue Acquisition and Tissue Dissociation for Single‐Cell

Samples from healthy and diseased teeth were collected from patients attending the Outpatient Dentistry Department and the One‐Day Clinic at the Erasme University Hospital (HUB—Erasme site, Brussels, Belgium). Immediately after extraction, the samples were placed in Hank's Balanced Salt Solution (HBSS, Sigma‐Aldrich, St.Louis, USA) containing 1% Penicillin/Streptomycin/Amphotericin B solution (A5955‐100mL, Sigma‐Aldrich, St.Louis, USA) and kept on ice. All samples were processed and dissociated within 60 min of extraction.

Fresh collagenase solution for tissue dissociation was prepared by resuspending 40 mg of Collagenase Type 4 (Worthington Biochemical, NJ, USA) and 4 mg of calcium chloride in 10 mL of prewarmed TrypLE Express Enzyme 1X phenol red (Thermo Fisher Scientific, Massachusetts, USA) and maintained at 37 °C. Dissection forceps and scissors were disinfected using a VIRKON S solution (Germineo, Onet‐le‐Château, France). Petri dishes were filled with sterile, cold RPMI solution containing 1% penicillin, streptomycin, and amphotericin B.

To process the tooth, it was first held in place using a tooth holder, and the periodontium was scraped off with a surgical blade. The surface of the tooth was carefully wiped with 70% ethanol. The tooth was then cracked using a hammer, ensuring minimal compression to avoid tissue damage. The broken fragments were placed into a petri dish containing cold RPMI solution and kept on ice.

Under a stereomicroscope and using forceps, the fragments were carefully separated to expose the dental pulp. The pulp was then extracted from the pulp chamber, transferred to a fresh petri dish filled with cold RPMI/antibiotic‐antimycotic solution, and kept on ice. The dental pulp tissues were finely chopped into small pieces using fine scissors and immediately resuspended in a sterile 15 mL Falcon tube containing 3 mL of prewarmed collagenase solution.

The tubes were incubated at 37 °C for 50 min, with manual shaking performed every 10 min. After incubation, the tubes were placed on ice, and the tissue was manually triturated by pipetting up and down, first with a slightly cut P1000 tip and then with a standard P1000 tip, for 3 min until complete tissue disaggregation was achieved.

The cell suspension was filtered through a 40 µm cell strainer into a new 15 mL tube, and the filter was rinsed with 1 mL of RPMI solution. The tube was centrifuged at 500 g for 10 min in a swinging bucket rotor. The supernatant was carefully decanted, and the pellet was resuspended in 375 µL of cold Cell Prefixation Buffer (Parse Fixation Kit v2, Parse Biosciences, Seattle, USA).

Next, 125 µL of cold Cell Fixation Solution (ECF2101 Evercode Cell Fixation v2, Parse Biosciences, Seattle, USA) was added to the suspension, mixed by pipetting three times with a P1000 pipette, and incubated on ice for 10 min. Then, 40 µL of cold Cell Permeabilization Solution (ECF2101 Evercode Cell Fixation v2, Parse Biosciences, Seattle, USA) was added, mixed, and incubated on ice for 3 min. Finally, 500 µL of cold Cell Neutralization Buffer (ECF2101 Evercode Cell Fixation v2, Parse Biosciences, Seattle, USA) was added, and the tube was centrifuged at 500 × g for 10 min in a swinging bucket rotor.

The supernatant was carefully removed and discarded, and the pellet was resuspended in 75 µL of cold Cell Buffer (ECF2101 Evercode Cell Fixation v2, Parse Biosciences, Seattle, USA) and transferred to a 1.5 mL tube. A 10 µL aliquot of the cell suspension was taken for cell counting. This aliquot was mixed with 40 µL of 0.4% trypan blue, and the cells were counted using a Neubauer chamber.

To preserve the sample, 3.75 µL of DMSO (ECF2101 Evercode Cell Fixation v2, Parse Biosciences, Seattle, USA) was added, and the sample was stored in a Mr. Frosty freezing container at −80 °C for up to 6 months, until further processing for the single‐cell protocol was required.

### Single‐Cell Library Generation and Sequencing

Single‐cell sublibraries were generated following the Evercode WT v2 user manual v2.3 (ECW02130 Evercode WT v2, Parse Biosciences, Seattle, USA). Samples were thawed, counted, and recorded into the Sample Loading Table provided. Based on the Sample Loading Table values, samples were diluted with Dilution Buffer and loaded into the Round 1 Plate. During this step, an in‐situ reverse transcription reaction was performed, and well‐specific barcodes were added. The cells were then pooled, centrifuged, and resuspended.

The pooled cells were mixed with the Ligation Master Mix and transferred to the Round 2 Plate, where an in‐situ ligation reaction attached a second well‐specific barcode to the 3′ end of the cDNA. After pooling and straining, Round 3 Ligation Enzyme was added to the sample, which was then loaded into the Round 3 Plate. During this step, a second in situ ligation reaction introduced a third well‐specific barcode, the Illumina TruSeq R2 sequence, and a biotin to the cDNA. The sample was subsequently pooled, strained, centrifuged, washed, and resuspended in Dilution Buffer.

The cells were counted and allocated into sublibraries following the guidelines in the Sublibrary Generation Table. These sublibraries were lysed and stored at −80 °C until further processing. The barcoded cDNA was then captured on streptavidin‐coated magnetic Binder Beads and washed to remove cellular debris. After an additional wash, a template switching reaction was performed on the captured cDNA, adding a 5′ adaptor. The captured cDNA was further washed and amplified using primers targeting the template switching (TS) sequence and the Illumina TruSeq R2 sequence.

The amplified cDNA was purified using a 0.8x SPRI bead cleanup. Its concentration and size distribution were assessed using fluorescent dyes (Qubit dsDNA HS Assay Kit) and capillary electrophoresis (High Sensitivity DNA Kit on the Agilent Bioanalyzer System). The barcoded and amplified cDNA was subsequently fragmented, end‐repaired, and A‐tailed in a single reaction. Fragmented and A‐tailed DNA underwent size selection with sequential 0.6x and 0.8x SPRI bead cleanups. Adaptors containing the Illumina TruSeq R2 sequence were ligated to the 5′ end of the fragmented DNA, followed by another 0.8× SPRI bead cleanup.

Adaptor‐ligated DNA was PCR‐amplified using Illumina TruSeq R1 and R2 primers. This indexing PCR created sequencing libraries while adding i5/i7 unique dual indices (UDIs) as a fourth layer of cell barcoding. The sequencing libraries were size‐selected using a double‐sided SPRI bead cleanup. Concentration and size distribution were measured again using fluorescent dyes (Qubit dsDNA HS Assay Kit) and capillary electrophoresis (High Sensitivity DNA Kit on the Agilent Bioanalyzer System). Finally, the sublibraries were stored at 4 °C for up to 48 h or at −20 °C for up to 3 months.

Sublibraries were submitted for sequencing with the NovaSeq X Plus Series (PE150, Novogene UK, Cambridge, UK).

### Statistical Analysis—Pre‐Processing of Data

Sequenced data were processed using Trailmaker (Parse Biosciences, v1.5.0, Seattle, USA). FASTQ files were analysed through the Parse Bioscience pipeline to generate project‐specific insight modules for further ananlysis in Trailmaker. Data processing included: i) cell size distribution filter, ii) mitochondrial content filter, iii) number of genes versus transcripts filter, iv) doublet filter, v) Harmony‐based data integration, and vi) nonlinear dimensionality reduction (UMAP) to visualize clusters and their relative proximities. Clustering was performed using the Louvain method. The integrated data were log‐normalized before embedding. Trailmaker exported Seurat objects, which were then used for downstream analyses in R v4.4.3. Within Seurat (v5.3.0), counts were log‐normalized (LogNormalize, scale factor 10 000), scaled, reduced by PCA/UMAP, and clustered. Variable features were identified with variance‐stabilizing transformation. Outliers and doublets were excluded during quality control.

### Data Presentation

Data are shown as descriptive visualizations (UMAPs, chord diagrams, heatmaps, barplots, and dotplots) or statistical summaries (mean ± SD or mean per cluster ± 95% CI). Proportions are displayed with binomial Wald confidence intervals. Global communication metrics include 95% bootstrap confidence intervals. Volcano plots show log2 fold‐change versus ‐log10 adjusted *p*.

Morphological and histological quantifications (e.g., vascular remodeling parameters, pulp plexus thickness, tissue‐level measures) were performed on raw images using NIS Elements (Nikon, v5.42.03). Extracted values were exported and analyzed in GraphPad Prism v10.2.3, with results shown as mean ± SD, unless otherwise specified.

### Sample Size

Sample sizes are reported in figure legends. For the integrated dataset, numbers of cells per condition were: Healthy *n* = 30 786, Early *n* = 12 573, Advanced *n* = 10 048. Subset analyses included:

1. Immune cells: Healthy *n* = 2 745; Early *n* = 624; Advanced *n* = 986
2. Endothelial cells: Healthy *n* = 7 096; Early n = 4 711; Advanced *n* = 3 639
3. Fibro‐stromal cells: Healthy *n* = 19 117; Early *n* = 5 336; Advanced *n* = 3991
4. Glial cells: Healthy n = 664; Early n = 1 055; Advanced *n* = 731
5. Mural cells: Healthy *n* = 1094; Early *n* = 833; Advanced *n* = 689

For odontoblast(‐like) subtype analyses, totals were Healthy *n* = 8 100; Early *n* = 1480; Advanced *n* = 1 158. For enrichment analyses (GO, KEGG, Reactome), n = number of mapped DEGs tested: Early versus Healthy up *n* = 377, down *n* = 2147; Advanced versus Early up *n* = 237, down *n* = 76. For volcano plots, *n* = number of cells in the cluster versus comparators (e.g., B‐cells 3 *n* = 63 versus all other immune cells *n* = 4286).

### Statistical Methods

Differential expression was assessed using the two‐sided Wilcoxon rank‐sum test implemented in Seurat, applying thresholds of log2 fold‐change greater than 0.25 and a minimum expression fraction of 0.1. *P*‐values were adjusted for multiple testing using the Benjamini–Hochberg false discovery rate (FDR), and volcano plots display genes with adjusted *p* values below 0.05. Cell–cell communication significance was evaluated with the CellChat permutation test (*p* < 0.05, minimum of five cells per group). Global communication metrics such as the number of edges and summed interaction strength were further estimated by 500 bootstrap resamplings to derive 95% confidence intervals, while Δ circos plots are presented descriptively without additional hypothesis testing.

Proportional differences in odontoblast(‐like) subtypes were examined with overall chi‐square tests, and pairwise comparisons were performed using two‐sided Fisher's exact tests with Benjamini–Hochberg adjustment. Odds ratios and their 95% confidence intervals were estimated with the Haldane–Anscombe correction when applicable, and proportion curves are shown with 95% binomial Wald confidence intervals. Module scores generated in Seurat were compared across conditions using Kruskal–Wallis tests, followed by pairwise Wilcoxon tests with FDR correction.

Pathway activity was quantified with AUCell (v1.x), which computes the area under the recovery curve of ranked gene sets per cell, with values averaged by cluster. Pathway enrichment analyses (GO, KEGG, Reactome) were performed using over‐representation analysis based on the hypergeometric/Fisher's exact test, again with Benjamini–Hochberg FDR correction. For Reactome, cnetplots are used to display the links between significant genes and enriched pathways. Protein–protein interaction networks were constructed from upregulated DEGs using STRING references; these networks are descriptive, with hubs and modules defined by graph topology measures such as node degree and clustering.

Finally, image‐based quantifications were carried out in NIS Elements (Nikon, v5.42.03), and the exported numerical values were analyzed in GraphPad Prism (v10.2.3). Depending on data distribution and design, two‐sided Student's *t* tests were applied for normally distributed data with equal variance, one‐way or two‐way ANOVA with Tukey's or Sidak's post‐hoc corrections for comparisons among multiple groups, or non‐parametric tests such as Mann–Whitney or Kruskal–Wallis with Dunn's correction when assumptions were not met. All tests were two‐sided, and statistical significance was defined as *p* < 0.05.

### Softwares

FASTQ preprocessing: Trailmaker pipeline v1.5.0 (Parse Biosciences). Image quantification: NIS Elements v5.42.03 (Nikon). Downstream analysis: R v4.4.3 with Seurat v5.3.0, SeuratObject v5.1.0, CellChat v1.6.1, clusterProfiler v4.14.6, ReactomePA v1.50.0, org.Hs.eg.db v3.20.0, AUCell v1.28.0, STRINGdb v2.18.0, enrichplot v1.26.6, DOSE v4.0.1, fgsea v1.32.4, ggplot2 v3.5.2, cowplot v1.2.0, pheatmap v1.0.13. Quantification/statistics: GraphPad Prism v10.2.3. Figures exported as SVG.

## Conflict of Interest

The authors declare no conflict of interest.

## Supporting information



Supporting Information

Supporting Information

## Data Availability

Raw (FASTQ files) and processed data from the single‐cell experiment have been deposited in ArrayExpress (a functional genomic data collection) and are publicly available as of the date of publication (ArrayExpress accession E‐MTAB‐14896).
