# Supplementary material for: Human Atlas of Tooth Decay Progression: Identification of Cellular Mechanisms Driving the Switch from Dental Pulp Repair Toward Irreversible Pulpitis
Source: Adv Sci (Weinh). 2025 Oct 31;13(8):e10096. doi: 10.1002/advs.202510096 (PMC12884820; doi:10.1002/advs.202510096)
Supplement: Supplementary file 1 — Supporting Information [file ADVS-13-e10096-s002.docx]

Supporting Information

**Human Atlas of Tooth Decay Progression: Identification of Cellular Mechanisms Driving the Switch from Dental Pulp Repair Towards Irreversible Pulpitis**

*Hoang-Thai HA ^*^, Sofya KOSMYNINA, Amandine VEROCQ, Keremsah OZEN, Ines TEKIA, Hugo BUSSY, Marie RAMIREZ, Dima SABBAH, Chloe GOEMANS, Valerie VANDENBEMPT, Esteban GURZOV, Sumeet Pal SINGH, Nicolas BAEYENS ^*^*

**Video S1. Representative 3D Rendering Demonstrating the Intimate Association Between Nerves and Vasculature in the Dental Pulp.**


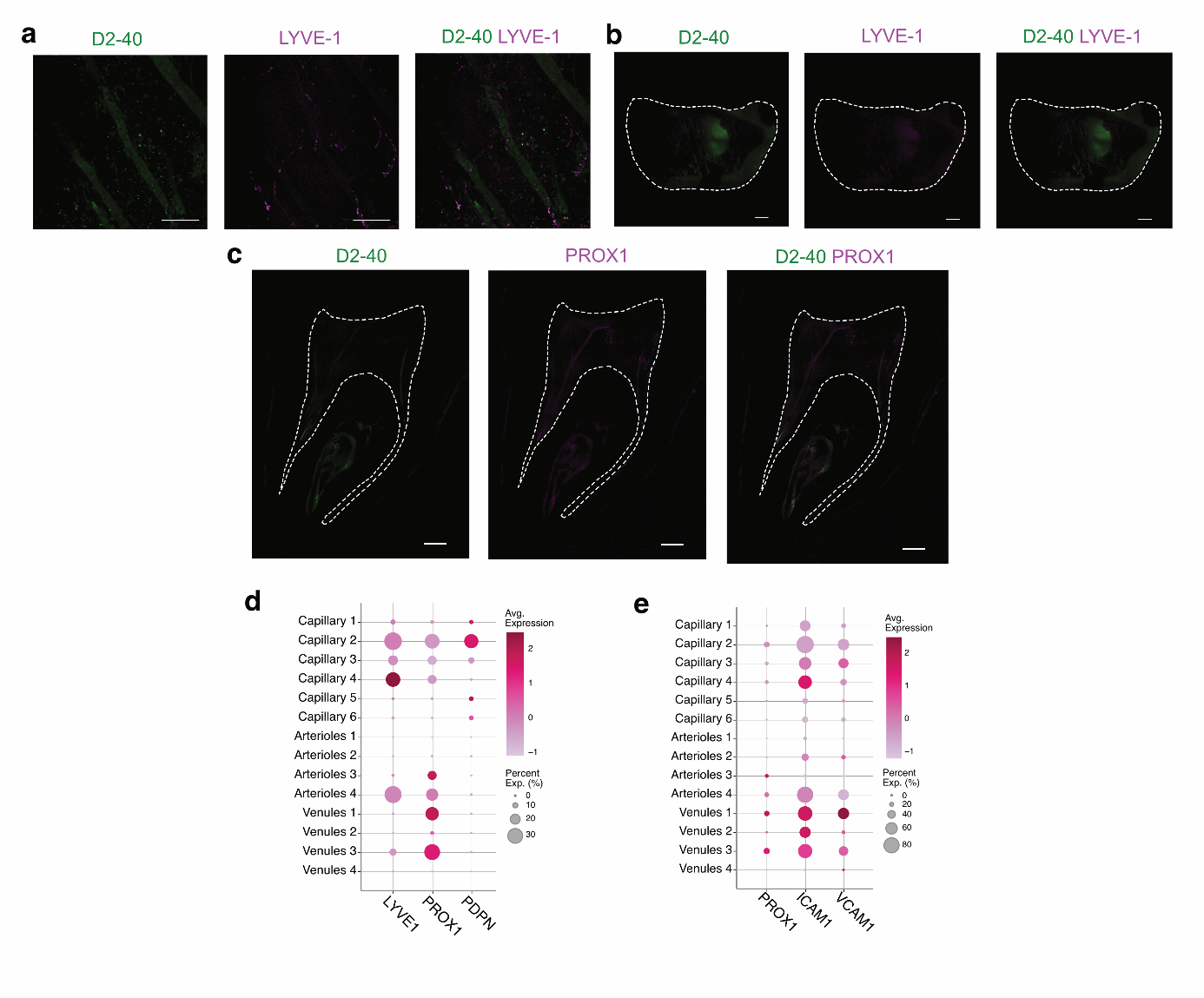


**Figure S1. Absence of Detectable Lymphatic Vessel Signal in Human Dental Pulp.**

(a-c) MIP of (a) a 57µm z-stack, (b) a 161µm z-stack, and (c) a 141µm z-stack showing the absence of immunolabeling for lymphatic structures.

Tissue transparization using a modified iDISCO protocol and staining for the lymphatic vessels (a-b) with D2-40 (green) and LYVE1 (purple), and (b) with D2-40 (green) and PROX1 (purple). Point scanning resonant confocal microscope. Plan apo Lambda 25XC Sil. Optic for (a). Plan Apo 10x λS OFN25 DIC N1 optic for (b, c). Scale bars: 1000µm for (c), 500µm for (b, c), 50µm for (a).

(d) Dot plot showing 3 well-established cell type markers for lymphatic vessels.

(e) Dot plot showing co-expression of a well-established marker for lymphatic vessels (PROX1) and 2 markers associated with reactive post-capillary venules (REVs).


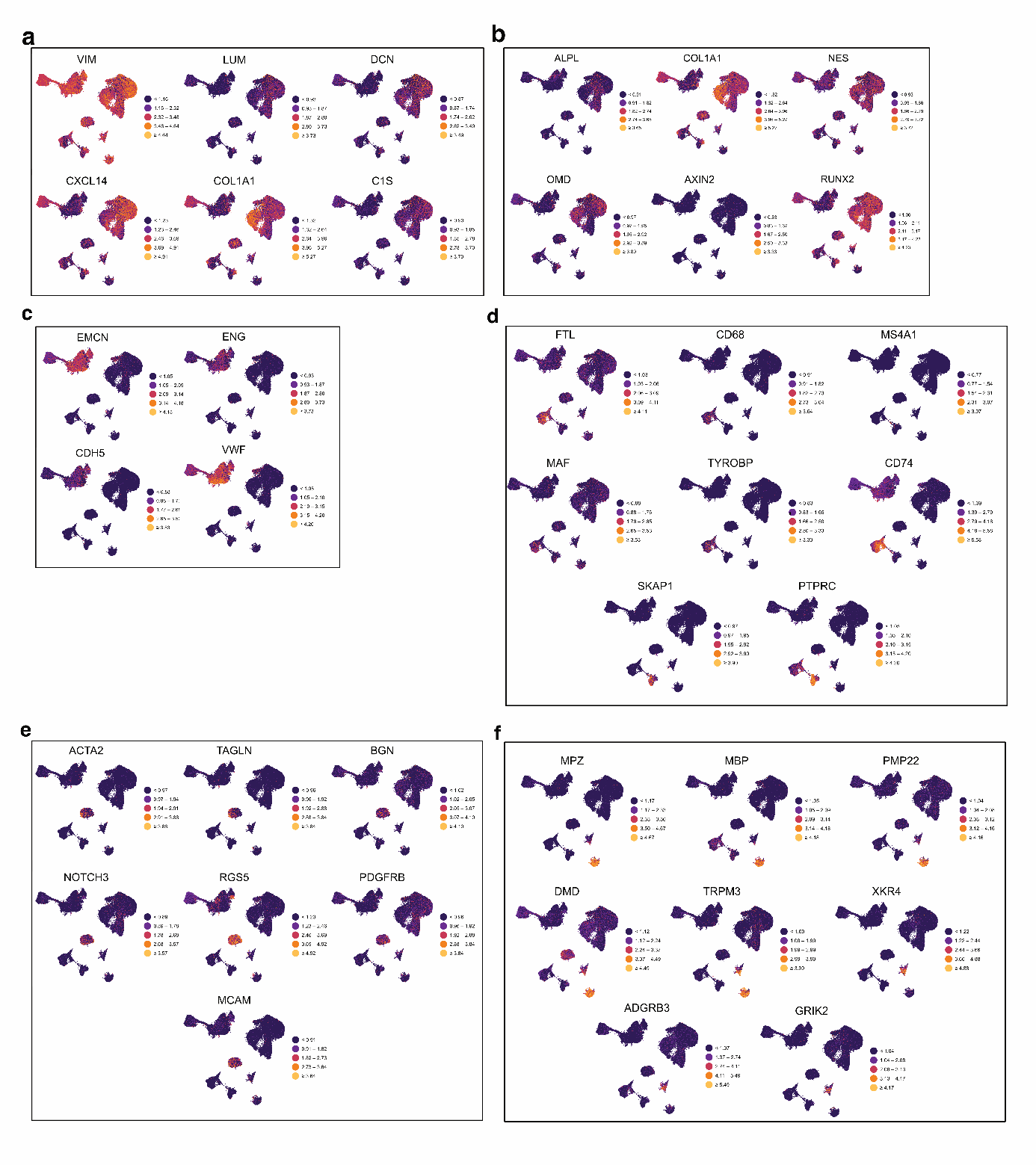


**Figure S2. UMAP Visualization of Lineage-Specific Marker Gene Expression in Single-cell Populations of Human Dental Pulp.**

(a) Fibroblast/stromal markers (*VIM, LUM, DCN, CXCL14, COL1A1, C1S*). (b) Odontoblast markers (*ALPL, COL1A1, NES, OMD, AXIN2, RUNX2*). (c) Endothelial cell markers (*EMCN, ENG, CDH5, VWF*). (d) Immune cell markers (*FTL, CD68, MS4A1, MAF, TYROBP, CD74, SKAP1, PTPRC*). (e) Mural cell markers (*ACTA2, TAGLN, BGN, NOTCH3, RGSS, PDGFRB, MCAM*). (f) Glial cell markers (*MPZ, MBP, PMP22, DMD, TRPM3, XKR4, ADGRB3, GRIK2*). Each UMAP shows the distribution and expression intensity of the indicated marker, confirming the presence of distinct stromal, odontoblastic, vascular, immune, mural, and glial cell populations within the dental pulp.


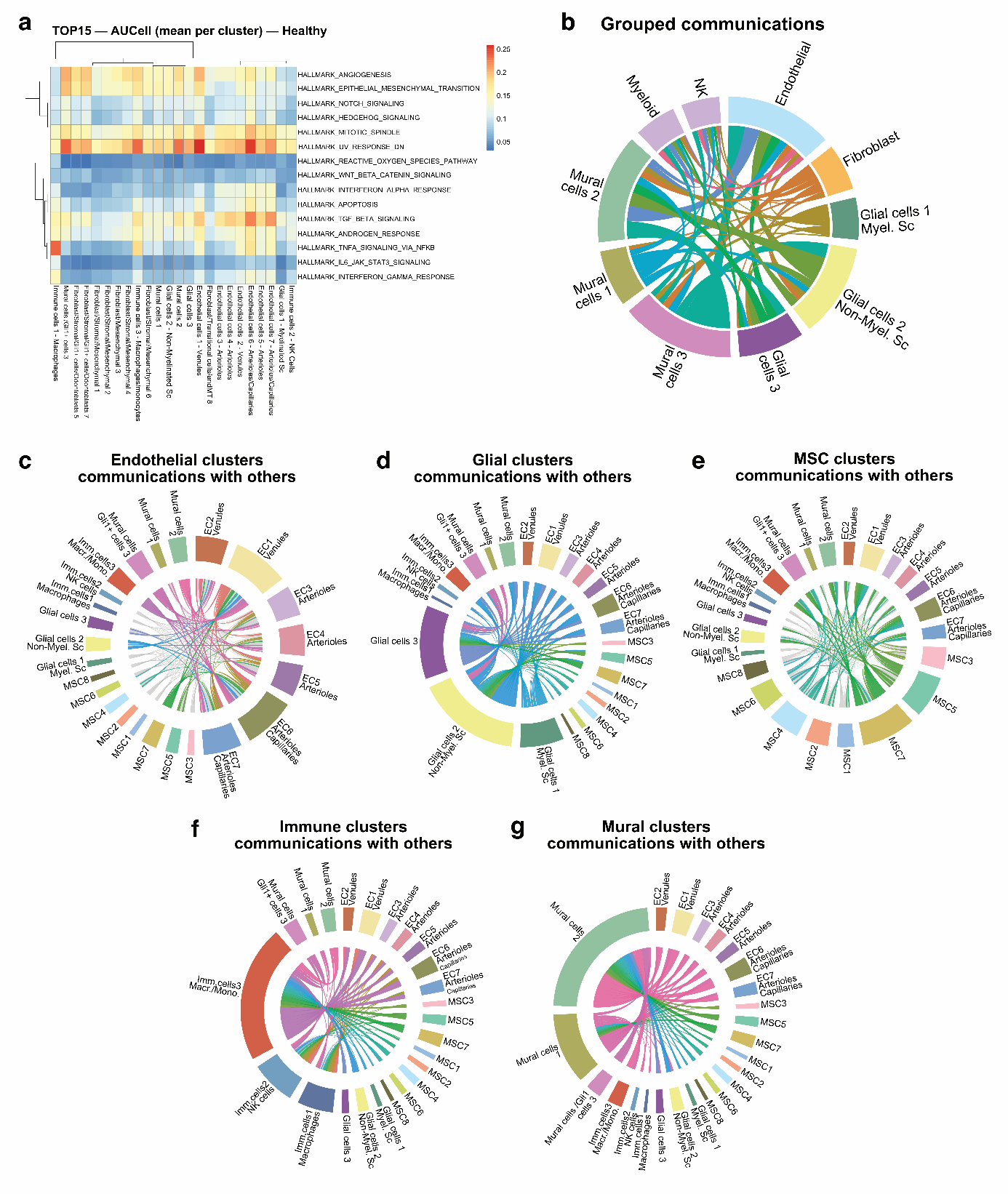


**Figure S3. Pathway Enrichment and Intercellular Communication in Healthy Dental Pulp.**

(a) Heatmap showing the top 15 enriched Hallmark pathways (AUCell scores, mean per cluster) in the Healthy condition. Stromal/mesenchymal/odontoblast populations are enriched for ECM and mesenchymal signaling programs. ECs exhibit angiogenesis-related activity, immune subsets display interferon and inflammatory responses, whereas glial populations show lower enrichment. AUCell scores averaged per cluster; heatmap clustered by correlation (complete linkage). Descriptive only, no statistical test. Sample size: Healthy n = 30 786 (95–6 789 per cluster). (b) Grouped chord diagram summarizing population-level communications highlights reciprocal interactions between stromal, vascular, mural, glial, and immune compartments. Edges = significant ligand–receptor interactions (CellChat permutation test, p < 0.05; min.cells = 5); edge width = summed communication probability; nodes grouped by compartment. Descriptive summary (no extra tests). Sample size (integrated): Healthy n = 30 786. (c–g) Subpopulation-specific chord diagrams detail communication patterns: (c) endothelial clusters preferentially interact with stromal and mural subsets, (d) glial clusters communicate mainly with stromal and vascular populations, (e) stromal/MSC clusters act as central hubs with broad interactions, (f) immune clusters show more restricted communications toward stromal and endothelial subsets, and (g) mural clusters primarily connect with endothelial and stromal populations. For (c-g) Edges = significant ligand–receptor interactions (CellChat permutation test, p < 0.05; min.cells = 5); edge width = summed communication probability. Descriptive only (no additional test). Sample size (Healthy condition, integrated dataset): n = 30 786 cells.


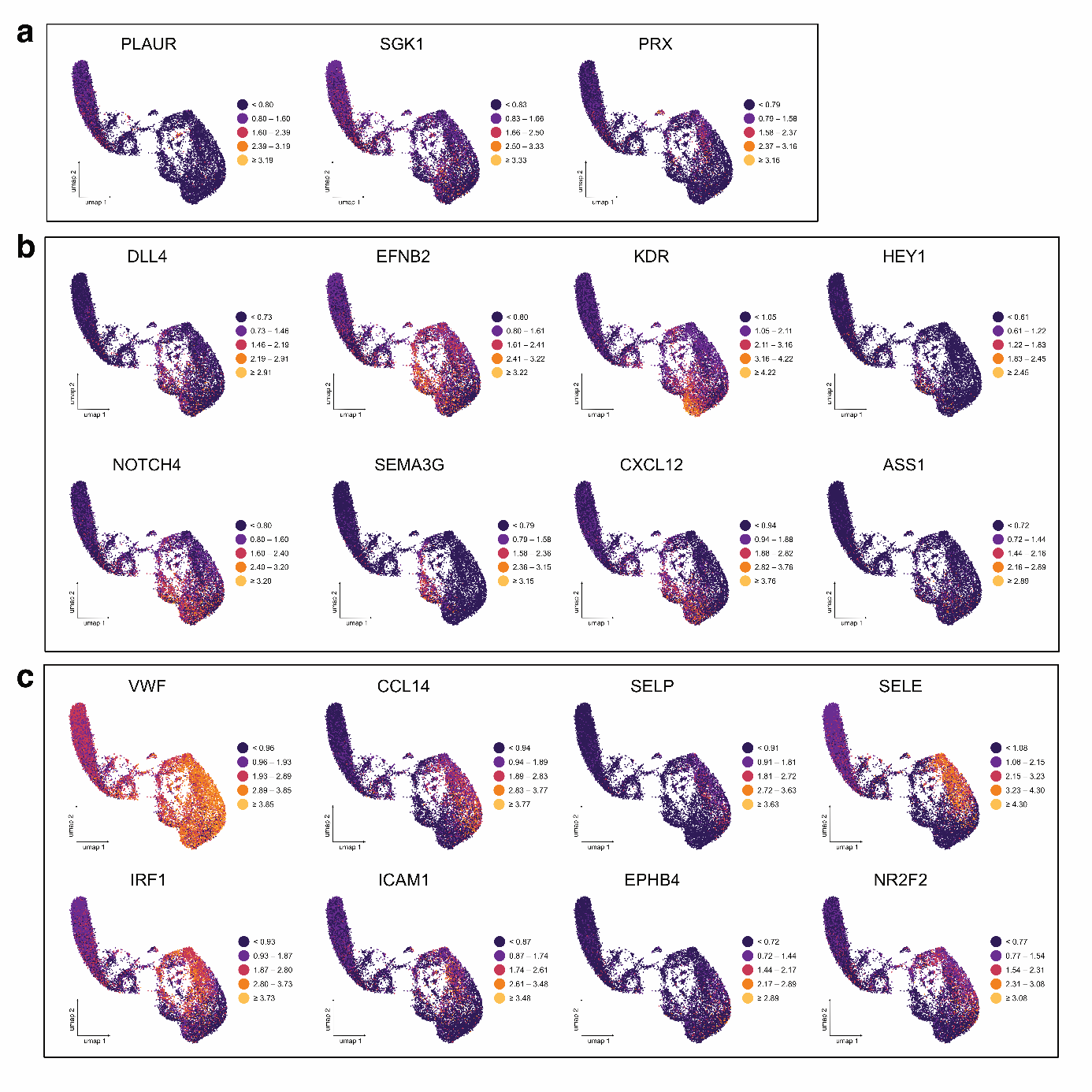


**Figure S4. UMAP Visualization of EC Subclusters and Marker Gene Expression.**
(a) Capillary EC subclusters, marked by high expression of *PLAUR, SGK1*, and *PRX*. (b) Arteriolar EC subclusters, identified by expression of *DLL4, EFNB2, KDR, HEY1, NOTCH4, SEMA3G, CXCL12, and ASS1*. (c) Venous EC subclusters, characterized by expression of *VWF, CCL14, SELP, SELE, IRF1, ICAM1, EPHB4, and NR2F2*. UMAP plots illustrate the spatial distribution and relative expression levels of these markers across the fourteen endothelial subclusters (six capillary, four arteriolar, and four venous).


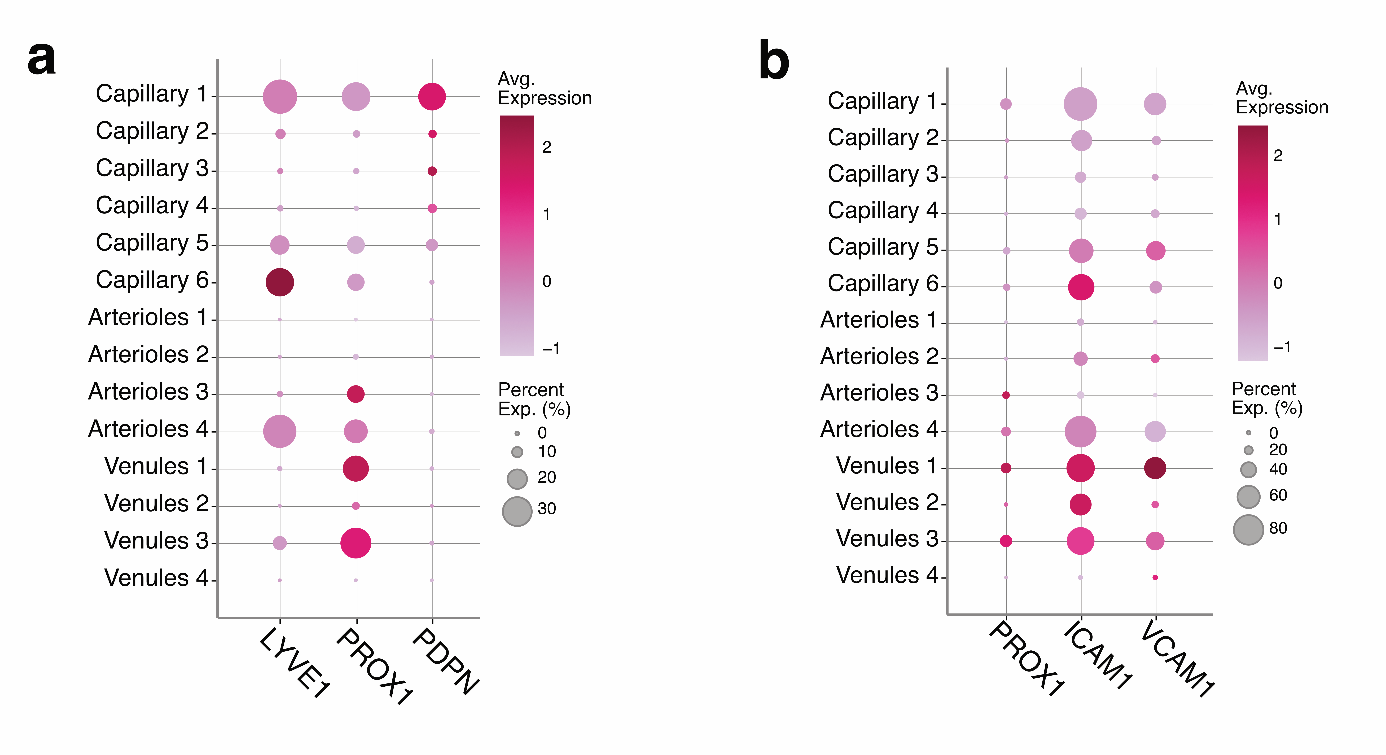


**Figure S5. Dot Plot Analysis of Lymphatic and Venous Marker Expression in Endothelial Subclusters.**

(a) Expression of canonical lymphatic markers (*LYVE1, PROX1, PDPN*) across endothelial subclusters shows no evidence of a distinct lymphatic population. (b) Venule 1 subcluster exhibits co-expression of *PROX1, ICAM1, and VCAM1*, consistent with the presence of specialized reactive post-capillary venules (REVs). Dot size indicates the percentage of cells expressing each marker, while color reflects the average expression level.


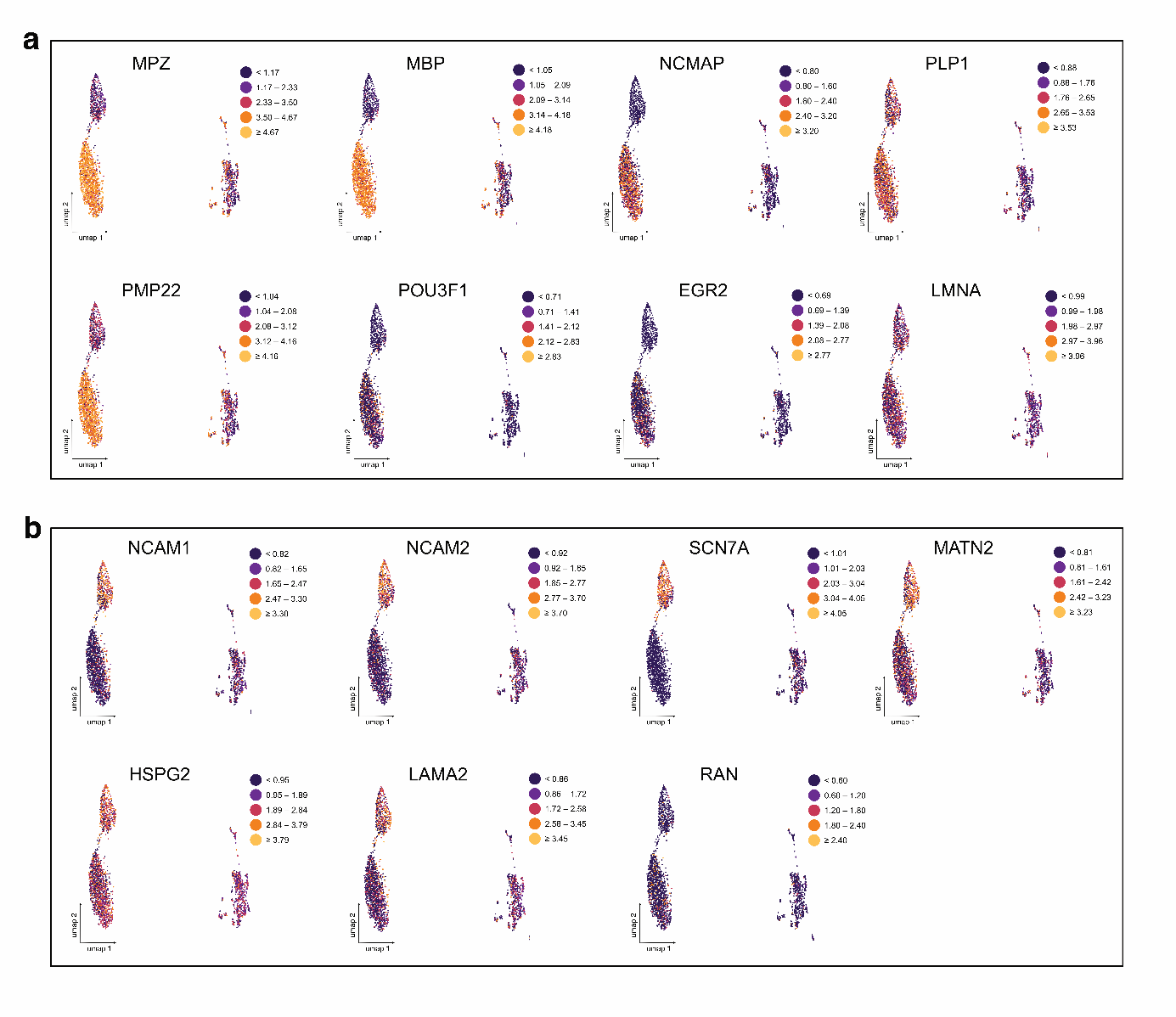


**Figure S6. UMAP Visualization of Glial Cell Subclusters and Marker Gene Expression.**
UMAP plots illustrate the distribution and relative expression intensity of each marker across glial subclusters. (a) Two subclusters of myelinated SCs defined by expression of *MPZ, MBP, NCMAP, PLP1, POU3F1, EGR2, and LMNA*. (b) Two subclusters of non-myelinated SCs are characterized by the expression of *NCAM1, NCAM2, SCN7A, MATN2, HSPG2, LAMA2, and RAN*.


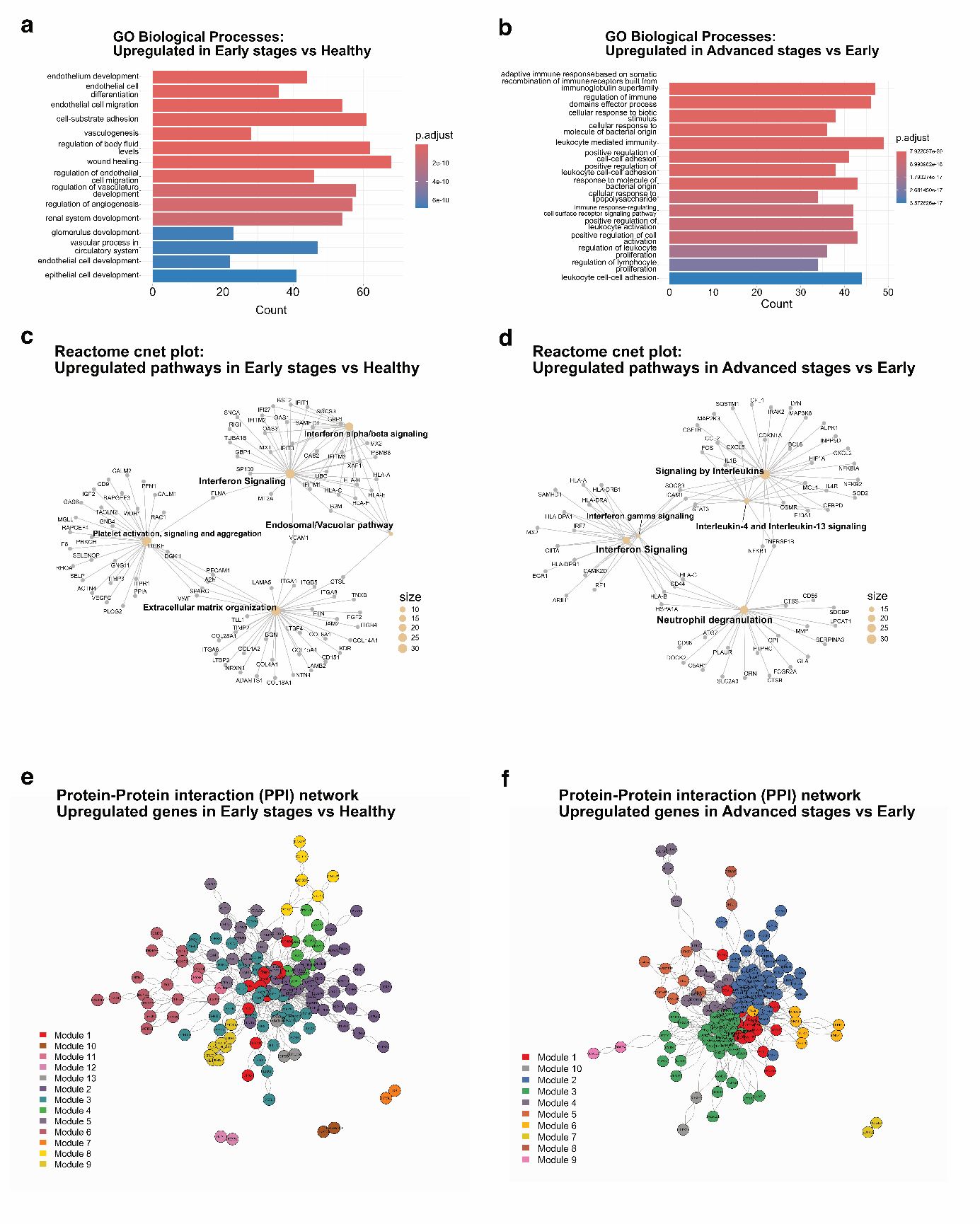


**Figure S7. Complementary Enrichment and PPI-Network Analyses Across Stages.**

(a) **Early vs Healthy (GO** **Biological Processes, upregulated in Early stages):** enrichment for endothelium development, cell–substrate adhesion, vasculogenesis, and regulation of angiogenesis. (b) **Advanced vs Early (GO Biological Processes, upregulated in Advanced stages):** enrichment for immune activation and leukocyte-mediated responses, with additional metabolic processes. (c) **Early vs Healthy (Reactome cnet, upregulated in Early stages):** nodes linking platelet activation/signaling/aggregation with ECM organization, endosomal/vacuolar pathways, and interferon (α/β) signaling. (d) **Advanced vs Early (Reactome cnet, upregulated in advanced stages):** signaling by interleukins (including IL-4/IL-7), neutrophil degranulation, and interferon-γ/type I interferon pathways, consistent with inflammatory activation. For (a-d) Over-representation analysis (hypergeometric/Fisher’s exact) with Benjamini–Hochberg FDR; cnetplots link significant genes to enriched pathways. Sample size (n): upregulated mapped genes — Early vs Healthy n = 377; Advanced vs Early n = 237. (e) **Early vs Healthy (PPI, upregulated in Early stages):** the network resolves modules M1 (Blood coagulation), M2 (Vasculogenesis), M3 (Muscle cell differentiation), M4 (Endodermal cell differentiation), M5 (Microvillus organization), and M6 (Alternative mRNA splicing), along with smaller unannotated clusters (M7–M13). (f) Advanced vs. Early (PPI, upregulated in Advanced stages): modules include M1 (Antigen processing/presentation), M2 (Regulation of inflammatory response), M3 (Microglial cell activation), and M4 (L-leucine transport), plus smaller unannotated clusters (M5–M10). For (e-f) Networks were built from upregulated DEGs using STRING-based protein–protein interactions. Nodes = upregulated mapped genes; edges = high-confidence interactions. The DEG lists were obtained with a Wilcoxon rank-sum test (p < 0.05, log2FC > 0.25). The PPI plots are descriptive; no additional statistical test was applied at the network level. Sample size (n): upregulated mapped genes — Early vs Healthy n = 377; Advanced vs Early n = 237.


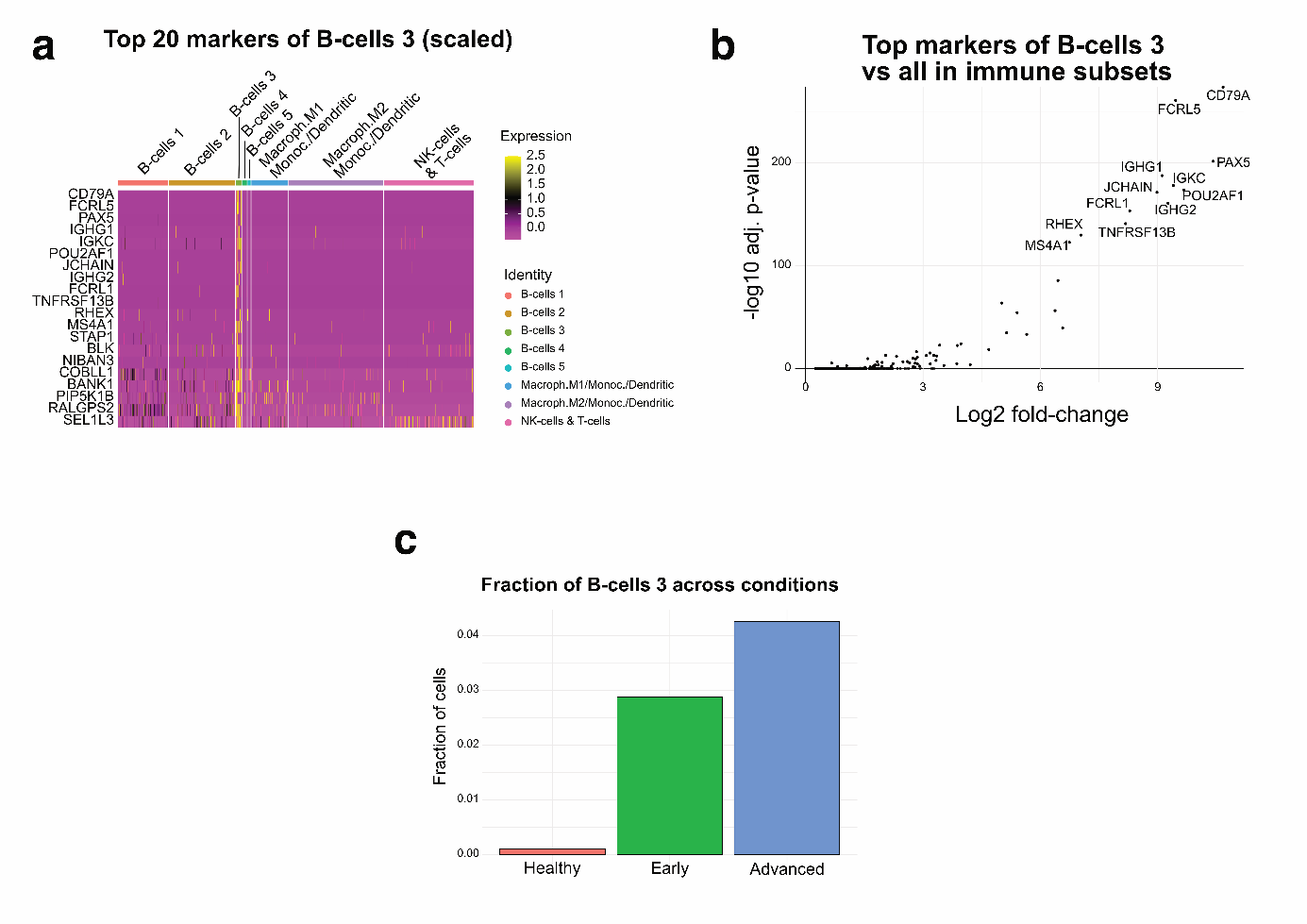


**Figure S8. Characterization and Stage-Specific Abundance of the B-cells 3 Subcluster.**
(a) Heatmap of the top 20 marker genes (scaled) across immune subclusters showing selective expression in B-cells 3. (b) Differential expression of B-cells 3 versus all other immune cells in the dedicated subset (log₂ fold-change vs −log₁₀ adjusted P), highlighting canonical B-cell markers (*CD79A, MS4A1/CD20, PAX5, BLK, POU2AF1*) and immunoglobulin/activation-associated genes (*IGKC, IGHG1/2, JCHAIN, FCRL5, TNFRSF13B*), consistent with an activated B-cell phenotype. Differential expression was tested using the Wilcoxon rank-sum test with Benjamini–Hochberg FDR correction. Genes were considered significant at log2FC > 0.25 and adjusted p < 0.05. Sample size: B-cells 3 (n = 63) vs all other immune subsets (n = 4 286). (c) Fraction of B-cells 3 across conditions, showing low frequency in Healthy, a notable increase in Early stages, and a gradual increase to Advanced stages.


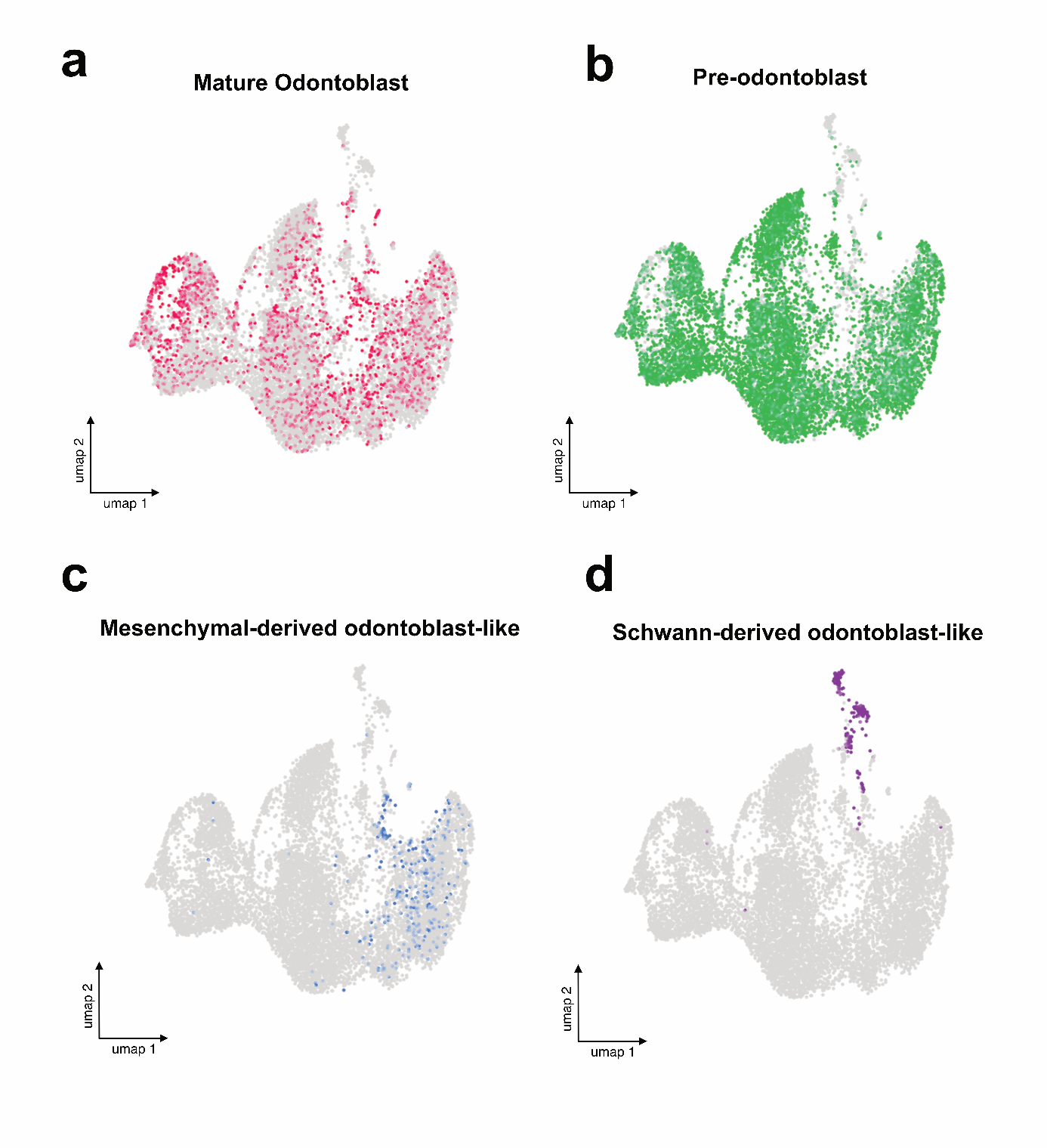


**Figure S9. UMAP Representations of Odontoblast (-like) Subtypes in the Merged Fibro/Stromal and Glial Dataset.**

(a) Mature odontoblast (expression of *DSPP, DMP1, SPARC, and* COL1A1). (b) Preodontoblasts (expression of *RUNX2, MSX1,* DLX3, *DLX2, PAX9, and ALPL*). (c) Mesenchymal-derived odontoblast-like cells identified by co-expression of mesenchymal markers (*PDGFRA, POSTN, THY1, and ACTA2*) together with odontogenic genes (*DSPP, RUNX2*). (d) SC-derived odontoblast-like cells, defined by SC markers (*SOX10, MPZ, PLP1, S100B*) with co-expression of odontoblast-related genes (*DSPP*, *DPMP1, RUNX2*). Each panel highlights the distribution of one subtype (colored) relative to all other cells in grey.

**Table S1. Data on Samples for the Single-cell Experiment.** This table details sample information, including name, health state, tooth number, sex, age, total cell counts, and cell concentration after tissue dissociation and fixation.

| Sample name | Health state | Tooth number | Sex | Age | Total cell count | Cell count/uL |
| --- | --- | --- | --- | --- | --- | --- |
| Healthy 1 | Healthy | 38 | F | 18 | 119 250 | 795 |
| Healthy 2 | Healthy | 28 | M | 18 | 228 375 | 3 045 |
| Healthy 3 | Healthy | 48 | F | 28 | 30.750 | 410 |
| Healthy 4 | Healthy | 18 | F | 28 | 24 750 | 330 |
| Early-stage 1 | Stage 1-2 | 38 | F | 19 | 83 250 | 555 |
| Early-stage 2 | Stage 2 | 38 | M | 31 | 77 250 | 1 030 |
| Early-stage 3 | Stage 1-2 | 28 | F | 30 | 20 625 | 275 |
| Early-stage 4 | Stage 2 | 18 | M | 27 | 24 750 | 330 |
| Advanced stage 1 | Stage 3 | 28 | M | 29 | 28 125 | 375 |
| Advanced stage 2 | Stage 3 | 48 | M | 31 | 111 375 | 1 485 |
| Advanced stage 3 | Stage 4 | 38 | F | 30 | 25 500 | 340 |
| Advanced stage 4 | Stage 4 | 28 | M | 27 | 37 125 | 495 |

**Table S2. Key Resources Table**

| REAGENT or RESOURCE | SOURCE | IDENTIFIER |
| --- | --- | --- |
| Antibodies | | |
| Polyclonal Goat IgG Human VE‑Cadherin Antibody | R&D Systems, Inc. | AF938, RRID: AB_355726 |
| Mouse monoclonal Anti-α Smooth Muscle Actin | Sigma-Aldrich | Cat# A2547, RRID: AB_476701, Cat# A5228, RRID: AB_262054 |
| Neurofilament-H Rabbit monoclonal Ab | Cell signaling | Cat #30564 |
| Nestin Monoclonal Antibody | Thermo Fisher Scientific | Cat# 14-9843-80, RRID: AB_1548838 |
| Nestin Antibody | Cell signaling | Cat#10959 |
| UCHL1/PGP9.5 antibody | Proteintech | Cat# 14730-1-AP, RRID: AB_2210497 |
| Podoplanin antibody | Proteintech | Cat# 67432-1-Ig RRID: AB_2882670 |
| Human LYVE-1 Antibody | R&D Systems, Inc. | AF2089, RRID: AB_355144 |
| PROX1 Polyclonal antibody | Proteintech | Cat# 11067-2-AP, RRID: AB_2268804 |
| Donkey anti-Goat IgG (H+L) Highly Cross-Adsorbed Secondary Antibody, Alexa Fluor™ Plus 555 | Thermo Fisher Scientific | Cat# A32816, RRID: AB_2762839 |
| Donkey Anti-Mouse IgG H&L (Alexa Fluor® 647) | Abcam | Cat# ab150107, RRID: AB_2890037 |
| Donkey anti-Rabbit IgG (H+L) Highly Cross-Adsorbed Secondary Antibody, Alexa Fluor™ Plus 555 | Thermo Fisher Scientific | Cat# A32794, RRID: AB_2762834 |
| Goat anti-Mouse IgG (H+L) Highly Cross-Adsorbed Secondary Antibody, Alexa Fluor™ Plus 555 | Thermo Fisher Scientific | Cat# A32727, RRID: AB_2633276 |
| Donkey anti-Rabbit IgG (H+L) Highly Cross-Adsorbed Secondary Antibody, Alexa Fluor™ Plus 647 | Thermo Fisher Scientific | Cat# A32795, RRID: AB_2762835 |
| Donkey anti-Mouse IgG (H+L) Highly Cross-Adsorbed Secondary Antibody, Alexa Fluor™ Plus 647 | Thermo Fisher Scientific | Cat# A32787, RRID: AB_2762830 |
| Biological Samples |  |  |
| Healthy and diseased adult human teeth | The Dentistry and Stomatology biobank at the Erasme University Hospital (HUB - Erasme site) (AFMPS number: BB190032) | https://www.erasme.be/fr/enseignement-recherche/comite-d-ethique/biobanques-cbeu/les-biobanques-sur-le-campus-erasme |
|  |  |  |
| Chemicals, Peptides, and Recombinant Proteins | | |
| Ethylenediaminetetraacetic acid disodium salt dihydrate | Sigma-Aldrich | ED2SS-1KG |
| Methanol | Sigma-Aldrich | 179957-1L |
| Hydrogen peroxide solution 30% | Sigma-Aldrich | 216763-100ML |
| DMSO | Sigma-Aldrich | D128-4 |
| Triton X-100 | Sigma-Aldrich | X100-500ML |
| Tetrahydrofuran THF | Sigma-Aldrich | 186562 |
| Dichloromethane (DCM) | Sigma-Aldrich | 270997 |
| Dibenzylether (DBE) | Sigma-Aldrich | 108014-1KG |
| Nunc Glass base dish 27mm | ThermoFisher | 150682 |
| Intercept® (PBS) Blocking Buffer | LI-COR Biosciences - GmbH | 927-70001 |
| Collagenase Type 4 | Worthington  Biochemical Corp. | LS004210 |
| Hank's Balanced Salt Solution | Sigma-Aldrich | H9394 |
| RPMI 1640 Medium, no glutamine, no phenol red 500 mL | ThermoFisher | 32404014 |
| TrypLE™ Express Enzyme (1X), phenol red | ThermoFisher | 12605010 |
| Cell strainer | VWR | 732-2758 |
| Antibiotic Antimycotic Solution (100×), Stabilized | Sigma-Aldrich | A5955-20ML |
| KAPA Pure Beads (5ml) | Roche | KK8000 (07983271001) |
|  |  |  |
| Critical Commercial Assays | | |
| Evercode™ Cell Fixation v2 | Parse biosciences | Ref# ECF2101 |
| Evercode™ WT v2 | Parse biosciences | Ref# ECW02130 |
| UDI Plate - WT | Parse biosciences | Ref# UDI1001 |
| Qubit™ dsDNA HS Assay Kit | ThermoFisher | Q32851 |
| Bioanalyzer High Sensitivity DNA Analysis | Aligent | 5067-4626; 5067-4627 |
|  |  |  |
| Software and Algorithms | | |
| NIS-Elements | NIKON CORPORATION | v5.42.03  https://www.microscope.healthcare.nikon.com/en_EU/products/software/nis-elements |
| GraphPad Prism 9 | GraphPad Software | v10.2.3  https://www.graphpad.com/updates/prism-900-release-notes |
| Trailmaker™ | Parse biosciences | Pipeline v1.5.0  https://www.parsebiosciences.com/data-analysis/ |
| Seurat | Satija Lab | v5.3.0 |
| CellChat R Package | Jin et al., 2021; available at https://github.com/sqjin/CellChat | V1.6.1 ; CellChatDB.human (human) |
|  |  |  |
| Other | | |
| Isomet Low Speed® | Buehler | https://www.buehler.com/products/sectioning/precision-cutters/isomet-low-speed-precision-cutter/ |
| Scanning point resonant confocal microscope with a variable spectrum detector (Nikon AX R) | Nikon | https://www.microscope.healthcare.nikon.com/en_EU/products/confocal-microscopes/ax |
| Confocal microscope with a spinning disk (X-Light V3, Crest Optics), | Nikon | https://www.microscope.healthcare.nikon.com/en_EU/products/confocal-microscopes/crest-x-light-series/crest-x-light-v3 |
| 10x immersion objective (N/A 0.5, WD 5.5 mm; Nikon Plan Apo) | Nikon | https://www.microscope.healthcare.nikon.com/en_EU/products/optics/selector |
| Plan Apo 10x λS OFN25 DIC N1 optics | Nikon | https://www.microscope.healthcare.nikon.com/en_EU/products/optics/selector |
| Plan Apo Lambda 25xC Sil | Nikon | https://www.microscope.healthcare.nikon.com/en_EU/products/optics/selector |
| Qubit™ Flex Fluorometer | ThermoFisher | https://www.thermofisher.com/be/en/home/industrial/spectroscopy-elemental-isotope-analysis/molecular-spectroscopy/fluorometers/qubit.html |
| 2100 Bioanalyzer | Agilent | https://www.agilent.com/en/product/automated-electrophoresis/bioanalyzer-systems/bioanalyzer-instrument |
|  |  |  |
